# Supplementary material for: Safety and effectiveness of hormonal vs non-hormonal or no contraception in women with hypertension and future fertility desire: A broad-scope systematic review
Source: PLoS One. 2026 Mar 31;21(3):e0345959. doi: 10.1371/journal.pone.0345959 (PMC13038026; doi:10.1371/journal.pone.0345959)
Supplement: S1 Appendix — (PDF) [file pone.0345959.s001.pdf]

A. Appendix S1: *Classification of hormonal contraceptive methods, percentages of unwanted pregnancies with perfect and typical use during the first year and side effects and side effects* [8,97,98]

**Classification and description of contraceptive methods**				**Percentage of unwanted pregnancies**		**Side effects**
**Contraceptive group**	**Description**	**Contraceptive method**	**Main action mechanism**	**Perfect use**	**Typical use**	
Combined hormonal contraceptives	Methods containing ethinyl estradiol and a progestin (8)	Combined oral contraceptives (COCs)	Inhibits ovulation [98]	0.3%	9%	Changes in menstruation, headache, nausea, changes in weight, acne, increased blood pressure [98] and increased risk of thromboembolic disease [30,99,100]
		Combined patch	Inhibits ovulation [98]	0.3%	9%	Skin irritation at the patch location, changes in menstruation, headache, nausea, vomiting, vaginitis, and tenderness and pain in the breasts [98].
		Combined vaginal ring	Inhibits ovulation [98]	0.3%	9%	Changes in the menstruation, headache and vaginitis [98].
		Combined injectable	Inhibits ovulation [98]	0.05%	3%	Changes in menstruation, weight gain, headache and breast tenderness [98].

Classification and description of contraceptive methods				Percentage of unwanted pregnancies		Side effects
**Contraceptive group**	**Description**	**Contraceptive method**	**Main action mechanism**	**Perfect use**	**Typical use**	
Progestin-only contraceptives	Contraceptives that only contain a progestin (8).	Progestin-only pill (POP)	Inhibits ovulation and thickens cervical mucus [98].	0.3%	9%	Changes in menstruation, headache, nausea and changes in mood [98].
		Levonorgestrel and etonogestrel implants	It inhibits ovulation, thickens cervical mucus and suppresses cyclic activity of the endometrium [98].	0.05%	0,05%	Changes in menstruation, headache, abdominal pain, acne, weight changes and nausea [98].
		Medroxyprogesterone acetate injection and norethisterone enanthate injection	Inhibits ovulation and increases the density of cervical mucus [98].	0.2%	4%	Changes in menstruation, weight gain, changes in mood and bone density loss [98].
Emergency contraceptive pills	They are used after having relationships sexual without using any contraceptive method. Includes: OCP, levonorgestrel contraceptive and ulipristal acetate (8).	Levonorgestrel contraceptive	Avoid a retreat to ovulation and hinder the passage of sperm to the ovum [98,101]	1%	1%	Changes in menstruation, nausea, abdominal pain, fatigue and headache [98].
		Ulipristal acetate		<1%	<1%	
Levonorgestrel-releasing intrauterine device	Plastic T that continuously releases intrauterine doses of levonorgestrel (8).	Intrauterine device (IUD) with levonorgestrel	Increases the density of cervical mucus [98], reduces estrogen and progesterone receptors in the endometrium and exerts an antiproliferative effect [102].	0.2%	0.2%	Changes in menstrual periods, acne, headache, tenderness in the breasts, nausea, weight gain, and mood changes [98].
