# Supplementary material for: Safety and effectiveness of hormonal vs non-hormonal or no contraception in women with hypertension and future fertility desire: A broad-scope systematic review
Source: PLoS One. 2026 Mar 31;21(3):e0345959. doi: 10.1371/journal.pone.0345959 (PMC13038026; doi:10.1371/journal.pone.0345959)
Supplement: S2 Appendix — (PDF) [file pone.0345959.s002.pdf]

B. **Appendix S2:** ***Classification of non-hormonal contraceptive methods, percentage of unwanted pregnancies in the first year according to the hormonal contraceptive method with perfect and typical use, designed from information in*** **[8,97]**

Group	Contraceptive method	Perfect use	Typical use	Side effects
Intrauterine device (IUD)	Copper IUD	0.6%	0.8%	Most are harmless, although changes in the menstrual cycle, increased risk of pelvic inflammatory disease (PID), uterine perforation, and increased risk of vaginal infections have been identified with the copper IUD [98].
Barrier methods	Male condom	2%	18%	
	Female condom	5%	21%	
	Spermicides	18%	28%	
	Diaphragms	6%	12%	
Natural methods	Lactational Amenorrhea	0.9%	-	
	Fixed days method	5%	-	
	Reversing method	4%	-	
	Rhythm method	No reliable data	-	
