# Supplementary material for: Safety and effectiveness of hormonal vs non-hormonal or no contraception in women with hypertension and future fertility desire: A broad-scope systematic review
Source: PLoS One. 2026 Mar 31;21(3):e0345959. doi: 10.1371/journal.pone.0345959 (PMC13038026; doi:10.1371/journal.pone.0345959)
Supplement: S3 Appendix — (PDF) [file pone.0345959.s003.pdf]

C. Appendix S3: PRISMA 2020 Checklist

Section and Topic	Item	Checklist item	Location where item is reported	Brief description
**Title**				
Title	1	Identify the report as a systematic review.	Page 1	Safety and effectiveness of hormonal contraception methods versus non-hormonal contraception methods or non-use of contraception methods for women of reproductive age with desire for future fertility and hypertension: A systematic review of broad scope
**Abstract**				
Abstract	2	See the PRISMA 2020 for Abstracts checklist.	Appendix D	Appendix D
**Introduction**				
Rationale	3	Describe the rationale for the review in the context of existing knowledge.	Page 2.	Hypertensive women need safe contraceptive methods. Hormonal methods are very effective, but could increase cardiovascular risk in women at high risk. There are two critically low-quality reviews on the safety of combined oral contraceptives in hypertensive women, and current World Health Organization (WHO) recommendations are based on individual studies and consensus. Given this lack of quality evidence, there is a need for a systematic review on the safety and effectiveness of hormonal contraceptives in hypertensive women, allowing for improvements in the decisions made or continuation of current practices.
Objectives	4	Provide an explicit statement of the objective(s) or question(s) the review addresses.	Page 3.	This systematic review seeks to evaluate the safety and effectiveness of hormonal versus non-hormonal contraceptive methods or non-use of contraception in women of reproductive age with a desire for future fertility and high blood pressure.

Section and Topic	Item	Checklist item	Location where item is reported	Brief description
**Methods**				
Eligibility criteria	5	Specify the inclusion and exclusion criteria for the review and how studies were grouped for the syntheses.	Page 3-4	Inclusion criteria: Studies assessing the safety ((clinical trials (RCTs), cohort, case-control, case reports, case series, clinical trial records, and adverse event reports) or effectiveness (RCTs and cohort studies) of hormonal contraception in hypertensive women of reproductive age (15–49 years) desiring future fertility.<br>Exclusion criteria: Studies exclusively comparing hormonal methods, and those including women with polycystic ovary syndrome, abnormal uterine bleeding, heavy menstrual bleeding, or early ovarian failure (hormone replacement).<br><br>The evidence was synthesized according to the type of hormonal contraceptive and outcome quantitatively (association estimator, vote count and meta-analysis) and qualitatively (narrative synthesis) in cases where it was not possible to perform meta-analysis. The outcomes were presented by type of hormonal contraceptive and by type of study.
Information sources	6	Specify all databases, registers, websites, organisations, reference lists and other sources searched or consulted to identify studies. Specify the date when each source was last searched or consulted.	Page 5	An initial search was conducted between September 29 and October 4, 2022, followed by a first update between September 12 and 13, 2023 and a second update between August 7 and 8, 2024, in the databases Medline (via Ovid), Embase, Cochrane Central Registry of Controlled Trials (CENTRAL) and the database of Latin American and Caribbean Literature in Life Sciences Health (LILACS). Additionally, on June 27, 2022, August 26 and 27, 2023, and September 11 and 12, 2024, searches were carried out in the registries of clinical trials, regulatory agencies, and databases specialized in reporting adverse events. , post-marketing safety and gray literature bases.

Section and Topic	Item	Checklist item	Location where item is reported	Brief description
Search strategy	7	Present the full search strategies for all databases, registers and websites, including any filters and limits used.	Appendix G	Appendix G.
Selection process	8	Specify the methods used to decide whether a study met the inclusion criteria of the review, including how many reviewers screened each record and each report retrieved, whether they worked independently, and if applicable, details of automation tools used in the process.	Page 5	Two reviewers (NL, AB, JV, AR) independently evaluated titles and abstracts, following the eligibility criteria contemplated in the protocol. The selected studies were reviewed in full text by two reviewers (NL and AB), independently. In case of discrepancies, a third reviewer (KE) was consulted.
Data collection process	9	Specify the methods used to collect data from reports, including how many reviewers collected data from each report, whether	Page 6.	Information from the included full-text studies was independently extracted by two reviewers (NL, AB, MH, PG) on the REDCap platform. Data extraction was performed independently. When differences occurred in the extracted data, they were resolved through dialogue and reviewing the evaluated study jointly. One reviewer (NL) transferred the collected data to Review Manager 5.4.1.

Section and Topic	Item	Checklist item	Location where item is reported	Brief description
		they worked independently, any processes for obtaining or confirming data from study investigators, and if applicable, details of automation tools used in the process.		
Data items	10a	List and define all outcomes for which data were sought. Specify whether all results that were compatible with each outcome domain in each study were sought (e.g. for all measures, time points, analyses), and if not, the methods used to decide which results to collect.	Page 4	The primary outcomes considered were: MACE and unwanted pregnancies. Secondary outcomes included: Pearl index, pelvic inflammatory disease (PID), vaginal infections, loss of fertility, discontinuation of contraception due to side effects or interactions with medications for chronic diseases, worsening of underlying medical condition, arterial disease peripheral, venous thromboembolism, weight gain and alteration in liver or kidney function tests.
	10b	List and define all other variables for which data were sought (e.g. participant and intervention	Page 6-7	The items on the data extraction forms for each study design are found in Appendix I.<br><br>In the presence of missing data, we sought to contact the authors of the studies to recover the information.

Section and Topic	Item	Checklist item	Location where item is reported	Brief description
		characteristics, funding sources). Describe any assumptions made about any missing or unclear information.		
Study risk of bias assessment	11	Specify the methods used to assess risk of bias in the included studies, including details of the tool(s) used, how many reviewers assessed each study and whether they worked independently, and if applicable, details of automation tools used in the process.	Page 6	Two reviewers (NL, AB, MH, PG) independently assessed the risk of bias in the cohort studies, the methodological quality of the case-control studies, and the critical approach of the included case series studies. This information was compiled in REDCap. Any disagreements were resolved through dialogue.<br><br>To assess the risk of bias in cohort studies, the ROBINS-I tool was used, for case-control studies, methodological quality was assessed by using the Newcastle-Ottawa tool and case series studies. The critical approach tool of the Joanna Briggs Institute was used. Assessment of the risk of bias by outcome reported in the different types of study designs was carried out using the GRADE approach.
Effect measures	12	Specify for each outcome the effect measure(s) (e.g. risk ratio, mean difference) used in the synthesis or presentation of results.	Page 6-7	For the dichotomous primary and secondary outcomes, we sought to use estimators adjusted for possible confounding variables that would present the relationship between the exposure and the outcome in hypertensive women (the hypertensive population in both the numerator and the denominator). In the absence of these, crude Odds Ratios (OR) were calculated along with their respective confidence intervals as measures of association. ORs were estimated instead of RR since it is a more stable

Section and Topic	Item	Checklist item	Location where item is reported	Brief description
				measure of association, and since the outcomes evaluated are rare in the population of interest, the OR does not overestimate the association. If the study did not report or a measure of association could not be calculated, narrative synthesis was performed. For continuous outcomes, narrative synthesis was performed.
Synthesis methods	13a	Describe the processes used to decide which studies were eligible for each synthesis (e.g. tabulating the study intervention characteristics and comparing against the planned groups for each synthesis (item #5)).	Page 8-9	The evidence was synthesized according to the type of hormonal contraceptive and outcome quantitatively (association estimator, vote count and meta-analysis) and qualitatively (narrative synthesis) in cases where it was not possible to perform meta-analysis. The outcomes were presented by type of hormonal contraceptive and by type of study.
	13b	Describe any methods required to prepare the data for presentation or synthesis, such as handling of missing summary statistics, or data conversions.	Page 8-9	The evidence was synthesized according to the type of hormonal contraceptive and outcome quantitatively (association estimator, vote count and meta-analysis) and qualitatively (narrative synthesis) in cases where it was not possible to perform meta-analysis. The outcomes were presented by type of hormonal contraceptive and by type of study.<br>For dichotomous outcomes in which meta-analysis was not performed, crude ORs with their 95% confidence intervals (95%CI) were calculated using Stata 15 statistical software and the Open Epi program, in case of have the information necessary for the calculation. For all outcomes in which meta-analysis could not be
	13c	Describe any methods used to tabulate or visually display results of	Page 25,26	

Section and Topic	Item	Checklist item	Location where item is reported	Brief description
		individual studies and syntheses.		performed, synthesis was performed by vote counting.<br>The quantitative outcomes were synthesized qualitatively. The way the data synthesis was structured is presented in the data synthesis diagram.
	13d	Describe any methods used to synthesize results and provide a rationale for the choice(s). If meta-analysis was performed, describe the model(s), method(s) to identify the presence and extent of statistical heterogeneity, and software package(s) used.	Page 9	Statistical analysis through meta-analysis was performed using Review Manager 5.4.1 software, when there were at least 2 studies per outcome, in dichotomous outcomes, when complete outcome data was available, the same effect measures were used, the participants, the exposure and research question were similar and when not there was considerable heterogeneity. The meta-analysis approach adopted was based on an assessment of the clinical, methodological and statistical diversity of the included studies. In cases in which it was possible to carry out meta-analysis, a random effects meta-analysis was carried out using the DerSimonian and Laird method to allow greater generalization and inference of the results to the populations, additionally, because it was taken into account that there is no single effect of exposure but rather a variety of effects and because it allows heterogeneity between studies to be incorporated.
	13e	Describe any methods used to explore possible causes of heterogeneity among study results (e.g. subgroup analysis, meta-regression).	Page 7-8	A subgroup analysis was performed by type of hormonal contraceptive; it was not possible to perform a subgroup analysis by the degree of hypertension. Subgroup analyzes were also performed by the definition of the exposure of "current use" of hormonal contraceptives, which took into account the duration of previous use of hormonal contraceptives at the time of the index date of the outcome evaluated when heterogeneity was present. considerable.<br>The presence of clinical, methodological and statistical

Section and Topic	Item	Checklist item	Location where item is reported	Brief description
				heterogeneity of the included studies was evaluated.
	13f	Describe any sensitivity analyses conducted to assess robustness of the synthesized results.	Page 9-10	For the sensitivity analysis, in order to evaluate the robustness of the conclusions, the outcomes were compared according to the type of study design, taking into account the evaluation of the risk of bias, the methodological quality or the critical approach as appropriate.
Reporting bias assessment	14	Describe any methods used to assess risk of bias due to missing results in a synthesis (arising from reporting biases).	Page 8	It was considered that if there were 10 or more studies, it would be evaluated using graphical methods (funnel plot) or statistical methods, when the number was less than 10 studies, qualitatively.
Certainty assessment	15	Describe any methods used to assess certainty (or confidence) in the body of evidence for an outcome.	Page 9.	The certainty of the evidence was assessed for the outcome reported in the different types of study designs using the GRADE approach.
**Results**				
Study selection	16a	Describe the results of the search and selection process, from the number of records identified in the search to the number of studies included in the review, ideally using a flow	Page 11	Figure 1

Section and Topic	Item	Checklist item	Location where item is reported	Brief description
		diagram.		
	16b	Cite studies that might appear to meet the inclusion criteria, but which were excluded, and explain why they were excluded.	Appendix H	Appendix H
Study characteristics	17	Cite each included study and present its characteristics.	Page 11	Table 1, Appendices K-L
Risk of bias in studies	18	Present assessments of risk of bias for each included study.	Page 12	Table 2-4, Appendix K
Results of individual studies	19	For all outcomes, present, for each study: (a) summary statistics for each group (where appropriate) and (b) an effect estimate and its precision (e.g. confidence/credible interval), ideally using structured tables or plots.	Page 12-22	Figure 2-5<br>Appendices P-Y
Results of syntheses	20a	For each synthesis, briefly summarise the characteristics and risk of bias	Page 12-22	Appendices M-N

Section and Topic	Item	Checklist item	Location where item is reported	Brief description
		among contributing studies.		
	20b	Present results of all statistical syntheses conducted. If meta-analysis was done, present for each the summary estimate and its precision (e.g. confidence/credible interval) and measures of statistical heterogeneity. If comparing groups, describe the direction of the effect.	Page 13, 14, 21, 22	Figure 2-5
	20c	Present results of all investigations of possible causes of heterogeneity among study results.	Page 13, 21	Figure 2, Figure 4
	20d	Present results of all sensitivity analyses conducted to assess the robustness of the synthesized	Page 12-22	For the sensitivity analysis, in order to evaluate the robustness of the conclusions, the outcomes were compared according to the type of study design, taking into account the evaluation of the risk of bias, the methodological quality or the critical approach as appropriate.

Section and Topic	Item	Checklist item	Location where item is reported	Brief description
		results.		
Reporting biases	21	Present assessments of risk of bias due to missing results (arising from reporting biases) for each synthesis assessed.	Appendices R and Y	It was considered that if there were 10 or more studies, it would be evaluated using graphical methods (funnel plot) or statistical methods, when the number was less than 10 studies, qualitatively. Given that there were fewer than 10 studies for each outcome, we assessed it qualitatively.
Certainty of evidence	22	Present assessments of certainty (or confidence) in the body of evidence for each outcome assessed.	Page 12-22	Each outcome has its evaluation of the certainty of evidence.
**Discussion**				
Discussion	23a	Provide a general interpretation of the results in the context of other evidence.	Page 23-24	The results were compared with other systematic reviews, expert consensus, and a clinical management guideline. The latter is also based on systematic reviews and previous studies
	23b	Discuss any limitations of the evidence included in the review.	Page 25	The outcomes evaluated had very low certainty of evidence and only one had low certainty of evidence and no study found evaluated outcomes of interest for other contraceptive methods such as the patch, levonorgestrel and etonogestrel implants, emergency contraceptives or IUD.
	23c	Discuss any limitations of the review processes used.	Page 25	There was no typing in the studies identified and evaluated. There are also no tools for evaluating bias in case-control studies or case series.

Section and Topic	Item	Checklist item	Location where item is reported	Brief description
	23d	Discuss implications of the results for practice, policy, and future research.	Page 25	The relationship between the use of COC in hypertensive patients and its relationship with hemorrhagic CVD is presented, even with low quality, and it is suggested that RCT or high-quality cohort studies be carried out to better evaluate the safety and effectiveness of COC. in hypertensive.
**Other information**				
Registration and protocol	24a	Provide registration information for the review, including register name and registration number, or state that the review was not registered.	Title page	Systematic review registered in PROSPERO with number CRD42022324806
	24b	Indicate where the review protocol can be accessed, or state that a protocol was not prepared.	Title page	Systematic review registered in PROSPERO with number CRD42022324806
	24c	Describe and explain any amendments to information provided at registration or in the protocol.	Page 10	• We added the category "current use" vs. "non-current use" of hormonal contraceptives as a new comparison based on data from case-control studies.<br>• Secondary outcomes were added: peripheral arterial disease and alteration of metabolic parameters.<br>• A new exposure category called "combined and progestin-only hormonal contraceptives" was created.

Section and Topic	Item	Checklist item	Location where item is reported	Brief description
Support	25	Describe sources of financial or non-financial support for the review, and the role of the funders or sponsors in the review.	Title page	This systematic review did not receive any financial support from any entity for its completion.
Competing interests	26	Declare any competing interests of review authors.	Title page	The authors report no conflicts of interest.
Availability of data, code and other materials	27	Report which of the following are publicly available and where they can be found: template data collection forms; data extracted from included studies; data used for all analyses; analytic code; any other materials used in the review.		Data collection model: Appendix 4; data extracted from included studies: Table 5; Data used for all analyses: Appendix 6 and 7; Analytical code: Appendix 7, 8, 9 and 10.

From (12)
