# Supplementary material for: Safety and effectiveness of hormonal vs non-hormonal or no contraception in women with hypertension and future fertility desire: A broad-scope systematic review
Source: PLoS One. 2026 Mar 31;21(3):e0345959. doi: 10.1371/journal.pone.0345959 (PMC13038026; doi:10.1371/journal.pone.0345959)
Supplement: S5 Appendix — (PDF) [file pone.0345959.s005.pdf]

E. Appendix S5: MOOSE Checklist for Meta-analyses of Observational Studies

Item No.	Recommendation	Reported on page No.	Brief description
Reporting of background should include			
1	Problem definition	Page 6	Hypertensive women need effective and safe contraceptive methods. The information available in the current recommendations regarding this topic is of low quality.
2	Hypothesis statement	Page 7	Hormonal methods are very effective, but could increase cardiovascular risk in hypertensive women.
3	Description of study outcome(s)	Page 17 to 18	The primary outcomes considered were: MACE and unwanted pregnancies. Secondary outcomes included: Pearl index, pelvic inflammatory disease (PID), vaginal infections, loss of fertility, discontinuation of contraception due to side effects or interactions with medications for chronic diseases, worsening of underlying medical condition, peripheral arterial disease, venous thromboembolism, weight gain and alteration in liver or kidney function tests.
4	Type of exposure or intervention used	Page 17 to 18	Studies that included women of reproductive age with high blood pressure exposed to hormonal contraceptive methods were included.
5	Type of study designs used	Page 18	This systematic review considered eligible to evaluate safety: clinical experiments, cohort studies, cases and controls, case reports and series, records of clinical experiments and reports of adverse events from databases specialized in reporting adverse events and post-marketing safety. . To evaluate effectiveness, clinical experiments and cohort studies were considered eligible.
6	Study population	Page 17 to 18	Women of reproductive age with desire for future fertility and high blood pressure exposed to hormonal contraceptive methods

Item No.	Recommendation	Reported on page No.	Brief description
Reporting of search strategy should include			
7	Qualifications of searchers (eg, librarians and investigators)	Page<br><br>1, 16 to 29	Investigator credentials are indicated in the manuscript and in the author contributions.
8	Search strategy, including time period included in the synthesis and key words	Page 16 to 29	An initial search was conducted between September 29 and October 4, 2022, followed by a first update between September 12 and 13, 2023 and a second update between August 7 and 8, 2024, in the databases Medline (via Ovid), Embase, Cochrane Central Registry of Controlled Trials (CENTRAL) and the database of Latin American and Caribbean Literature in Life Sciences Health (LILACS). Additionally, on June 27, 2022, August 26 and 27, 2023, and September 11 and 12, 2024, searches were carried out in the registries of clinical trials, regulatory agencies, and databases specialized in reporting adverse events. , post-marketing safety and gray literature bases. The search strategies are found in Appendix 4.
9	Effort to include all available studies, including contact with authors	Page 24	In the presence of missing data, we sought to contact the authors of the studies to recover the information.
10	Databases and registries searched	Page 18	Table 4.
11	Search software used, name and version, including special features used (eg, explosion)	Page 20	After searching the databases, the records were entered into Rayyan to identify and remove duplicates, as well as screen by title and abstract.

Item No.	Recommendation	Reported on page No.	Brief description
12	Use of hand searching (eg, reference lists of obtained articles)	Page 18	A manual search of the references of the included studies was performed to ensure that all studies of interest were available.
13	List of citations located and those excluded, including justification	Page 31 and 261	Appendix 5
14	Method of addressing articles published in languages other than English	Page 17	There was no restriction on language or publication date.
15	Method of handling abstracts and unpublished studies	Page 16	Abstracts and unpublished studies were not included in the inclusion criteria.
16	Description of any contact with authors	Page 21	In the presence of missing data, we sought to contact the authors of the studies to recover the information.
Reporting of methods should include			
17	Description of relevance or appropriateness of studies assembled for assessing the hypothesis to be tested	Page 4	This systematic review considered eligible to evaluate safety: clinical experiments (RCTs), cohort studies, cases and controls, reports and case series, registries of clinical experiments and reports of adverse events from databases specialized in reporting adverse events. and post-marketing safety. To evaluate effectiveness, clinical experiments and cohort studies were considered eligible.
18	Rationale for the selection and coding of	Page 21	The items on the data extraction forms for each study design are found in Table 5.

Item No.	Recommendation	Reported on page No.	Brief description
	data (eg, sound clinical principles or convenience)		
19	Documentation of how data were classified and coded (eg, multiple raters, blinding and interrater reliability)	Page 31, 268 and 279	Table 6, Appendix 6, Appendix 7
20	Assessment of confounding (eg, comparability of cases and controls in studies where appropriate)	Page 25	For the dichotomous primary and secondary outcomes, we sought to use estimators adjusted for possible confounding variables that would present the relationship between the exposure and the outcome in hypertensive women (the hypertensive population in both the numerator and the denominator). In the absence of these, crude Odds Ratios (OR) were calculated along with their respective confidence intervals as measures of association. It was not possible to calculate ORs adjusted for potential confounding variables, as we did not have the original data from the studies.
21	Assessment of study quality, including blinding of quality assessors, stratification or regression on possible predictors of study results	Page 20	Two reviewers (NL, AB, MH, PG) independently assessed the risk of bias in the cohort studies, the methodological quality of the case-control studies, and the critical approach of the included case series studies. This information was compiled in REDCap. Any disagreements were resolved through dialogue.<br><br>To assess the risk of bias in cohort studies, the ROBINS-I tool was used, for case-control studies, methodological quality was assessed by using the Newcastle-Ottawa tool and case series studies. The critical approach tool of the Joanna Briggs Institute was used. The certainty of the evidence was assessed for the outcome reported in the different types of study designs using the GRADE approach.

Item No.	Recommendation	Reported on page No.	Brief description
22	Assessment of heterogeneity	Page 24	The presence of clinical, methodological and statistical heterogeneity of the included studies was evaluated.
23	Description of statistical methods (eg, complete description of fixed or random effects models, justification of whether the chosen models account for predictors of study results, dose-response models, or cumulative meta-analysis) in sufficient detail to be replicated	Page 25	The meta-analysis approach adopted was based on an assessment of the clinical, methodological and statistical diversity of the included studies. In cases in which it was possible to carry out meta-analysis, a random effects meta-analysis was carried out using the DerSimonian and Laird method to allow greater generalization and inference of the results to the populations, additionally, because it was taken into account that there is no single effect of exposure but rather a variety of effects and because it allows heterogeneity between studies to be incorporated.
24	Provision of appropriate tables and graphics	Page 27, 30, 21, 44	We include in Figure 1 the diagram for the synthesis of the data; in Figure 2 the PRISMA flow chart; in Table 6 the general characteristics of the included studies; Table 7-14 summarizes the results using the vote counting method. Meta-analysis and subgroup analysis in Figures 3-6
Reporting of results should include			
25	Graphic summarizing individual study estimates and overall estimate	Page 73-129	Table 7-14<br>Figure 3-6
26	Table giving descriptive information	Page 268	Appendix 6

Item No.	Recommendation	Reported on page No.	Brief description
	for each study included		
27	Results of sensitivity testing (eg, subgroup analysis)	Page 83, 128	Figure 3, Figure 5
28	Indication of statistical uncertainty of findings		The estimated ORs were presented with their respective 95% confidence intervals. In cases where meta-analysis could be performed, the grouped ORs were presented with their respective 95% confidence intervals, and the I2 and chi2 p values were also presented.
Reporting of discussion should include			
29	Quantitative assessment of bias (eg, publication bias)	Page 132	The risk of publication bias could not be assessed using statistical methods because there were fewer than 10 studies per intervention or outcome, although the use of highly sensitive search methods prevented all studies from being included
30	Justification for exclusion (eg, exclusion of non-English language citations)	Page 261	Appendix 5
31	Assessment of quality of included studies	Page 129 to 130	All outcomes had very low certainty of evidence, except for the relationship between combined oral contraceptives and hemorrhagic cerebrovascular events (low evidence).<br>Appendix 8
Reporting of conclusions should include			
32	Consideration of alternative explanations for observed results	Page 131 and 132	The main limitations include the inability to meet the objective of evaluating the degree of hypertension and the safety of contraceptives, due to non-typing in the studies. All outcomes had very low certainty of evidence, except for the relationship between combined oral contraceptives and hemorrhagic cerebrovascular events (low evidence). There are currently no adequate

Item No.	Recommendation	Reported on page No.	Brief description
			tools available to assess bias in case-control studies or case series. Information on comorbidities, cardiovascular risk factors, duration of contraceptive exposure, and ages of hypertensive women exposed to hormonal contraceptives was not available in the included studies.
33	Generalization of the conclusions (ie, appropriate for the data presented and within the domain of the literature review)	Page 133	Evidence suggests that the use of combined oral contraceptives in hypertensive women may increase the risk of hemorrhagic CVD, but the certainty of the evidence is low. Regarding the other identified outcomes, the certainty of the evidence is very low; Therefore, the effects on the safety and effectiveness of combined oral contraceptives, combined vaginal ring, combined injectable contraceptives, progestin-only pills and injectables in hypertensive women of childbearing age cannot be conclusively established.
34	Guidelines for future research	Page 133	The ideal would be to carry out pragmatic RCTs; if this is not possible, cohort studies are proposed using information from administrative databases, performing causal inference analysis. Current recommendations should continue to be used given the high uncertainty of the results, except in the case of COC in hemorrhagic CVD, where evidence suggests that their use could increase the risk. Regarding the evaluation of safety and effectiveness, studies are required that provide high-quality evidence by controlling the risk of bias, with adequate sample sizes and with detailed population descriptions.
35	Disclosure of funding source	Page 2	This research did not receive any specific grant from funding agencies in the public, commercial, or not-for-profit sectors

From [13]
