# Supplementary material for: Safety and effectiveness of hormonal vs non-hormonal or no contraception in women with hypertension and future fertility desire: A broad-scope systematic review
Source: PLoS One. 2026 Mar 31;21(3):e0345959. doi: 10.1371/journal.pone.0345959 (PMC13038026; doi:10.1371/journal.pone.0345959)
Supplement: S6 Appendix — (PDF) [file pone.0345959.s006.pdf]

F. Appendix S6: Sources of information

Category	Information sources
Databases	● Medline (via Ovid).<br>● Embase.<br>● CENTRAL.<br>● LILACS.
Clinical Trial Records	● ClinicalTrials.gov<br>● Portal of the WHO International Clinical Trials Registration Platform (ICTRP).<br>● Registry of Clinical Experiments of the European Union.
Regulatory agencies, databases specialized in reporting adverse events and post-marketing safety	● FDA Adverse Event Reporting Reporting Program.<br>● EMA European database of reports of suspected adverse reactions.<br>● MedWatch.<br>● Drugs@FDA.<br>● WHO Uppsala Monitoring Center.<br>● Safety warnings, alerts and recalls for drugs or medical devices from the Medicines and Healthcare products Regulatory Agency (MHRA) in the United Kingdom.<br>● Australian Government Department of Health Adverse Event Reporting Database.<br>● Canadian Agency for Drugs and Health Technologies (CADTH).<br>● National Health Surveillance Agency (ANVISA).<br>● Ministry of Health and Social Protection of Colombia with the National Institute for Medicines Surveillance (INVIMA).
Gray literature	● Google scholar<br>● National Technical Information Service (NTIS)
