# Supplementary material for: Safety and effectiveness of hormonal vs non-hormonal or no contraception in women with hypertension and future fertility desire: A broad-scope systematic review
Source: PLoS One. 2026 Mar 31;21(3):e0345959. doi: 10.1371/journal.pone.0345959 (PMC13038026; doi:10.1371/journal.pone.0345959)
Supplement: S7 Appendix — (PDF) [file pone.0345959.s007.pdf]

G. Appendix S7: Search logs

Database: Medline

Characteristic	Report				
Search type	Electronic				
Database	Medline				
Platform	Ovid				
Search date	First search: 04/October/2022<br>First search update: 12/September/2023<br>Second search update: 08/August/2024				
Date range of the search	No restrictions				
Language restrictions	No restrictions				
Other limits	None				
Search strategy	No.	Search strategy	Results of the first search	Results of the first update	Results of the second update
	1	exp hypertension/	310437	345185	352123
	2	exp essential hypertension/	2628	3231	3261
	3	(hypertens$ or prehypertens$).ti,ab.	483080	825520	879302
	4	((elevat$ or increas$ or lower or high or rais$ or rising) adj2 (arterial pressure or blood pressure or diastolic pressure or systolic pressure)).ti,ab.	66854	120541	129162
	5	((elevat$ or increas$ or lower or high or rais$ or rising) adj2 (bp or dbp or hbp or sbp)).ti,ab.	16443	36333	38944
	6	exp Heart Disease Risk Factors/	4945	6490	7984
	7	cardiovascular risk factors.ti,ab.	34941	61284	64553
	8	exp Contraception/	28969	30562	31127
	9	contracep$.ti,ab.	73008	114494	119723
	10	exp Contraceptive Agents/	77979	86645	88330
	11	(contracep$ adj3 agent$).ti,ab.	1180	1539	1583
	12	exp Hormonal Contraception/	152	210	216
	13	(hormonal adj3 contracep$).ti,ab.	6358	11096	11764
	14	exp Contraceptives, Oral, Combined/	5192	6273	6459
	15	(oral adj3 contracep$).ti,ab.	27488	42534	43992
	16	(contraceptive$ adj3 agent$ adj3 female adj3 combined).ti,ab.	0	0	0
	17	exp Transdermal Patch/	1521	1923	2011
	18	(transdermal adj3 patch).ti,ab.	1877	4253	4446

	19	(patch adj5 (transdermal or skin)).ti,ab.	3751	7802	8144
	20	(vaginal adj3 ring).ti,ab.	943	1941	2055
	21	(inject$ adj3 (antifert$ or contracep$)).ti,ab.	1588	2943	3102
	22	exp Progestins/	71231	75581	76850
	23	(contraception and progest$).ti,ab.	3303	4998	5213
	24	exp Levonorgestrel/	4655	5910	6161
	25	levonorgestrel.ti,ab.	5149	9502	9836
	26	(contracep$ and etonogestrel).ti,ab.	513	1002	1080
	27	exp Medroxyprogesterone Acetate/	5047	6252	6374
	28	(contracep$ and medroxyprogeste$).ti,ab.	1621	2577	2703
	29	(contracep$ and (norethindrone or ethynodiol or lynestrenol or norethynodrel)).ti,ab.	1068	1492	1535
	30	(contracep$ and norethisterone).ti,ab.	819	1139	1144
	31	(contracep$ and (ulipristal adj1 acetate)).ti,ab.	198	363	377
	32	exp Intrauterine Devices/	12068	13106	13398
	33	exp Intrauterine Devices, Medicated/	3649	4299	4463
	34	(intrauterine adj3 (device$ or dispositive or levonogestrel or hormone or progest$ or medicat$ or releas$)).ti,ab.	8701	14840	15736
	35	iud.ti,ab.	7489	11077	11538
	36	(contracep$ and ethinylestradiol).ti,ab.	1197	2121	2155
	37	(contracep$ and estradiol).ti,ab.	4204	6659	6832
	38	(contracep$ and gestoge$).ti,ab.	19	34	32
	39	(contracep$ and (norgestimate or gestodene or desogestrel)).ti,ab.	1426	2467	2493
	40	(contracep$ and (drospirenone or cyproterone)).ti,ab.	964	1820	1885
	41	1 or 2 or 3 or 4 or 5 or 6 or 7	614642	1012454	1074177
	42	8 or 9 or 10 or 11 or 12 or 13 or 14 or 15 or 16 or 17 or 18 or 19 or 20 or 21 or 22 or 23 or 24 or 25 or 26 or 27 or 28 or 29 or 30 or 31 or 32 or 33 or 34 or 35 or 36 or 37 or 38 or 39 or 40	199660	256959	264719

	43	41 and 42	3949	5612	5861
References identified	First search: 3949<br>First search update: 5612<br>Second search update: 5861				

Database: Embase

Characteristic	Report				
Search type	Electronic				
Database	Embase				
Platform	Elsevier				
Search date	First search: 29/September/2022<br>First search update: 13/ September /2023<br>Second search update: 07/August/2024				
Date range of the search	No restrictions				
Language restrictions	No restrictions				
Other limits	None				
Search strategy	No.	Search strategy	Results of the first search	Results of the first update	Results of the second update
	#1	'hypertension'/exp	889391	1013915	1013915
	#2	'essential hypertension'/exp	29882	30666	30666
	#3	(hypertens* OR prehypertens*):ab,ti,kw	776406	821650	864635
	#4	((elevat* OR increas* OR lower OR high OR rais* OR rising) NEAR/2 ('arterial pressure' OR 'blood pressure' OR 'diastolic pressure' OR 'systolic pressure')):ab,ti,kw	100004	99375	99375
	#5	((elevat* OR increas* OR lower OR high OR rais* OR rising) NEAR/2 (bp OR dbp OR hbp OR sbp)):ab,ti,kw	27307	29145	29145
	#6	'heart disease risk factor'/exp	209	408	408
	#7	(cardiovascular NEAR/3 risk NEAR/3 factor*):ab,ti,kw	97307	102987	102987
	#8	'contraception'/exp	182767	190068	190068
	#9	(contracep*):ab,ti,kw	93013	96613	96613
	#10	'Contraceptive Agents'/exp	171180	177700	177700
	#11	(contracep* NEAR/3 agent*):ab,ti,kw	1474	1518	1518
	#12	'Hormonal Contraception'/exp	6534	6880	6880
	#13	(hormonal NEAR/3 contracep*):ab,ti,kw	9624	10173	10173

	#14	'Contraceptives, Oral, Combined'/exp	73762	76758	76758
	#15	(oral NEAR/3 contracep*):ab,ti,kw	34907	35847	35847
	#16	(contraceptive* NEAR/3 agent* NEAR/3 female NEAR/3 combined):ab,ti,kw	0	0	0
	#17	'Transdermal Patch'/exp	6114	6641	6641
	#18	(transdermal NEAR/3 patch):ab,ti,kw	3113	3299	3299
	#19	(patch NEAR/5 (transdermal OR skin)):ab,ti,kw	6098	6459	6459
	#20	(vaginal NEAR/3 ring):ab,ti,kw	1588	1648	1648
	#21	(inject* NEAR/3 (antifert* OR contracep*)):ab,ti,kw	1851	1907	1907
	#22	'Progestins'/exp	196402	204567	204567
	#23	(contraception and progest*):ab,ti,kw	4456	4633	4633
	#24	'Levonorgestrel'/exp	12910	14348	14348
	#25	(levonorgestrel):ab,ti,kw	6938	7251	7251
	#26	(contracep* NEAR/3 etonogestrel):ab,ti,kw	274	295	295
	#27	'Medroxyprogesterone Acetate'/exp	18528	19007	19007
	#28	(contracep* NEAR/3 medroxyprogesteron*):ab,ti,kw	418	434	434
	#29	(contracep* and (norethindrone or ethynodiol or lynestrenol or norethynodrel)):ab,ti,kw	968	984	984
	#30	(contracep* and norethisterone):ab,ti,kw	824	836	836
	#31	contracept*:ab,ti,kw AND ((ulipristal NEAR/1 acetate):ab,ti,kw)	350	368	368
	#32	'Intrauterine Devices'/exp	22435	23460	23460
	#33	'Intrauterine Devices, Medicated'/exp	22435	23460	23460
	#34	(intrauterine NEAR/3 (device* OR dispositive OR levonogestrel OR hormone OR progest* OR medicat* OR releas*)):ab,ti,kw	12648	13179	13179
	#35	(iud):ab,ti,kw	8560	8916	8916
	#36	(contracep* NEAR/3 ethinylestradiol):ab,ti,kw	351	356	356
	#37	(contracep* and estradiol):ab,ti,kw	4724	4877	4877

	#38	(contracep* and gestoge$):ab,ti,kw	18	18	18
	#39	(contracep* and (norgestimate or gestodene or desogestrel)):ab,ti,kw	1701	1732	1732
	#40	(contracep* NEAR/3 (drospirenone OR cyproterone)):ab,ti,kw	1518	371	371
	#41	#1 OR #2 OR #3 OR #4 OR #5 OR #6 OR #7	1239749	1370067	1370067
	#42	#8 OR #9 OR #10 OR #11 OR #12 OR #13 OR #14 OR #15 OR #16 OR #17 OR #18 OR #19 OR #20 OR #21 OR #22 OR #23 OR #24 OR #25 OR #26 OR #27 OR #28 OR #29 OR #30 OR #31 OR #32 OR #33 OR #34 OR #35 OR #36 OR #37 OR #38 OR #39 OR #40	473608	491547	491547
	#43	#41 AND #42	16178	18546	19694
References identified	First search: 16178<br>First search update: 18546<br>Second search update: 19694				

Database: Cochrane Controlled Register of Trials (CENTRAL)

**Characteristic**	**Report**				
Search type	Electronic				
Database	CENTRAL				
Platform	Ovid				
Search date	First search: 29/September/2022<br>First search update: 12/September/2023<br>Second search update: 08/August/2024				
Date range of the search	No restrictions				
Language restrictions	No restrictions				
Other limits	None				
Search strategy	No.	Search strategy	Results of the first search	Results of the first update	Results of the second update
	1	exp hypertension/	20361	26500	25800
	2	exp essential hypertension/	231	474	400
	3	(hypertens$ or prehypertens$).ti,ab.	65410	66256	69958
	4	((elevat$ or increas$ or lower or high or rais$ or rising) adj2 (arterial pressure or blood	10519	10791	11571

		pressure or diastolic pressure or systolic pressure)).ti,ab.			
	5	((elevat$ or increas$ or lower or high or rais$ or rising) adj2 (bp or dbp or hbp or sbp)).ti,ab.	3306	3447	3726
	6	exp Heart Disease Risk Factors/	315	512	650
	7	cardiovascular risk factors.ti,ab.	5427	5361	5680
	8	exp Contraception/	591	864	851
	9	contracep$.ti,ab.	15140	16129	17450
	10	exp Contraceptive Agents/	6825	7313	8036
	11	(contracep$ adj3 agent$).ti,ab.	77	79	79
	12	exp Hormonal Contraception/	7	24	7
	13	(hormonal adj3 contracep$).ti,ab.	1833	1879	2047
	14	exp Contraceptives, Oral, Combined/	944	1006	1124
	15	(oral adj3 contracep$).ti,ab.	4955	5019	5239
	16	(contraceptive$ adj3 agent$ adj3 female adj3 combined).ti,ab.	0	0	0
	17	exp Transdermal Patch/	286	359	352
	18	(transdermal adj3 patch).ti,ab.	1260	1296	1370
	19	(patch adj5 (transdermal or skin)).ti,ab.	1718	1764	1870
	20	(vaginal adj3 ring).ti,ab.	550	548	575
	21	(inject$ adj3 (antifert$ or contracep$)).ti,ab.	829	867	924
	22	exp Progestins/	3005	3352	3729
	23	(contraception and progest$).ti,ab.	714	733	792
	24	exp Levonorgestrel/	1010	1098	1253
	25	levonorgestrel.ti,ab.	1930	1982	2074
	26	(contracep$ and etonogestrel).ti,ab.	213	225	239
	27	exp Medroxyprogesterone Acetate/	1067	1151	1227
	28	(contracep$ and medroxyprogeste$).ti,ab.	342	346	368
	29	(contracep$ and (norethindrone or ethynodiol or lynestrenol or norethynodrel)).ti,ab.	216	209	214
	30	(contracep$ and norethisterone).ti,ab.	186	182	185
	31	(contracep$ and (ulipristal adj1 acetate)).ti,ab.	57	57	62
	32	exp Intrauterine Devices/	760	859	1018

	33	exp Intrauterine Devices, Medicated/	494	554	659
	34	(intrauterine adj3 (device$ or dispositive or levonogestrel or hormone or progest$ or medicat$ or releas$)).ti,ab.	2443	2638	2818
	35	iud.ti,ab.	1719	1851	1963
	36	(contracep$ and ethinylestradiol).ti,ab.	560	574	597
	37	(contracep$ and estradiol).ti,ab.	1370	1399	1452
	38	(contracep$ and gestoge$).ti,ab.	6	6	6
	39	(contracep$ and (norgestimate or gestodene or desogestrel)).ti,ab.	637	648	664
	40	(contracep$ and (drospirenone or cyproterone)).ti,ab.	462	475	495
	41	1 or 2 or 3 or 4 or 5 or 6 or 7	79198	81468	85625
	42	8 or 9 or 10 or 11 or 12 or 13 or 14 or 15 or 16 or 17 or 18 or 19 or 20 or 21 or 22 or 23 or 24 or 25 or 26 or 27 or 28 or 29 or 30 or 31 or 32 or 33 or 34 or 35 or 36 or 37 or 38 or 39 or 40	24116	25751	27799
	43	41 and 42	732	771	841
References identified	First search: 732<br>First search update: 771<br>Second search update: 841				

Database: LILACS

**Characteristic**	**Report**				
Search type	Electronic				
Database	LILACS				
Platform	Portal Regional de la Biblioteca Virtual en Salud (BVS)				
Search date	First search: 26/September/2022<br>First search update: 12/September/2023<br>Second search update: 08/August/2024				
Date range of the search	No restrictions				
Language restrictions	No restrictions				
Other limits	None				
Search strategy	No.	Search strategy	Results of the first search	Results of the first update	Results of the second update
	1	Hypertension	22025	23150	24097

	2	Hipertensión	19.690	20548	21231
	3	Hipertensão	18603	19232	19688
	4	“Hormonal Contraception”	432	455	478
	5	Anticoncepción Hormonal	209	220	237
	6	Contracepção Hormonal	173	188	199
	7	Contraceptives, Oral, Combined	140	146	151
	8	Anticonceptivos Orales Combinados	139	142	144
	9	Anticoncepcionais Orais Combinados	132	135	136
	10	Transdermal Patch	54	56	61
	11	Parche Transdérmico	47	48	50
	12	Adesivo Transdérmico	49	50	52
	13	Medroxyprogesterone	222	226	229
	14	Medroxiprogesterona	283	286	290
	15	Contraceptives, Postcoital	122	130	133
	16	Anticonceptivos Poscoito	117	125	128
	17	Anticoncepcionais Pós-Coito	140	149	152
	18	Levonorgestrel	176	189	200
	19	Levanogestrel	93	102	106
	20	Intrauterine Devices, Medicated	29	34	36
	21	Dispositivos Intrauterinos Medicados AND (db:("LILACS"))	32	37	38
	22	(hypertension) AND ((“hormonal contraception”) OR (contraceptives, oral, combined) OR (transdermal patch) OR (medroxyprogesterone) OR (contraceptives, postcoital) OR (levonorgestrel) OR (intrauterine devices, medicated)) AND (db:("LILACS"))	26	26	26
	23	(hipertensión) AND ((anticoncepción hormonal) OR (anticonceptivos orales combinados) OR (parche transdérmico) OR (medroxiprogesterona) OR (anticonceptivos poscoito) OR (levonorgestrel) OR (dispositivos intrauterinos medicados)) AND (db:("LILACS"))	14	14	14
	24	(hipertensão) AND ((contracepção hormonal) OR (anticoncepcionais orais	18	18	18

		combinados) OR (adesivo transdérmico) OR (medroxiprogesterona) OR (anticoncepcionais pós-coito) OR (levanogestrel) OR (dispositivos intrauterinos medicados)) AND (db:("LILACS"))			
References identified	First search: 58<br>First search update: 58<br>Second search update: 58				

Clinical trial records:
Clinicaltrials.gov

Characteristic	Report				
Search type	Electronic				
Database	ClinicalTrials.gov				
Platform	ClinicalTrials.gov				
Search date	First search: 27/June/2022<br>First search update: 27/August/2023<br>Second search update: 11/September/2024				
Date range of the search	No restrictions				
Language restrictions	No restrictions				
Other limits	None				
Search strategy	No.	Search strategy	Results of the first search	Results of the first update	Results of the second update
	1	Condition or disease:Hypertension Intervention/Treatment: Contraception	2	2	2
	2	Condition or disease:Hypertension Intervention/Treatment: contraception AND hormonal	1	1	1
	3	Condition or disease:Hypertension Intervention/Treatment: contraceptives AND oral AND combined	4	4	4
	4	Condition or disease:Hypertension Intervention/Treatment: transdermal AND patch	4	4	5
	5	Condition or disease:Hypertension	0	0	0

		Intervention/Treatment: vaginal AND ring			
	6	Condition or disease:Hypertension Intervention/Treatment: agent AND injectable AND contraceptive	0	0	0
	7	Condition or disease:Hypertension Intervention/Treatment: progestins	7	7	7
	8	Condition or disease:Hypertension Intervention/Treatment: levonorgestrel	0	0	0
	9	Condition or disease:Hypertension Intervention/Treatment: etonogestrel	0	0	0
	10	Condition or disease:Hypertension Intervention/Treatment: norethindrone AND enanthate	0	0	0
	11	Condition or disease:Hypertension Intervention/Treatment: norethisterone AND enanthate	0	0	0
	12	Condition or disease:Hypertension Intervention/Treatment: ulipristal AND acetate	0	0	0
	13	Condition or disease:Hypertension Intervention/Treatment: intrauterine devices, medicated	0	0	0
References identified	First search: 18<br>First search update: 18<br>Second search update: 19				

International Clinical Trials Registry Platform (ICTRP)

Characteristic	Report
Search type	Electronic
Database	OMS
Platform	International Clinical Trials Registry Platform (ICTRP)
Search date	First search: 27/June/2022<br>First search update: 27/August/2023<br>Second search update: 11/September/2024
Date range of the search	No restrictions
Language restrictions	No restrictions

Other limits	None				
Search strategy	No.	Search strategy	Results of the first search	Results of the first update	Results of the second update
	1	hypertension AND (hormonal AND contraception)	0	0	0
	2	hypertension AND contraception	0	0	0
	3	hypertension AND (contraceptives AND oral AND combined)	1	1	1
	4	hypertension AND (transdermal AND patch)	4	4	3
	5	hypertension AND (vaginal AND ring)	0	0	0
	6	hypertension AND (agent AND injectable AND contraceptive)	0	0	0
	7	hypertension AND progestins	0	0	0
	8	hypertension AND (levonorgestrel)	0	0	0
	9	hypertension AND (etonogestrel)	0	0	0
	10	hypertension AND (norethindrone AND enanthate)	0	0	0
	11	hypertension AND (norethisterone AND enanthate)	0	0	0
	12	hypertension AND (ulipristal AND acetate)	0	0	0
	13	hypertension AND (intrauterine AND devices AND medicated)	0	0	0
References identified	First search: 5<br>First search update: 5<br>Second search update: 5				

Clinical trial records from the European Union

Characteristic	Report
Search type	Electronic
Database	EU Clinical Trials Register
Platform	https://www.clinicaltrialsregister.eu/
Search date	Primera búsqueda: 27/June/2022<br>Primera actualización de la búsqueda: 27/August/2023<br>Segunda actualización de la búsqueda: 11/September/2024
Date range of the search	No restrictions
Language restrictions	No restrictions
Other limits	None

Search strategy	No.	Search strategy	Results of the first search	Results of the first update	Results of the second update
	1	hypertension AND (hormonal AND contraception)	126	135	136
	2	hypertension AND contraception	404	423	425
	3	hypertension AND (contraceptives AND oral AND combined)	38	40	40
	4	hypertension AND (transdermal AND patch)	13	13	13
	5	hypertension AND (vaginal AND ring)	11	12	12
	6	hypertension AND (agent AND injectable AND contraceptive)	7	7	7
	7	hypertension AND progestins	0	0	0
	8	hypertension AND (levonorgestrel)	3	3	3
	9	hypertension AND (etonogestrel)	0	0	0
	10	hypertension AND (norethindrone AND enanthate)	0	0	0
	11	hypertension AND (norethisterone AND enanthate)	0	0	0
	12	hypertension AND (ulipristal AND acetate)	0	0	0
	13	hypertension AND (intrauterine AND devices AND medicated)	0	0	0
References identified	First search: 602<br>First search update: 633<br>Second search update: 636				

Regulatory agencies
U.S. Food and Drug Administration (FDA)

**Characteristic**	**Report**
Search type	Electronic
Database	U.S. Food and Drug Administration
Platform	https://www.fda.gov/drugs/drug-safety-and-availability

Search date	First search:27/June/2022<br>First search update: 27/August/2023<br>Second search update: 11/September/2023				
Date range of the search	No restrictions				
Language restrictions	No restrictions				
Other limits	None				
Search strategy	No.	Search strategy	Results of the first search	Results of the first update	Results of the second update
	1	contraceptives AND oral AND combined	0	0	0
	2	transdermal AND patch	0	0	0
	3	vaginal AND ring	0	0	0
	4	agent AND injectable AND contraceptive	0	0	0
	5	progestins	0	0	0
	6	progestational AND drug	0	0	0
	7	levonorgestrel	0	0	0
	8	etonogestrel	0	0	0
	9	medroxyprogesterone	0	0	0
	10	norethindrone AND enanthate	0	0	0
	11	norethisterone AND enanthate	0	0	0
	12	ulipristal AND acetate	0	0	0
	13	intrauterine AND devices AND medicated	0	0	0
References identified	First search:0<br>First search update: 0<br>Second search update: 0				

MedWatch

Characteristic	Report			
Search type	Electronic			
Database	MedWatch			
Platform	https://www.fda.gov/safety/medwatch-fda-safety-information-and-adverse-event-reporting-program			
Search date	First search: 27/June/2022<br>First search update: 27/August/2023<br>Second search update: 11/September/2024			
Date range of the search	No restrictions			
Language restrictions	No restrictions			
Other limits	None			
No.	Search strategy	Results of the first search	Results of the first update	Results of the second update
**Combined hormonal contraceptives**				
**Combined oral contraceptives**				
Ethinylestradiol and norethisterone				
1	Brevicon	0	0	0
2	Modicon	0	0	0
3	Wera	0	0	0
4	Balziva	0	0	0
5	Briellyn	0	0	0
6	Gildagia	0	0	0
7	Philith	0	0	0
8	Zenchent	0	0	0
Ethinylestradiol and norgestimate				
9	Estarylla	0	0	0
10	Previfem	0	0	0

11	Sprintec	0	0	0
Dropirenone and ethinylestradiol				
12	Ocella	0	0	0
13	Yasmin	0	0	0
14	Zarah	0	0	0
15	Yaz	0	0	0
Dropirenone, ethinylestradiol, and levomefolate				
16	Safyral	0	0	0
17	Beyaz	0	0	0
Ethinylestradiol and norgestrel				
18	Cryselle	0	0	0
19	Elinest	0	0	0
20	Ogestrel	0	0	0
Ethinylestradiol and levonorgestrel				
21	Levora	0	0	0
22	Altavera	0	0	0
23	Daysee	0	0	0
24	Lessina	0	0	0
25	Lybrel	0	0	0
26	LoSeasonique	0	0	0
27	Enpresse	0	0	0
28	Trivora	0	0	0
29	Introvale	0	0	0
30	Seasonique	0	0	0
Estetrol/drospirenone				
31	Nextstellis	0	0	0
Ethinylestradiol / clormadinone				
32	Ethinyl estradiol/chlormadinone	0	0	0
Ethinylestradiol / cyproterone				
33	Ethinyl estradiol/cyproterone	0	0	0
Extended cycle of ethinylestradiol and levonorgestrel				
34	Amethia Lo	0	0	0

35	Camrese Lo	0	0	0
36	Daysee	0	0	0
37	Amethia	0	0	0
38	Ashlyna	0	0	0
39	Jolessa	0	0	0
40	Quasense	0	0	0
41	Cuarteto	0	0	0
42	Amethia	0	0	0
43	Jolessa	0	0	0
Ethinylestradiol and desogestrel				
44	Azurette	0	0	0
45	Kariva	0	0	0
46	Mircette	0	0	0
47	Viorele	0	0	0
48	Caziant	0	0	0
49	Cyclessa	0	0	0
50	Velivet	0	0	0
51	Apri	4	4	4
52	Desogen	0	0	0
53	Juleber	0	0	0
54	Reclipsen	0	0	0
55	Solia	0	0	0
Ethinylestradiol and norethindrone				
56	Aranelle	0	0	0
57	Tri-Norinyl	0	0	0
58	Leena	0	0	0
59	Alyacen 7/7/7	0	0	0
60	Necon 7/7/7	0	0	0
61	Notrel 7/7/7	0	0	0
62	Dasetta 7/7/7	0	0	0
63	Cyclafem 7/7/7	0	0	0
Ethinylestradiol and norgestimate				
64	TriNessa	0	0	0
Dienogest y estradiol				

65	Natazia	0	0	0
**Combined contraceptive patch**				
Norelgestromin/ethinylestradiol				
66	Xulane	0	0	0
67	Ortho Evra	0	0	0
68	Evra	0	0	0
**Combined contraceptive vaginal ring**				
**Etonogestrel/ethinylestradiol**				
69	NuvaRing	0	0	0
70	EluRyng	0	0	0
**Combined injectable contraceptives**				
71	Cyclofem	0	0	0
72	Mesigyna	0	0	0
**Progestin-only contraceptives**				
**Progestin-only pill (or mini-pill)**				
Norethindrone				
73	Aygestin	0	0	0
74	Camila	0	0	0
75	Errin	0	0	0
76	Jolivette	0	0	0
77	Lyza	0	0	0
78	Nora-Be	0	0	0
79	Nor-QD	0	0	0
80	Ortho Micronor	0	0	0
Desogestrel				
81	Desogestrel	0	0	0
**Levonorgestrel and etonogestrel implants**				
Levonorgestrel and etonogestrel				
82	Norplant	0	0	0
83	Jadelle	0	0	0
84	Sino-implant	0	0	0
85	Implanon	0	0	0
86	Nexplanon	0	0	0
**Depot medroxyprogesterone acetate injection**				
Medroxyprogesterone acetate				

87	Medroxyprogesterone acetate	0	0	0
**Norethisterone enanthate/estradiol valerate injectable**				
Norethisterone enanthate/ Estradiol valerate				
88	Norethisterone enanthate	0	0	0
89	Estradiol valerate	0	0	0
**Emergency contraceptives**				
90	Levonorgestrel	0	0	0
91	Ulipristal acetate	0	0	0
**Levonorgestrel-releasing intrauterine device**				
92	Mirena	0	0	0
93	Skyla	0	0	0
94	Liletta	0	0	0
95	Kyleena	0	0	0
96	Jaydess	0	0	0
References identified	First search: 4<br>First search update: 4<br>Second search update: 4			

Drugs@FDA

**Characteristic**	**Report**
Search type	Electronic
Database	Drugs@FDA
Platform	https://www.accessdata.fda.gov/scripts/cder/daf/index.cfm
Database	First search: 27/June/2022<br>First search update: 27/August/2023<br>Second search update: 11/September/2024
Date range of the search	No restrictions

Language restrictions	No restrictions			
Other limits	None			
No.	Search strategy	Results of the first search	Results of the first update	Results of the second update
Combined hormonal contraceptives				
Combined oral contraceptives				
Ethinylestradiol and norethisterone				
1	Brevicon	2	2	2
2	Modicon	2	2	2
3	Wera	1	1	1
4	Balziva	2	2	2
5	Briellyn	1	1	1
6	Gildagia	1	1	1
7	Philith	1	1	1
8	Zenchent	0	0	0
Ethinylestradiol and norgestimate				
9	Estarylla	3	3	3
10	Previfem	2	2	2
11	Sprintec	3	3	3
Dropirenone and ethinylestradiol				
12	Ocella	0	0	0
13	Yasmin	1	1	1
14	Zarah	0	0	0
15	Yaz	3	3	3
Dropirenone, ethinylestradiol, and levomefolate				
16	Safyral	1	1	1
17	Beyaz	1	1	1
Ethinylestradiol and norgestrel				
18	Cryselle	2	2	2
19	Elinest	1	1	1
20	Ogestrel	31	31	31
Ethinylestradiol and levonorgestrel				
21	Levora	2	2	2
22	Altavera	1	1	1
23	Daysee	1	1	1

24	Lessina	2	2	2
25	Lybrel	1	1	1
26	LoSeasonique	1	1	1
27	Enpresse	2	2	2
28	Trivora	2	2	2
29	Introvale	1	1	1
30	Seasonique	2	2	2
Estetrol/drospirenone				
31	Nextstellis	1	1	1
Ethinylestradiol / clormadinone				
32	Ethinyl estradiol/chlormadinone	0	0	0
Ethinylestradiol / cyproterone				
33	Ethinyl estradiol/cyproterone	0	0	0
Extended cycle of ethinylestradiol and levonorgestrel				
34	Amethia Lo	0	0	0
35	Camrese Lo	0	0	0
36	Daysee	1	1	1
37	Amethia	0	0	0
38	Ashlyna	1	1	1
39	Jolessa	0	0	0
40	Quasense	1	1	1
41	Cuarteto	1	1	0
42	Amethia	0	0	0
43	Jolessa	0	0	0
Ethinylestradiol and desogestrel				
44	Azurette	0	0	0
45	Kariva	1	1	1
46	Mircette	1	1	1
47	Viorele	1	1	1
48	Caziant	0	0	0
49	Cyclessa	1	1	1

50	Velivet	3	3	3
51	Apri	30	30	30
52	Desogen	2	2	2
53	Juleber	0	0	0
54	Reclipsen	0	0	0
55	Solia	0	0	0
Ethinylestradiol and norethindrone				
56	Aranelle	1	1	1
57	Tri-Norinyl	2	2	2
58	Leena	1	1	1
59	Alyacen 7/7/7	3	3	3
60	Necon 7/7/7	0	0	0
61	Notrel 7/7/7	0	0	0
62	Dasetta 7/7/7	1	1	1
63	Cyclafem 7/7/7	1	1	1
Ethinylestradiol and norgestimate				
64	TriNessa	0	0	0
Dienogest and estradiol				
65	Natazia	1	1	1
Combined contraceptive patch				
Norelgestromin/ethinylestradiol				
66	Xulane	1	1	1
67	Ortho Evra	1	1	1
68	Evra	1	1	1
Combined contraceptive vaginal ring				
Etonogestrel/ethinylestradiol				
69	NuvaRing	1	1	1
70	EluRyng	1	1	1
Combined injectable contraceptives				
71	Cyclofem	0	0	0
72	Mesigyna	0	0	0
Progestin-only contraceptives				
Progestin-only pill (or mini-pill)				
Norethindrone				

73	Aygestin	1	1	1
74	Camila	1	1	1
75	Errin	2	2	2
76	Jolivette	0	0	0
77	Lyza	2	2	2
78	Nora-Be	0	0	0
79	Nor-QD	1	1	1
80	Ortho Micronor	0	0	0
Desogestrel				
81	Desogestrel	19	19	19
Levonorgestrel and etonogestrel implants				
Levonorgestrel y etonogestrel				
82	Norplant	2	2	2
83	Jadelle	1	1	1
84	Sino-implant	0	0	0
85	Implanon	2	2	2
86	Nexplanon	2	2	2
Depot medroxyprogesterone acetate injection				
Medroxyprogesterone acetate				
87	Medroxyprogesterone acetate	13	13	13
Norethisterone enanthate/estradiol valerate injectable				
Norethisterone enanthate/ Estradiol valerate				
88	Norethisterone enanthate	0	0	0
89	Estradiol valerate	7	7	7
Emergency contraceptives				
90	Levonorgestrel	72	72	73
91	Ulipristal acetate	2	2	2
Levonorgestrel-releasing intrauterine device				
92	Mirena	1	1	1

93	Skyla	1	1	1
94	Liletta	1	1	1
95	Kyleena	1	1	1
96	Jaydess	0	0	0
References identified	First search: 126<br>First search update: 126<br>Second search update: 126			

European Medicines Agency (EMA)

Characteristic	Report				
Search type	Electronic				
Database	European Medicines Agency				
Platform	https://www.ema.europa.eu/en/medicines?search_api_views_fulltext=				
Search date	First search:27/June/2022<br>Primera actualización: 27/August/2023<br>Segunda actualización: 12/September/2024				
Date range of the search	No restrictions				
Language restrictions	No restrictions				
Other limits	None				
Search strategy	No.	Search strategy	Results of the first search	Results of the first update	Results of the second update
	1	contraceptives AND oral AND combined	1022	1149	5
	2	transdermal AND patch	89	100	12
	3	vaginal AND ring	234	254	3
	4	agent AND injectable AND contraceptive	840	948	0
	5	progestins	53	60	0
	6	progestational AND drug	282	309	0
	7	levonorgestrel	172	186	27
	8	etonogestrel	26	27	9
	9	medroxyprogesterone	32	36	1
	10	norethindrone AND enanthate	0	0	0
	11	norethisterone AND enanthate	0	0	0
	12	ulipristal AND acetate	11	12	12
	13	intrauterine AND devices AND medicated	157	179	0
References identified	First search:2918<br>First search update: 3260<br>Second search update: 69				

Uppsala Monitoring Centre

<table>
<tr><th>Characteristic</th><th colspan="5">Report</th></tr>
<tr><td>Search type</td><td colspan="5">Electronic</td></tr>
<tr><td>Database</td><td colspan="5">Uppsala Monitoring Centre</td></tr>
<tr><td>Platform</td><td colspan="5">who-umc.org</td></tr>
<tr><td>Search date</td><td colspan="5">First search:27/June/2022
First search update: 27/August/2023
Second search update: 12/September/2024</td></tr>
<tr><td>Date range of the search</td><td colspan="5">No restrictions</td></tr>
<tr><td>Language restrictions</td><td colspan="5">No restrictions</td></tr>
<tr><td>Other limits</td><td colspan="5">None</td></tr>
<tr><td rowspan="10">Search strategy</td><td>No.</td><td>Search strategy</td><td>Results of the first search</td><td>Results of the first update</td><td>Results of the second update</td></tr>
<tr><td>1</td><td>contraceptives, oral, combined</td><td>0</td><td>0</td><td>325</td></tr>
<tr><td>2</td><td>transdermal patch</td><td>1</td><td>1</td><td>43</td></tr>
<tr><td>3</td><td>vaginal ring</td><td>0</td><td>0</td><td>74</td></tr>
<tr><td>4</td><td>agent injectable contraceptive</td><td>0</td><td>0</td><td>80</td></tr>
<tr><td>5</td><td>progestins</td><td>0</td><td>0</td><td>0</td></tr>
<tr><td>6</td><td>progestational drug</td><td>0</td><td>0</td><td>412</td></tr>
<tr><td>7</td><td>levonorgestrel</td><td>6</td><td>6</td><td>6</td></tr>
<tr><td>8</td><td>etonogestrel</td><td>1</td><td>1</td><td>2</td></tr>
<tr><td>9</td><td>medroxyprogesterone</td><td>5</td><td>5</td><td>5</td></tr>
<tr><td rowspan="4"></td><td>10</td><td>norethindrone enanthate</td><td>0</td><td>0</td><td>1</td></tr>
<tr><td>11</td><td>norethisterone enanthate</td><td>0</td><td>0</td><td>3</td></tr>
<tr><td>12</td><td>ulipristal acetate</td><td>0</td><td>0</td><td>42</td></tr>
<tr><td>13</td><td>intrauterine devices, medicated</td><td>0</td><td>0</td><td>138</td></tr>
<tr><td>References identified</td><td colspan="5">First search: 13
First search update: 13
Second search update: 941</td></tr>
</table>

Medicines and Healthcare Products Regulatory Agency (MHRA)

Characteristic	Report
Search type	Electronic
Database	Medicines and Healthcare Products Regulatory Agency (MHRA)
Platform	https://www.gov.uk/drug-device-alerts
Search date	First search: 27/June/2022<br>First search update: 27/August/2023<br>Second search update: 12/September/2024

Date range of the search	No restrictions				
Language restrictions	No restrictions				
Other limits	None				
Search strategy	No.	Search strategy	Results of the first search	Results of the first update	Results of the second update
	1	contraceptives AND (oral AND combined)	111	113	123
	2	transdermal AND patch	23	24	25
	3	vaginal AND ring	18	18	19
	4	agent AND injectable AND contraceptive	190	220	231
	5	progestins	0	0	0
	6	progestational AND drug	268	281	291
	7	levonorgestrel	0	0	0
	8	etonogestrel	1	1	1
	9	medroxyprogesterone	2	2	2
	10	norethindrone AND enanthate	0	0	0
	11	norethisterone AND enanthate	0	1	2
	12	ulipristal AND acetate	9	9	11
	13	intrauterine AND devices AND medicated	885	1017	1108
References identified	First search: 1507<br>First search update: 1686<br>Second search update: 1813				

Australian Government Department of Health Adverse Event Reporting Database

Characteristic	Report				
Search type	Electronic				
Database	Australian Government Department of Health Adverse Event Reporting Database				
Platform	https://www.tga.gov.au/				
Search date	First search: 27/June/2022<br>First search update: 27/August/2023<br>Second search update: 12/September/2024				
Date range of the search	No restrictions				
Language restrictions	No restrictions				
Other limits	None				
Search strategy	No.	Search strategy	Results of the first search	Results of the first update	Results of the second update

	1	contraceptives AND oral AND combined	26	27	27
	2	transdermal AND Patch	176	191	226
	3	vaginal AND ring	1	1	1
	4	agent AND injectable AND contraceptive	1	7	8
	5	progestins	4	5	5
	6	progestational AND drug	1	1	1
	7	levonorgestrel	78	81	95
	8	etonogestrel	8	15	18
	9	medroxyprogesterone	32	42	41
	10	norethindrone AND enanthate	0	0	0
	11	norethisterone AND enanthate	0	0	0
	12	ulipristal AND acetate	7	8	7
	13	intrauterine AND devices AND medicated	9	15	26
References identified	First search: 343<br>First search update: 393<br>Second search update: 455				

Canadian Agency for Drugs and Technologies in Health (CADTH)

**Characteristic**	**Report**				
Search type	Electronic				
Database	Canadian Agency for Drugs and Technologies in Health (CADTH)				
Platform	https://www.cadth.ca/				
Search date	First search: 27/June/2022<br>First search update: 27/August/2023<br>Second search update: 12/September/2024				
Date range of the search	No restrictions				
Language restrictions	No restrictions				
Other limits	None				
Search strategy	No.	Search strategy	Results of the first search	Results of the first update	Results of the second update
	1	contraceptives AND oral AND combined	98	99	83
	2	transdermal AND patch	41	40	20
	3	vaginal AND ring	5	5	4
	4	agent AND injectable AND contraceptive	56	57	52
	5	progestins	11	11	9
	6	progestational AND drug	0	0	0

	7	levonorgestrel	12	12	4
	8	etonogestrel	5	5	3
	9	medroxyprogesterone	11	11	11
	10	norethindrone AND enanthate	0	0	0
	11	norethisterone AND enanthate	0	0	0
	12	ulipristal AND acetate	7	7	0
	13	intrauterine AND devices AND medicated	30	30	10
References identified	First search: 276<br>First search update: 277<br>Second search update: 196				

National Health Surveillance Agency (ANVISA)

Characteristic	Report				
Search type	Electronic				
Database	Agencia Nacional de Vigilancia Sanitaria (ANVISA)				
Platform	https://www.gov.br/anvisa/pt-br				
Search date	First search: 27/June/2022<br>First search update: 27/August/2023<br>Second search update: 12/September/2024				
Date range of the search	No restrictions				
Language restrictions	No restrictions				
Other limits	None				
Search strategy	No.	Search strategy	Results of the first search	Results of the first update	Results of the second update
	1	contraceptivos E orais E combinados	41	45	47
	2	transdérmico E patch	10	10	12
	3	vaginal E anel	34	48	49
	4	agente E injetável E contraceptivo	26	26	31
	5	progestinas	3	3	3
	6	progestacional E medicamento	2	12	12
	7	levonorgestrel	137	203	283
	8	etonogestrel	93	152	232
	9	medroxiprogesterona	113	176	255
	10	noretindrona E enantato	1	2	1
	11	noretisterona E enantato	103	160	241
	12	ulipristal E acetato	88	142	219
	13	intra-uterino E dispositivos E medicado	55	55	65

References identified	First search: 706<br>First search update: 1034<br>Second search update: 1449

Instituto Nacional de Vigilancia de Medicamentos (INVIMA)

Characteristic	Report				
Search type	Electronic				
Database	Instituto Nacional de Vigilancia de Medicamentos y Alimentos (INVIMA)				
Platform	https://www.invima.gov.co/consulta-registros-sanitarios				
Search date	First search: 27/June/2022<br>First search update: 27/August/2023<br>Second search update: 12/September/2024				
Date range of the search	No restrictions				
Language restrictions	No restrictions				
Other limits	None				
	No.	Search strategy	Results of the first search	Results of the first update	Results of the second update
	1	anticonceptivos orales combinados	0	0	0
	2	parche transdérmico	9	0	0
	3	anillo vaginal	5	5	5
	4	anticonceptivos inyectables	0	0	0
	5	progestágenos	0	0	0
	6	medicamento progestacional	0	0	0
	7	levonorgestrel	6	6	6
	8	etonogestrel	0	0	0
	9	medroxiprogesterona	6	6	6
	10	enantato de noretindrona	0	0	0
	11	enantato de noretisterona	0	0	0
	12	acetato de ulipristal	1	1	1
	13	dispositivo intrauterino medicado	0	0	0
References identified	First search: 27<br>First search update: 18<br>Second search update: 18				

Grey literature
Google scholar

Characteristic	Report
Search type	Electronic
Database	Google scholar
Platform	Google scholar

Search date	First search: 27/June/2023<br>First search update: 26/August/2023<br>Second search update: 12/September/2024				
Date range of the search	No restrictions				
Language restrictions	No restrictions				
Other limits	None				
Search strategy	No.	Search strategy	Results of the first search	Results of the first update	Results of the second update
	1	Hormonal contraception AND hypertension	40	40	40
References identified	First search and updates: 40 first results				

National Technical Information Service (NTIS)

**Characteristic**	**Report**				
Search type	Electronic				
Database	National Technical Information Service (NTIS)				
Platform	National Technical Information Service (NTIS)				
Search date	First search: 27/06/2022<br>First search update: 26/08/2023<br>Second search update: 12/09/2024				
Date range of the search	No restrictions				
Language restrictions	No restrictions				
Other limits	None				
Search strategy	No.	Search strategy	Results of the first search	Results of the first update	Results of the second update
	1	Hormonal contraception AND hypertension	0	0	0
References identified	First search: 0<br>First search update: 0<br>Second search update: 0				
