# Supplementary material for: Safety and effectiveness of hormonal vs non-hormonal or no contraception in women with hypertension and future fertility desire: A broad-scope systematic review
Source: PLoS One. 2026 Mar 31;21(3):e0345959. doi: 10.1371/journal.pone.0345959 (PMC13038026; doi:10.1371/journal.pone.0345959)
Supplement: S8 Appendix — (PDF) [file pone.0345959.s008.pdf]

H. Appendix S8: Characteristics of studies not included

Study	Reason for exclusion	Standardized reason for exclusion
Adler 2018 [31]	Not the exposure/intervention of interest: The intervention in this study is not taken as a contraceptive method, only ethinyl estradiol (without being combined, as found in contraceptives) is administered to women daily in order to see changes in endothelial function.	Does not have the exposure/intervention of interest
Ananijevic-Pandey 1989 [32]	It does not present the population of interest with the exposure to be evaluated.	Wrong population
Andrews 2012 [33]	Analysis of a published study.	Analysis of a published study.
Correia 2016 [34]	Conference summary	Conference summary
Dinger 2010 [35]	Conference summary	Conference summary
Alcalde 2018 [36]	They do not use the contraceptive method for birth control, but rather to control the underlying pathology.	Does not have the exposure/intervention of interest
Dong 1997 [37]	Wrong population. They do not discriminate the results.	Wrong population
Dunn 2001 [38]	Wrong population.	Wrong population
Fish 1977 [39]	Wrong population	Wrong population
Mekonnen 2015 [40]	They do not evaluate the safety or effectiveness of contraceptive methods in hypertensive women.	PICO question does not apply
Millet 1979 [41]	Not available in full text.	Not available in full text.
Kalenga 2019 [42]	Congress summary	Conference summary
Kalenga 2022 [43]	Wrong study.	Wrong study.
Kovell 2022 [44]	They report the percentage of use of contraceptive methods among hypertensive women. They do not evaluate the safety or effectiveness of contraceptive methods in hypertensive women.	PICO question does not apply
Leaf 1991 [45]	Wrong population.	Wrong population.
Lidegaard 1998 [46]	Wrong population.	Wrong population.
Lidegaard 2011 [47]	They do not evaluate the interaction between high blood	Wrong population.

Study	Reason for exclusion	Standardized reason for exclusion
	pressure and the use of contraceptives.	
Lubianca 2003 [69]	It is a cross-sectional study. It is not part of the designs included in this systematic review.	Wrong study.
Dinger 2010 [30]	They do not evaluate the interaction between high blood pressure and the use of contraceptives	Wrong population.
Mann 1975 [48]	In full text	Not available in full text.
Meinel 1988 [49]	In full text	Not available in full text.
Narkiewicz 1995 [68]	It is a cross-sectional study. It is not part of the designs included in this systematic review.	Wrong study.
Nessa 2006 [50]	In full text	Not available in full text.
Perritt 2011 [51]	Wrong population	Wrong population
Wieder 2010 [52]	Congress summary	Conference summary
Arthes 1976 [53]	Wrong population	Wrong population
Chaudhury 1988 [54]	Wrong population	Wrong population
Chen 1999 [55]	Wrong population	Wrong population
Maino 2016 [56]	Wrong population	Wrong population
Sivin 1994 [57]	Wrong population	Wrong population
Ueda 2019 [58]	Wrong population	Wrong population
Wang 2011 [59]	Wrong population	Wrong population
Mant 1998 [60]	Wrong population	Wrong population
Jensen 1991 [61]	Wrong population	Wrong population
Dunn 1999 [62]	They do not describe how many of the hypertensive patients were exposed and how many were not exposed. They only say the hypertensive cases and controls, but they do not say how many were exposed to hormonal contraceptives and how many were not.	Wrong population
D'Avanzo 1994 [63]	Presents measures of association of acute myocardial infarction in exposed hypertensive women compared to unexposed non-hypertensive women. It does not compare exposed hypertensive women with unexposed	Wrong population

Study	Reason for exclusion	Standardized reason for exclusion
	hypertensive women. It is not the white population.	
Lewis 1997 [29]	They do not evaluate the interaction between high blood pressure and the use of contraceptives	Wrong population
Lidegaard 1996 [64]	Wrong study	Wrong study.
Lidegaard 2002 [65]	Does not present data on exposed and unexposed hypertensive patients (cases and controls): The population of interest is not present.	Wrong population
Siritho 2003 [66]	Does not present data on exposed and unexposed hypertensive patients (cases and controls): The population of interest is not present.	Wrong population
Nightingale 2004 [67]	Does not present data on exposed and unexposed hypertensive patients (cases and controls): The population of interest is not present.	Wrong population
Ojelabi 2024 [70]	Poster	Poster
Costescu 2024 [71]	Poster	Poster
Spielvogel 2023 [72]	PICO question does not apply	PICO question does not apply
Neill 2023 [73]	Poster	Poster
Jeong 2023 [74]	They do not describe how many of the hypertensive patients were exposed and how many were not exposed.	Wrong population
Abdrebi 2024 [75]	Poster	Poster
