# Supplementary material for: Safety and effectiveness of hormonal vs non-hormonal or no contraception in women with hypertension and future fertility desire: A broad-scope systematic review
Source: PLoS One. 2026 Mar 31;21(3):e0345959. doi: 10.1371/journal.pone.0345959 (PMC13038026; doi:10.1371/journal.pone.0345959)
Supplement: S9 Appendix — (PDF) [file pone.0345959.s009.pdf]

I. Appendix S9: Data extraction format for studies on hormonal contraceptives and hypertensive women

Category	Extracted data
Study methods (all designs)	Authors, year, study design, time of follow-up or information collection, country or countries where the study was carried out, place where the study was carried out, language, objective of the study, type of sampling and data collection method.
Population (RCTs, cohorts, series and case reports)	Sample size, number of women per exposure/intervention group and comparator, sociodemographic data (age: mean and standard deviation or median and interquartile range), inclusion and exclusion criteria, definition of arterial hypertension in the study, proportion of women with hypertension, presence of comorbidities in the hypertensive women in the study and use of other medications in addition to hormonal contraceptives.
Exposure/Intervention (RCTs, cohorts, series and case reports)	Type of contraceptive method, frequency of use of contraceptive methods, and concentration and dosage of contraceptives used.
Comparator (RCTs and cohorts)	Comparator type.
Population (Case-control studies)	Sample size, number of cases and controls, matching variables, sociodemographic data (age: mean and standard deviation or median and interquartile range), inclusion and exclusion criteria, definition of hypertension in the study, proportion of women with hypertension arterial, presence of comorbidities in the hypertensive women in the study and use of other medications in addition to hormonal contraceptives.
Factor of interest (Case and control studies)	Type of contraceptive method used by the cases and controls, concentration, dose and frequency of use of the contraceptive methods used.
Outcomes (All studies)	The outcomes were reported according to the exposure and non-exposure groups to hormonal contraceptives, according to the type of study. In the case-control studies, the outcomes were represented by the cases.
Financing and conflicts of interest	Funding sources and conflicts of interest.
