# Supplementary figures and images for: Safety and effectiveness of hormonal vs non-hormonal or no contraception in women with hypertension and future fertility desire: A broad-scope systematic review
Source: PLoS One. 2026 Mar 31;21(3):e0345959. doi: 10.1371/journal.pone.0345959 (PMC13038026; doi:10.1371/journal.pone.0345959)

## J. Appendix S10: Diagram for data synthesis

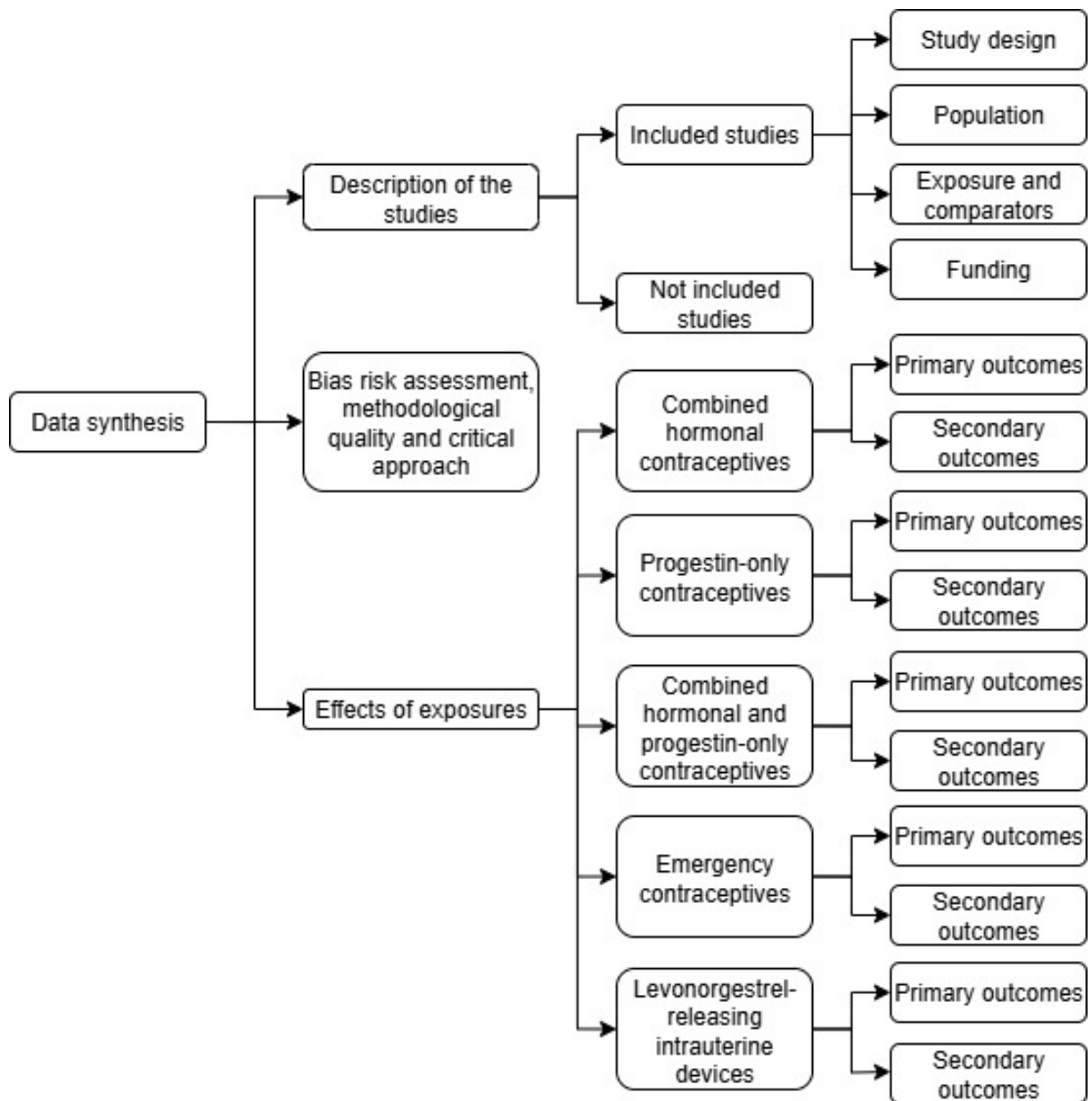

Source: Own elaboration

Supplement: S10 Appendix — (PDF) [file pone.0345959.s010.pdf]
