# Supplementary material for: Safety and effectiveness of hormonal vs non-hormonal or no contraception in women with hypertension and future fertility desire: A broad-scope systematic review
Source: PLoS One. 2026 Mar 31;21(3):e0345959. doi: 10.1371/journal.pone.0345959 (PMC13038026; doi:10.1371/journal.pone.0345959)
Supplement: S11 Appendix — (PDF) [file pone.0345959.s011.pdf]

K. Appendix S11: Detailed characteristics of the included studies and assessment of risk of bias, methodological quality and critical approach

Case-control studies:

Collaborative Group for the Study of Stroke in Young Women 1975 [84]

Feature	Description
Methods	*Study design*: cases and controls.<br>*Data collection dates*: September 1, 1969 to August 31, 1971.<br>*Country*: United States (12 cities).<br>*Research centers*: 91 hospitals.<br>*Language*: English.<br>*Generalities of the study*: The objective was to evaluate the association between the use of oral contraceptives and the presence of cerebrovascular events, both ischemic and hemorrhagic, taking into account cardiovascular risk factors. Sampling was by convenience. The researchers conducted interviews with the women included in the study to identify each woman's exposures.
Population	*Sample size*: 1216.<br>*Groups*: cases: women with ischemic or hemorrhagic cerebrovascular events (n=336), controls: hospital (n=429) and community (n=451).<br>*Pairing variables*: age and race.<br>*Sociodemographic data*: cases and controls: The women who were part of this study were between 15 and 44 years old. They do not present measures of central tendency or age dispersion.<br>*Inclusion criteria*: non-pregnant women from 15 to 44 years old. Cases had to be women with a cerebrovascular event (ischemic or hemorrhagic). Controls had to not be pregnant, within 30 days of giving birth, and had to reside in the same geographic area as the cases. Hospital controls had to have been admitted to the hospital for at least 24 hours and discharged within 2 months of case admission.<br>*Exclusion criteria*: does not report.<br>*Definition of high blood pressure*: for hospital cases and controls: according to the highest blood pressure value in hospital records. In community controls: by interview they identified the use of antihypertensives. Classification: normal blood pressure < 140/90 mmHg, borderline arterial hypertension: 140-159/ 90-94 mmHg, moderate arterial hypertension: 160-179/95-109 mmHg and severe arterial hypertension >=180/110 mmHg.<br>*Hypertensive women*: 399 (32.81%) women were hypertensive, 92 women with ischemic cerebrovascular event were hypertensive, 155 women with hemorrhagic cerebrovascular event were hypertensive and 152 controls were hypertensive.<br>*Comorbidities of hypertensive women*: They do not present the comorbidities that hypertensive women presented.

	Use of other medications in addition to contraceptive methods in hypertensive women: cannot be determined from the study data.
Exposure/Comparator	Exposure was current use of combined oral contraceptives.<br>The comparator was no current use of combined oral contraceptives. They do not present the definition of current use and non-current use of oral contraceptives in relation to time, taking into account the presence of the cerebrovascular event.<br>*Type of hormonal contraceptive*: combined oral contraceptives.<br>They do not specify the classes of combined oral contraceptives used, concentrations, doses or duration of use of the contraceptives.
Outcomes	Ischemic or hemorrhagic cerebrovascular events.<br>For ischemic and hemorrhagic CVD:<br>Cases exposed with HTA: 73<br>Unexposed cases with HTA: 174<br>Controls exposed with HTA: 23<br>Controls not exposed with HTA: 129<br><br>For ischemic CVD:<br>Cases exposed with HTA: 38<br>Unexposed cases with HTA: 54<br>Controls exposed with HTA: 23<br>Controls not exposed with HTA: 129<br><br>For hemorrhagic CVD:<br>Cases exposed with HTA: 35<br>Unexposed cases with HTA: 120<br>Controls exposed with HTA: 23<br>Controls not exposed with HTA: 129<br><br>Association measures (reference: non-hypertensive and non-current contraceptive users):<br><br>ischemic CVD<br>Borderline pressure: RR=5.2 (95% CI 2.3-12).<br>Moderate pressure: RR=8.9 (95% CI 3.5-22.8).<br>High blood pressure: RR=13.6 (95% CI 4.8-38.6).<br><br>Hemorrhagic CVD<br>Borderline pressure: RR=2.8 (95% CI 1-7.9)<br>Moderate pressure: RR =8.4 (95% CI 3-23.1)<br>Severe pressure: RR=25.7 (95% CI 9.4-70.7)<br><br>*Definition of the outcome*: The diagnosis was clinical and imaging, determined by neurologists.
Financing	National Institute of Child Health and Human Development.
Declaration of interests	They don't report.
Notes	No specific definition of “current oral contraceptive use” is provided. The study does not offer details about the comorbidities that hypertensive women could present.

	In this study they describe that they could not establish an association between estrogen doses and the presence of cerebrovascular events, given that 23 of the 25 women with ischemic cerebrovascular events who were taking contraceptives with mestranol, the dose was 100 µg, and 20 who were taking contraceptives with estradiol (in non-sequential pills) used doses of 50µg.
**Methodological quality: Newcastle Ottawa case-control**	
Good methodological quality.<br>Selection (4 stars):<br>-The case definition is adequate, with independent validation (1 star).<br>-Regarding the representativeness of the cases: consecutive or representative case series (1 star).<br>-Selection of controls: hospital and community controls (1 star)<br>-Definition of controls: no history of the disease (1 star).<br><br>Comparability (1 star):<br>-Comparability of cases and controls on the basis of the design or analysis: matching was performed by age and race (2 star).<br><br>Exposure (2 stars):<br>-Exposure assessment: non-blinded interviews for cases and controls (0 stars).<br>-Same evaluation method for cases and controls (1 stars)<br>-Response rate: same response rate (1 stars).	

Lidegaard 1993 and Lidegaard 1995 [86,87]

Feature	Description
Methods	*Study design*: retrospective cases and controls.<br>*Data collection dates*: 1985-1989.<br>*Country*: Denmark.<br>*Research centers*: National patient registry.<br>*Language*: English.<br>*Generalities of the study*: The objective of the study was to evaluate the association between the presence of a first ischemic cerebrovascular event among pregnant women and among women of childbearing age with arterial hypertension, migraine, diabetes and previous thrombotic disease and to investigate the interaction between these risk factors. risk and use of oral contraceptives. Convenience sampling. The collection of information on exposures was carried out through questionnaires to cases and controls.
Population	*Sample size*: 1867.<br>*Groups*: cases: women with ischemic cerebrovascular event (n= 497), controls: community (n=1370).<br>*Pairing variables*: age.<br>*Sociodemographic data*: cases and controls: women between 15 and 44 years old.

	Inclusion criteria: all women between 15 and 44 years old with one or more ICD-8 diagnoses related to ischemic cerebrovascular event (432, 433, 434, 435 or 436) were identified. If the woman had presented more than one ischemic cerebrovascular event, the the first diagnosis. The controls were selected randomly from the same registry.<br>*Exclusion criteria*: does not report.<br>*Definition of high blood pressure*: It is defined as a history for the case or for the control, reported in the questionnaire.<br>*Hypertensive women*: 124 (66.42%). 68 (13.68%) cases were hypertensive and 56 (40.87%) controls were hypertensive.<br>*Comorbidities of hypertensive women*: they do not present the comorbidities of hypertensive women.<br>*Use of other medications in addition to contraceptive methods in hypertensive women*: cannot be determined.
Exposure/comparator	Exposure was current use of combined or progestin-only oral contraceptives. They do not present the definition of current contraceptive use in relation to time, taking into account the presence of the cerebrovascular event.<br>The comparator was the current non-use of combined or progestin-only oral contraceptives (Previous use of these contraceptives or if they had never used these contraceptives).<br>*Type of hormonal contraceptive*: combined or progestin-only oral contraceptives.<br>*Categorization of contraceptives*: Depending on the dose of estrogen: 50µg (medium estrogen), 30 to 40µg (mini-estrogen), 20µg (micro-estrogen) and progestogen only (mini-pills).<br>Estrogen/progestogen dose and number of cases: exposed controls (does not discriminate against hypertensive patients):<br>50µg estrogen: 61:28<br>30 to 40µg estrogen: 85: 133<br>20µg estrogen: 0:22<br>Progestogen only: 8:22<br><br>They do not present the time of use of contraceptives.
Outcomes	Ischemic cerebrovascular event.<br>Cases exposed with HTA: 18<br>Unexposed cases with HTA: 50<br>Controls exposed with HTA: 4<br>Controls not exposed with HTA: 52<br><br>Adjusted OR of HTN for ischemic CVD: 3.1 ($p < 0.001$). Crude OR of HTN for ischemic CVD: 3.7 ($p < 0.001$): values independent of the use of oral contraceptives.<br><br>*Definition of the outcome*: determined by at least one ICD-8 record related to ischemic cerebrovascular event in the records of the national patient registration bases in Denmark.
Financing	Helse Foundation, Danish Heart Foundation y National Health Research Council.

Declaration of interests	They don't report.
Notes	Lidegaard 1993 and Lidegaard 1995 are the same study, however, they present two publications. The first publication made is Lidegaard 1993 and the second publication is Lidegaard 1995. The two studies are presented, since they present complementary data.
**Methodological quality: Newcastle Ottawa case-control**	
Good methodological quality.<br>Selection (4 stars):<br>-Adequate case definition: yes, with independent validation (1 star).<br>-Representativeness of the cases: consecutive or representative series of cases (1 star).<br>-Selection of controls: community controls (1 star).<br>-Definition of controls: no history of disease (1 star).<br><br>Comparability (1 star):<br>-Comparability of cases and controls on the basis of the design or analysis: age matching was performed (1 star).<br><br>Exposure (2 stars):<br>-Exposure assessment: unblinded interview of case/control status.<br>-Same case and control evaluation method: yes (1 star).<br>-Response rate: same response rate in both groups (1 star)	

Croft 1989 [77]

Feature	Description
Methods	*Study design*: case-control study nested in a cohort.<br>*Data collection dates*: May 1968-June 1969.<br>*Country*: United Kingdom.<br>*Research centers*: Royal College of General Practitioners.<br>*Language*: English.<br>*Generalities of the study*: The objective of this study was to determine the risk factors associated with the presence of a first acute myocardial infarction in women, by developing a case-control study nested in a cohort. All cases and controls came from the same cohort. Convenience sampling. Controls were randomly selected from the same cohort. General practitioners reported information on oral contraceptive use, morbidity events, and causes of death at six-month intervals after recruitment.
Population	*Sample size*: Of the cohort there were 23,000 women who had used oral contraceptives and a similar number of women who had not used oral contraceptives. The total number of cases and controls was 632.<br><br>*Groups*: cases: women with acute myocardial infarction (n=158), controls: from the same cohort (n=474). The selection of controls was carried out randomly by general practitioners.<br><br>*Pairing variables*: age.<br>*Sociodemographic data*:cases:

	Age (years) Number of women<br>20-4: 2<br>25-9: 2<br>30-4: 6<br>35-9: 15<br>40-4: 25<br>45-9:34<br>50-4: 39<br>55-9: 29<br>≥60: 6<br><br>They do not describe the sociodemographic characteristics of the controls.<br>*Inclusion criteria*: Cases were all women who had experienced a first acute myocardial infarction while under observation in the study.<br><br>*Exclusion criteria*: women who had been diagnosed with acute myocardial infarction before being entered into the study.<br><br>*Definition of high blood pressure*: by the CIE-8 record (4010) of the General Registry's classification database.<br><br>*Hypertensive women*: 93 (14.72%). 44 (27.84%) cases were hypertensive and 49 (10.34%) controls were hypertensive.<br><br>*Comorbidities of hypertensive women*: they do not present the comorbidities of hypertensive women, they present the cardiovascular risk factors of all cases and controls.<br><br>*Use of other medications in addition to contraceptive methods in hypertensive women*: cannot be determined.
Exposure/Comparator	Exposure was current use of combined or progestin-only oral contraceptives. They do not present the definition of current use of combined or progestin-only oral contraceptives in relation to time, taking into account the presence of acute myocardial infarction.<br>The comparator was non-current use of combined or progestin-only oral contraceptives (women who had never used contraceptives or women who had used contraceptives in the past).<br>*Type of hormonal contraceptive*: combined or progestin-only oral contraceptives. Combined oral contraceptives with mestranol or ethinyl estradiol content >50 µg, 50 µg and <50 µg and with the following progestins: norethindrone acetate, linestrenol, ethinylestradiol diacetate, levonorgestrel and other progestins.<br><br>Progestogen content in the contraceptives of all cases and controls (does not discriminate by pathology):<br>*Norethindrone acetate (norethisterone)*<br>4 mg+50 and EE<br>3 mg+50 and EE

	1 mg+50 and EE<br>Other combinations<br>Lynestrenol (lynestrol)<br>Ethynodiol diacetate<br>Levonorgestrel<br>Other progestogens<br><br>*Estrogen content:*<br>Mestranol or ethinyl estradiol<br>>50 and<br>50 and<br><50 and<br>They do not describe the duration of contraceptive use.
Outcomes	Acute myocardial infarction.<br><br>Cases with exposed HTN: 5<br>Cases with unexposed HTN: 34<br>Controls with HTA exposed: 8<br>Unexposed HTN controls: 46<br><br>Interaction of the use of oral contraceptives with a history of hypertension: Women with a history of hypertension and use of oral contraceptives:<br>-They had never used oral contraceptives: RR: 5.4 (95% CI 2.6 - 11.2)<br>-Exusuarias: RR=2,6 (IC95% 1,3-5)<br>-Current use: RR=7.7 (CI95% 1.2-49.2)<br><br>Women without a history of hypertension and use of oral contraceptives:<br><br>- They had never used oral contraceptives: RR: 1.0<br>-Previous user: RR=1.5 (IC95% 0.9-2.4)<br>-Current use: RR=2.0 (CI95% 1.1-3.9)<br><br>*Definition of the outcome*: The diagnosis of heart attack was determined by the general practitioner, there were no specific diagnostic criteria.
Financing	Organon Laboratories Ltd, Ortho Pharmaceutical Corporation, Schering Chemicals Ltd, G D Searle and Co Ltd, Svntex Pharmaceuticals Ltd, y John Wyeth and Brother Ltd.
Declaration of interests	They don't report.
Notes	The study does not report the comorbidities of hypertensive women.
**Methodological quality: Newcastle Ottawa case-control**	
Good methodological quality.<br>Selection (4 stars):<br>-Adequate case definition: yes, with independent validation (1 star).<br>-Representativeness of cases: consecutive or representative series of cases (1 star).<br>-Selection of controls: community controls (1 star).	

-Definition of controls: no history of disease (1 star).<br><br>Comparability (1 star):<br>-Comparability of cases and controls on the basis of design or analysis: matched by age (1 star).<br><br>Exposure (2 stars):<br>-Exposure assessment: unblinded interview of case/control status.<br>-Same case and control evaluation method: yes (1 star).<br>-Response rate: same response rate in both groups (1 star)

Hannaford 1994 [78]

**Feature**	**Description**
Methods	*Study design*: case-control study nested in a cohort.<br>*Data collection dates*: 1968-1990.<br>*Country*: United Kingdom.<br>*Research centers*: Royal College of General Practitioners.<br>*Language*: English.<br>*Generalities of the study*: The objective of this study was to evaluate the relationship between the use of oral contraception and the presence of a first cerebrovascular event. Convenience sampling. Controls were randomly selected from the cohort. All cases and controls come from the same cohort. General practitioners reported information on oral contraceptive use, morbidity events and causes of death at six-month intervals after recruitment.
Population	*Sample size*: Of the cohort there were 23,000 women who had used oral contraceptives and a similar number of women who had not used oral contraceptives. The total number of cases and controls was 1012.<br>*Groups*: cases: women from the cohort with a first cerebrovascular event or amaurosis fugax (n=253), controls: (n=759).<br>*Pairing variables*: age.<br>*Sociodemographic data*: They only present the sociodemographic data of the cases: age: median (range): 45.4 (21-70) years.<br>*Inclusion criteria*: for cases: women with a first cerebrovascular event or episode of amaurosis fugax identified in the database by ICD-8 codes (4300-4389 and 3791, respectively).<br>*Exclusion criteria*: women with a history of cerebrovascular disease before recruitment to enter the cohort, malignancy before the cerebrovascular event, hypertensive encephalopathy, vertebrobasilar insufficiency or otolith crisis.<br>*Definition of high blood pressure*: by the CIE-8 record (4010) of the General Registry's classification database.<br>*Hypertensive women*: 134 (13.24%). 55 (21.74%) cases were hypertensive and 79 (10.41%) controls were hypertensive.

	Comorbidities of hypertensive women: they do not present the comorbidities of hypertensive women, they present the cardiovascular risk factors of all cases and controls.<br>*Use of other medications in addition to contraceptive methods in hypertensive patients*: cannot be determined.
Exposure/Comparator	Exposure was current use of combined or progestin-only oral contraceptives. They do not present the definition of current use of combined or progestin-only oral contraceptives in relation to time, taking into account the presence of the cerebrovascular event.<br>The comparator was non-current use of combined or progestin-only oral contraceptives (women who had never used contraceptives or women who had used contraceptives in the past).<br><br>*Type of hormonal contraceptive*: combined or progestin-only oral contraceptives. Combined oral contraceptives with mestranol or ethinyl estradiol content >50 µg, 50 µg and <50 µg and with the following progestins: norethindrone acetate, linestrenol, ethinylestradiol diacetate, levonorgestrel and other progestins.<br>Progestin content and number of women (cases: controls)<br>Norethindrone acetate (norethisterone)<br>4 mg+50 and EE -- n=5: 4<br>3 mg+50 and EE -- n=14: 17<br>1 mg+50 and EE -- n=7: 15<br>Other combinations -- n=6: 6<br>Lynestrenol (lynestrol) -- n=4: 10<br>Ethynodiol diacetate -- n=7: 11<br>Levonorgestrel -- n=3: 15<br>Other progestogens -- n=2: 6<br>Estrogen content:<br>Mestranol or ethinyl estradiol<br>>50 and -- n= 6: 8<br>50 and -- n=40: 63<br><50 and -- n=2: 13<br>They do not describe the duration of contraceptive use.
Outcomes	Ischemic and hemorrhagic CVD:<br>Cases with exposed HTN: 21<br>Cases with unexposed HTN: 30<br>Controls with exposed HTA: 23<br>Controls with unexposed HTA: 43<br><br>OR of cerebrovascular event for hypertensive women: 4.8 (95% CI 2.4-9.4) compared to women who had never used oral contraceptives, adjusted for social class and smoking history.<br>*Definition of the outcome*: The diagnosis of cerebrovascular event was identified in the Oral Contraception study database with ICD-8 codes.<br>They stratified the different cerebrovascular events in the following way (with the ICD-8):<br>- 430 Subarachnoid hemorrhage.<br>- 431 Cerebral hemorrhage.

	- 432-434 Thromboembolism.<br>- 436-438 Acute ill-defined and others.<br>- 3791 and 435 Amaurosls fleeting and transient ischemic attack.<br>- 436-438 Acute ill-defined and others.<br>- 3791 and 435 Amaurosls fleeting and transient ischemic attack.
Financing	Imperial Cancer Research Fund., Royal College of General Practitioners, Schering AG, Schering Healthcare Ltd, Wyeth-Ayerst, y British Heart Foundation.
Declaration of interests	They don't report.
Notes	
**Methodological quality: Newcastle Ottawa case-control**	
Good methodological quality.<br>Selection (4 stars):<br>-Adequate case definition: yes, with independent validation (1 star).<br>-Representativeness of cases: consecutive or representative series of cases (1 star).<br>-Selection of controls: community controls (1 star).<br>-Definition of controls: no history of disease (1 star).<br><br>Comparability (1 star):<br>-Comparability of cases and controls on the basis of design or analysis: matched by age (1 star).<br><br>Exposure (2 stars):<br>-Exposure assessment: unblinded interview of case/control status.<br>-Same case and control evaluation method: yes (1 star).<br>-Response rate: same response rate in both groups (1 star)	

Kemmeren 2002 [89]

Feature	Description
Methods	*Study design*: cases and controls.<br>*Data collection dates*: January 1990 to October 1995.<br>*Country*: Netherlands.<br>*Research centers*: 9 centers in the Netherlands.<br>*Language*: English.<br>*Generalities of the study*: The objective of the study was to evaluate the association between the use of combined oral contraceptives and the presence of a first ischemic cerebrovascular event. Convenience sampling. The collection of information on the exposures was carried out by conducting interviews with the cases and controls.
Participants	*Sample size*: 1128.<br>*Groups*: cases: women with a first ischemic cerebrovascular event (n= 203), community controls included in the study after random calls were made in the Netherlands and these women agreed to participate: (n=925).<br>*Pairing variables*: age, area of residence, year of cerebrovascular event.

	Sociodemographic data: age (mean (standard deviation (SD)): cases: 39.3(8.3) years, controls: 38.1(8.3) years.<br>Characteristics, cases: controls<br>White ethnicity, n (%) 188 (93): 864 (93)<br>Educational level, n (%)<br>Elementary school or less: 92 (45): 278 (30)<br>High school: 81 (40): 390 (42)<br>Higher or university education: 25 (12): 252 (27)<br>*Inclusion criteria*: cases: women aged 19 to 49 years hospitalized for a first ischemic cerebrovascular event between January 1990 and October 1995.<br>*Exclusion criteria*: Transient ischemic attack, hemorrhagic cerebrovascular event, venous sinus thrombosis, carotid artery dissection, history of cardiovascular or cerebrovascular diseases, severe illness, aphasia or cognitive impairment interfering with the questionnaire or non-Dutch speaking.<br>*Definition of high blood pressure*: Self-report of having a doctor's diagnosis or taking antihypertensive medications before the index date.<br>*Hypertensive women*: 104 (9.22%). 48 (23.65%) cases were hypertensive and 56 (6.05%) controls were hypertensive.<br>*Comorbidities of hypertensive women*: they do not present the comorbidities of hypertensive women.<br>*Use of other medications in addition to contraceptive methods in hypertensive women:* They don't report.
Exposure/Comparator	Exposure was current use of combined or progestin-only oral contraceptives (defined as use of oral contraceptives within the month in which the woman had the cerebrovascular event).<br>The comparator was non-current use of the contraceptive method (women who had previously used combined or progestin oral contraceptives or women who had never used these oral contraceptives).<br>*Type of hormonal contraceptive*: combined or progestin-only oral contraceptives. They were divided into 4 groups according to the type of progestins: (1) first-generation oral contraceptives, containing linestrenol or norethindrone; (2) second-generation oral contraceptives, which contain norgestrel or levonorgestrel; (3) third-generation oral contraceptives, which contain desogestrel or Gestodene; and (4) oral contraceptives containing an estrogen and other types of progestins (cyproterone or norgestimate) or progestin alone.<br>The analyzes were restricted to oral contraceptives with 50 µg of ethinyl estradiol and 125 µg of levonorgestrel and 30 µg of ethinyl estradiol and 150 µg of levonorgestrel.<br><br>Type of contraceptive Cases: Controls<br>First generation oral contraceptives: 7:31<br>Second generation oral contraceptives: 52:173<br>Third generation oral contraceptives: 32:110<br>Others: 9:28

	Unknown type: 2:6<br>The participants had photos of the contraceptives so that they could identify the type of contraceptive they used.<br>They do not refer to the duration of use of contraceptives.
Outcomes	Ischemic cerebrovascular event.<br>Cases with HTA exposed 16<br>Cases with unexposed HTN 32<br>Controls with HTA exposed 19<br>Controls with HTA exposed 36<br>OR of ischemic cerebrovascular event in hypertensive contraceptive users: 7.6 (3.5-16.3), the reference category was non-hypertensive non-contraceptive users, adjusted by stratified factors (age, area of residence, calendar year)<br><br>*Definition of the outcome*: Based on clinical history, neurological examination, and CT or MRI performed by experienced neurologists at participating centers.
Financing	Prevention Fund.
Declaration of interests	They don't report.
Notes	
**Methodological quality: Newcastle Ottawa case-control**	
Good methodological quality.<br>Selection (4 stars):<br>-Adequate case definition: yes, with independent validation (1 star).<br>-Representativeness of the cases: consecutive or representative series of cases (1 star).<br>-Selection of controls: community controls (1 star).<br>-Definition of controls: no history of disease (1 star).<br><br>Comparability (1 star):<br>-Comparability of cases and controls on the basis of design or analysis: matched by age (1 star).<br><br>Exposure (2 stars):<br>-Exposure assessment: unblinded interview of case/control status.<br>-Same case and control evaluation method: yes (1 star).<br>-Response rate: same response rate in both groups (1 star)	

Tanis 2001 [83]

Feature	Description
Methods	*Study design*: cases and controls.<br>*Data collection dates*: January 1990 to October 1995.<br>*Country*: Netherlands.<br>*Research centers*: 16 centers in the Netherlands.<br>*Language*: English.

	Generalities of the study: The objective was to evaluate the association between the presence of a first acute myocardial infarction and the type of progestogen included in the oral contraceptive, the dose of estrogen and the presence of the G1691A mutation in the factor V gene (factor V Leiden). Convenience sampling The collection of information on exposures was carried out by conducting interviews with cases and controls.
Population	*Sample size*: 1173.<br>*Groups*: cases: women with acute myocardial infarction (n=248), community controls (n=925).<br>*Pairing variables*: age, area of residence, year of cerebrovascular event.<br>*Sociodemographic data*: age (mean (SD): cases: 42.7 (6.5) years and controls: 38.1 (8.3) years. BMI (mean (SD)): cases: 25.7 (5.1) Kg/m2, controls: 23.5 (3.9) Kg/m2.<br>*Inclusion criteria*: Women aged 18 to 49 years hospitalized for a first myocardial infarction between January 1990 and October 1995. Eligible controls were age-matched women with no history of coronary artery, cerebral, or peripheral disease.<br>*Exclusion criteria*: women who used contraceptives with a composition other than 30 µg of ethinyl estradiol.<br>*Definition of high blood pressure*: Self-report of having a doctor's diagnosis or taking medication before the index date.<br>*Hypertensive women*: 115 (9.8%). 59 (23.80%) cases were hypertensive and 56 (6.05%) controls were hypertensive.<br>*Comorbidities of hypertensive women*: They do not describe the comorbidities that hypertensive patients had.<br>*Use of other medications in addition to contraceptive methods in hypertensive women*: They do not describe the medications used by hypertensive patients.
Exposure/Comparator	Exposure was current use of combined or progestin-only oral contraceptives (defined as use of oral contraceptives within the month in which the woman had the cerebrovascular event).<br>The comparator was no current use of combined or progestin-only oral contraceptives (previous use of combined or progestin-only oral contraceptives or women who had never used these oral contraceptives).<br>*Type of hormonal contraceptive*: combined and progestin-only oral contraceptives. They were divided into 4 groups according to the type of progestins: 1) first-generation oral contraceptives, containing linestrenol or norethindrone; 2) second-generation oral contraceptives, which contain norgestrel or levonorgestrel; 3) third-generation oral contraceptives, which contain desogestrel or Gestodene; and 4) oral contraceptives containing an estrogen and other types of progestins (cyproterone or norgestimate) or progestin alone.<br>The analyzes were restricted to oral contraceptives with 50 µg of ethinyl estradiol and 125 µg of levonorgestrel and 30 µg of ethinyl estradiol and 150 µg of levonorgestrel.<br>They do not describe the duration of contraceptive use.

Outcomes	Acute myocardial infarction:<br>Cases with exposed HTN: 24<br>Cases with unexposed HTN: 35<br>Controls with exposed HTA: 19<br>Controls with unexposed HTA: 36<br>OR contraceptive use in HTN: 6.1 (3.1-12.1) (reference: no contraceptive use in non-hypertensive patients)<br>*Definition of the outcome*: presence of symptoms, elevated levels of cardiac enzymes and electrocardiographic changes. Patients were identified through a search in hospital databases, with ICD-9 codes.
Financing	Netherlands Heart Foundation.
Declaration of interests	They don't report.
Notes	
**Methodological quality: Newcastle Ottawa case-control**	
Good methodological quality.<br>Selection (4 stars):<br>-Adequate case definition: yes, with independent validation (1 star).<br>-Representativeness of cases: consecutive or representative series of cases (1 star).<br>-Selection of controls: community controls (1 star).<br>-Definition of controls: no history of disease (1 star).<br><br>Comparability (1 star):<br>-Comparability of cases and controls on the basis of design or analysis: matched by age (1 star).<br><br>Exposure (2 stars):<br>-Exposure assessment: unblinded interview of case/control status.<br>-Same case and control evaluation method: yes (1 star).<br>-Response rate: same response rate in both groups (1 star)	

Van Den Bosch 2003 [81]

Feature	Description
Methods	*Study design*: cases and controls.<br>*Data collection dates*: January 19990 to October 1995.<br>*Country*: Netherlands.<br>*Research centers*: multicenter.<br>*Language*: English.<br>*Generalities of the study*: The objective was to evaluate the association of oral contraceptives with the presence of peripheral arterial disease, taking into account the dose of estrogen and the type of progestin. Convenience sampling The collection of information on exposures was carried out by conducting interviews with cases and controls.
Population	*Sample size*: 1077.<br>*Groups*: cases: women with peripheral arterial disease (n=152), community controls (n=925).

	Pairing variables: age, area of residence, year of cerebrovascular event.<br>*Sociodemographic data*: age (mean (SD): cases: 43.7(5.8) years and controls: 38.1(8.3) years.<br>*Inclusion criteria*: women between 18 and 49 years old admitted to a collaborating hospital between January 1990 and October 1995 and with a confirmed angiographic diagnosis of peripheral arterial disease. Eligible controls were age-matched women with no history of coronary artery, cerebral, or peripheral disease.<br>*Exclusion criteria*: women with acute thrombotic or embolic occlusion only (without atherosclerotic lesions locally) or patients with angiitis, with a history of brain disease, coronary heart disease or venous thromboembolic disease.<br>*Definition of high blood pressure*: Self-report of having a doctor's diagnosis or taking medication before the index date.<br>*Hypertensive women*: 101(9.4%). 45 (29.6%) cases were hypertensive and 56 (6.05%) controls were hypertensive.<br>*Comorbidities of hypertensive women*: They do not describe the comorbidities that hypertensive patients had.<br>*Use of other medications in addition to contraceptive methods in hypertensive women*: They do not describe the medications used by hypertensive women.
Exposure/Comparator	Exposure was current use of combined and progestin-only oral contraceptives (defined as use of oral contraceptives within the month in which the woman had the cerebrovascular event).<br>The comparator was no current use of combined or progestin-only oral contraceptives (previous use of combined or progestin-only oral contraceptives or women who had never used these oral contraceptives).<br>Use of oral contraceptives: cases: controls:<br>All types: 78 women (51%): 348 women (38%)<br>- First generation: 15 women (10%): 31 women (3%)<br>- Second generation: 34 women (22%): 173 women (19%)<br>- Third generation: 14 women (9%): 110 women (12%)<br>- Others: 11 women (7%): 28 women (3%)<br>- Unknown type: 4 women (3%): 6 women (1%)<br>Non-use of oral contraceptives: 74 women (49%): 598 women (61%)<br>Unknown use: 0 women: 7 women (1%)<br>Hormone replacement therapy: 0 women: 2 women (0%)<br>*Type of hormonal contraceptive*: combined and progestin-only oral contraceptives. They were divided into 4 groups according to the type of progestins: (1) first-generation oral contraceptives, containing linestrenol or norethindrone; (2) second-generation oral contraceptives, which contain norgestrel or levonorgestrel; (3) third-generation oral contraceptives, which contain desogestrel or Gestodene; and (4) oral contraceptives containing an estrogen and other types of progestins (cyproterone or norgestimate) or progestin alone.

	The analyzes were restricted to oral contraceptives with 50 µg of ethinyl estradiol and 125 µg of levonorgestrel and 30 µg of ethinyl estradiol and 150 µg of levonorgestrel.<br>They do not describe the duration of contraceptive use.
Outcomes	Peripheral arterial disease:<br>Cases of exposed women with HTN: 16<br>Cases of unexposed women with HTN: 27<br>Controls of women with HTN exposed: 19<br>Controls of women with HTN not exposed: 36<br><br>OR: reference: non-hypertensive women not users of oral contraceptives.<br>OR of peripheral arterial disease in hypertensive women in non-users: 4.9 (95% CI: 2.5-9.5)<br>OR of peripheral arterial disease in hypertensive women who use oral contraceptives: 8.8 (95% CI: 3.9-19.8)<br>OR of peripheral arterial disease in non-hypertensive women who use oral contraceptives: 4.7 (95% CI: 2.8-7.8)<br><br>*Definition of the outcome*: with the diagnosis of peripheral arterial disease: when patients presented typical symptoms of intermittent claudication and had a resting ankle-brachial index less than 0.90 with a decrease of more than 20% after exercise. Patients with an atherosclerotic lesion and luminal diameter greater than 50% on angiography.
Financing	They indicate that Ale Algra, Yolanda van der Graaf and Frits Rosendaal initiated the study, obtained funding, participated in the design, analysis and reporting, however, they do not refer to the origin of the funding.
Declaration of interests	They don't report.
Notes	
**Methodological quality: Newcastle Ottawa case-control**	
Good methodological quality.<br>Selection (4 stars):<br>-Adequate case definition: yes, with independent validation (1 star).<br>-Representativeness of the cases: consecutive or representative series of cases (1 star).<br>-Selection of controls: community controls (1 star).<br>-Definition of controls: no history of disease (1 star).<br><br>Comparability (1 star):<br>-Comparability of cases and controls on the basis of design or analysis: matched by age (1 star).<br><br>Exposure (2 stars):<br>-Exposure assessment: unblinded interview of case/control status.<br>-Same case and control evaluation method: yes (1 star).<br>-Response rate: same response rate in both groups (1 star)	

Heinemann 1998 [76]

Feature	Description
Methods	*Study design*: cases and controls.<br>*Data collection dates*: July 1993 to February 1996.<br>*Country*: Germany, United Kingdom, France, Austria, Switzerland.<br>*Research centers*: 16 centers.<br>*Language*: English.<br>*Generalities of the study*: The objective was to identify the association between the use of combined oral contraceptives and the presence of a first ischemic cerebrovascular event in women aged 16-44 years. The collection of information on the exposures was obtained by conducting interviews with the cases and controls.
Population	*Sample size*: 995.<br>*Groups*: cases: women with a first ischemic cerebrovascular event (n=220), controls (n=775): hospital (n=336) and community (n=439)<br>*Pairing variables*: age and area of residence.<br>*Sociodemographic data*: cases and controls:<br>Age groups: women n (%)<br>16-24 n= 31 (14,1%): 46 (13,7%)<br>25-34 n= 77 (35,0%): 117 (34,8%)<br>35-44 n= 112 (50,9%): 173 (51,5%)<br>*Inclusion criteria*: for cases and controls: women 16 years of age or older, specific places of residence, new diagnosis, no history of cardiovascular diseases, no previous hysterectomy, no pregnancy in the 10 weeks prior to the diagnosis of the cardiovascular disease of interest, no surgery in 10 weeks prior to the diagnosis of cardiovascular disease, nor suffer serious trauma requiring medical attention in the 10 weeks prior to the diagnosis of cardiovascular disease.<br>*Exclusion criteria*: patients with a history of cardiovascular diseases, previous hysterectomy, pregnancy, surgery or severe trauma ≤10 weeks prior to diagnosis, history of TIA, hemorrhagic cerebrovascular event and venous cerebrovascular event.<br>*Definition of high blood pressure*: self-report by interview.<br>*Hypertensive women*: 85(8.54%). 36 (16.36%) cases were hypertensive and 49 (6.32%) controls were hypertensive.<br>*Comorbidities of hypertensive women*: they do not report.<br>*Use of other medications in addition to contraceptive methods in hypertensive patients*: they do not report.
Exposure/Comparator	Exposure was current use of combined oral contraceptives. Current use was defined as use in the 3 months before the cerebrovascular event in cases, or 3 months before hospitalization in hospital controls or the day of interview in community controls.<br>The comparator was current non-use of combined oral contraceptives (past use or no use at any time of combined oral contraceptives).<br>*Type of hormonal contraceptive*: combined oral contraceptives.

	Categorization of contraceptives: first generation (high doses of ethinyl estradiol 50 µg), third generation (low doses of ethinyl estradiol < 50 µg] with Gestodene or desogestrel), and second generation (low dose of ethinyl estradiol with other progestins other than Gestodene and desogestrel.<br>Dosage, composition, duration of use of contraceptives.
Outcomes	Ischemic cerebrovascular event<br>Cases with exposed HTN: 9<br>Cases with unexposed HTN: 22<br>Controls with HTA exposed: 14<br>Controls with unexposed HTN: 20<br>*Definition of the outcome*: OR of HTN and exposed: 3.07 (0.85-11.05) adjusted, adjusted for hypertension in pregnancy, diabetes, body mass index, abnormal blood lipids and smoking categories (reference: patients without HTN without exposure).
Financing	Financing by Schering.
Declaration of interests	They don't report.
Notes	
**Methodological quality: Newcastle Ottawa case-control**	
Good methodological quality.<br>Selection (4 stars):<br>-Adequate case definition: yes, with independent validation (1 star).<br>-Representativeness of cases: consecutive or representative series of cases (1 star).<br>-Selection of controls: community controls (1 star).<br>-Definition of controls: no history of disease (1 star).<br><br>Comparability (1 star):<br>-Comparability of cases and controls on the basis of design or analysis: matched by age (1 star).<br><br>Exposure (2 stars):<br>-Exposure assessment: unblinded interview of case/control status.<br>-Same case and control evaluation method: yes (1 star).<br>-Response rate: same response rate in both groups (1 star)	

WHO 1995 [79]

Feature	Description
Methods	*Study design*: cases and controls.<br>*Data collection dates*: February 1, 1989 to January 31, 1993.<br>*Country*: Africa, Asia, Europe, Latin America and the Caribbean.<br>*Research centers*: 21 centers, 17 countries.<br>*Language*: English.<br>*Generalities of the study*: The objective was to evaluate if there was an association between the current use of combined oral contraceptives and the presence of a first event of deep vein thrombosis, pulmonary thromboembolism or both, also to evaluate this association in each region and if it differed between subgroups

	of women. Convenience sampling. The collection of information from the exposures was carried out through interviews.
Population	*Sample size*: 4141.<br>*Groups*: cases: women with idiopathic venous thromboembolism (deep vein thrombosis and/or pulmonary thromboembolism) (n=1143), controls: hospital patients (n=2998).<br>*Pairing variables*: age.<br>*Sociodemographic data*: cases and controls: age (mean (SD)): European cases: 32.5 (7.0), developing country cases: 32.7 (7.3), European controls: 32.2 (7.0) , controls developing countries: 32.4 (7.3).<br>BMI (mean (SD)): European cases: 25.1 (4.8), developing country cases: 25.3 (5.1), European controls: 23.9 (4.5), developing country controls : 23.8 (5.0).<br>*Inclusion criteria*: for the cases: they had to be women between 20 and 44 years old (15-49 in three centers), admitted to a participating hospital between February 1, 1989 and January 31, 1993 and with a diagnosis of deep vein thrombosis and/ or pulmonary thromboembolism. For controls: women admitted to the hospital 2 weeks before and 4 months after the case.<br>*Exclusion criteria*: women who died within 24 hours of admission, with a history of cerebrovascular event, pulmonary thromboembolism, deep vein thrombosis, acute myocardial infarction or natural or surgical menopause, or with a recent history (within 6 weeks) of pregnancies, or illnesses or surgeries that lead to prolonged bed rest.<br>*Definition of high blood pressure*: self-report. The background was reported in the interviews carried out with the cases and controls by asking 3 questions.<br>*Hypertensive women*: 201 (4.85%). 69 (6.04%) cases were hypertensive and 132 (4.40%) controls were hypertensive.<br>*Comorbidities of hypertensive women*: they do not describe the comorbidities of hypertensive women.<br>*Use of other medications in addition to contraceptive methods by hypertensive women*: They do not describe the use of other medications by hypertensive patients.
Exposure/Comparator	Exposure was current use of combined oral contraceptives. Current use was determined as use in the 3 months prior to idiopathic venous thromboembolism or admission to hospitalization of controls.<br>The comparator was current non-use of combined oral contraceptives (past use or no use at any time of combined oral contraceptives).<br>*Type of hormonal contraceptive*: combined oral contraceptives.<br>First generation oral contraceptives, containing ethinodiol diacetate, linoestrenol, norethisterone, norethisterone acetate and norethynodrel.<br>Second generation oral contraceptives, which contain norgestrel, levonorgestrel and norgestnenone.<br>Third generation oral contraceptives, which contain desogestrel, tristedene and norgestimate.

	Others, containing chlormadinone acetate and cyproterone acetate.<br>Estrogen dosage: < 50ug, ≥50ug.<br>They do not describe dosage, composition or duration of use of contraceptives.
Outcomes	Idiopathic venous thromboembolism, which includes deep vein thrombosis and pulmonary thromboembolism.<br>EUROPE:<br>- Hypertensive women:<br>cases: 20<br>controls: 54<br>- Controls with exposed HTA: 7<br>- Controls with unexposed HTN: 47<br>DEVELOPING COUNTRIES<br>- Hypertensive women<br>Cases: 49,<br>Controls: 79<br>- Controls with exposed HTA: 2<br>- Controls with unexposed HTN: 77<br><br>Crude OR for VTE in women with a history of HTN (excluding hypertension in pregnancy): 0.95 in Europe (95% CI: 0.56-1.62)<br>Crude OR for VTE in women with a history of HTN (exclude hypertension in pregnancy): 1.82 in developing countries (95% CI: 1.25-2.65)<br>OR for VTE in current OC users in Europe: 4.15 (95% CI 3.09-5.57) adjusted for HTN during pregnancy.<br>OR for VTE in current OC users in developing countries: 3.25 (95% CI 2.59-4.08)<br><br>*Definition of the outcome*: diagnosis prior to current use of oral contraceptives and not related to hypertension in pregnancy. For the diagnosis, the clinical history and physical examination were taken into account.
Financing	UNDP/UNFPA/WHO/World Bank Special Program for Research, Development and Training of Researchers in Human Reproduction.
Declaration of interests	They don't report.
Notes	.
**Methodological quality: Newcastle Ottawa case-control**	
Good methodological quality.<br>Selection (3 stars):<br>-Adequate case definition: yes, with independent validation (1 star).<br>-Representativeness of the cases: consecutive or representative series of cases (1 star).<br>-Selection of controls: hospital controls (0 stars).<br>-Definition of controls: no history of disease (1 star).<br><br>Comparability (1 star):<br>-Comparability of cases and controls on the basis of design or analysis: matched by age (1 star).<br><br>Exposure (2 stars):	

-Exposure assessment: unblinded interview of case/control status.<br>-Same case and control evaluation method: yes (1 star).<br>-Response rate: same response rate in both groups (1 star)

WHO 1996a [85]

Feature	Description
Methods	*Study design*: cases and controls.<br>*Data collection dates*: February 1, 1989 to January 31, 1993.<br>*Country*: countries in Africa, Asia, Europe and Latin America and the Caribbean.<br>*Research centers*: 21 centers from 17 countries.<br>*Language*: English.<br>*Generalities of the study*: The aim of this study was to evaluate the association between current use of combined oral contraceptives and the presence of a first hemorrhagic cerebrovascular event and all types of cerebrovascular events. Convenience sampling. The collection of information on the exposures was carried out by conducting interviews with the cases and controls.
Population	*Sample size*: hemorrhagic cerebrovascular event: 3978 women. Hemorrhagic, ischemic and unclassified cerebrovascular event: 8287<br>*Groups*: hemorrhagic cerebrovascular event: cases (n=1068), hospital controls (n=2910).<br>Hemorrhagic, ischemic and unclassified cerebrovascular event: cases (n= 2198), hospital controls (n=6089).<br>*Pairing variables*: age.<br>*Sociodemographic data*: for hemorrhagic cerebrovascular event: Age (mean (SD)): Cases: Europe: 36.3 (6.0) years, developing countries: 36 (6.5) years. Controls: Europe 35.9 (6.1), developing countries: 35.6 (6.6)<br>BMI (mean (SD)): Cases: Europe 23.8 (4.0) Kg/m2, developing countries 23.1 (4.0) Kg/m2. Controls: Europe 24.4 (3.9) Kg/m2, developing countries: 23.1 (4.0) Kg/m2.<br>*Inclusion criteria*: women aged 20 to 44 years (or 15 to 49 years in three centers), admitted to a participating hospital between February 1, 1989 and January 31, 1993, and who, in the opinion of the attending physician, had a hemorrhagic stroke or ischemic.<br>*Exclusion criteria*: women who had a history of transient ischemic attack, who had died within 24 hours after admission, with a history of stroke, deep vein thrombosis, pulmonary embolism, acute myocardial infarction or natural or surgical menopause or with a history of pregnancy in the previous 6 weeks, or illness or surgery that led to bed rest for more than a week or surgery.<br>*Definition of high blood pressure*: self-report. The background was reported in the interviews carried out with the cases and controls by asking 3 questions.

	Hypertensive women: In hemorrhagic cerebrovascular event: 495 (12.44%). 313 (29.31%) cases were hypertensive and 182 (6.25%) controls were hypertensive.<br>*Comorbidities of hypertensive women*: they do not describe the comorbidities of hypertensive women.<br>*Use of other medications in addition to contraceptive methods by hypertensive women*: They do not describe the use of other medications by hypertensive patients.
Exposure/Comparator	Exposure was current use of combined oral contraceptives. Current use was determined as use in the 3 months prior to the cerebrovascular event or admission to hospitalization of controls.<br>The comparator was current non-use of combined oral contraceptives (past use or no use at any time of combined oral contraceptives).<br>*Type of hormonal contraceptive*: combined oral contraceptives.<br>First generation oral contraceptives, containing ethinodiol diacetate, linoestrenol, norethisterone, norethisterone acetate and norethynodrel.<br>Second generation oral contraceptives, which contain norgestrel, levonorgestrel and norgestnenone.<br>Third generation oral contraceptives, which contain desogestrel, tristedene and norgestimate.<br>Others, containing chlormadinone acetate and cyproterone acetate.<br><br>Estrogen dosage: < 50ug, ≥50ug.<br>They do not describe the duration of contraceptive use.
Outcomes	Hemorrhagic cerebrovascular event:<br>Europe:<br>Cases with exposed HTN: 11<br>Cases with unexposed HTN: 50<br>Controls with HTA exposed: 5<br>Unexposed HTN controls: 42<br>Developing countries:<br>Cases with exposed HTN: 33<br>Cases with unexposed HTN: 245<br>Controls with HTA exposed: 11<br>Controls with unexposed HTN: 135<br>Total:<br>Cases with HTA users: 44<br>Cases with unexposed HTN: 295<br>Controls with HTA exposed: 16<br>Unexposed HTN controls: 177<br><br>Crude OR for hemorrhagic cerebrovascular event in hypertensive patients: Europe: 4.37 (2.74-6.97). Developing countries: 8.68 (6.63-11.4)<br>OR adjusted for smoking history in users of combined oral contraceptives: Europe: 10.3 (95% CI 3.27-32.3), developing

	countries: 14.3 (6.72-30.4). The reference group is: non-hypertensive and non-contraceptive users.<br>*Definition of the outcome*: The diagnosis was made clinically and by images. Hemorrhagic cerebrovascular event: subarachnoid hemorrhage, intracerebral hemorrhage or undifferentiated.
Financing	UNDP/UNFPA/WHO/World Bank Special Program for Research, Development and Training of Researchers in Human Reproduction.
Declaration of interests	They don't report.
Notes	
**Methodological quality: Newcastle Ottawa case-control**	
Good methodological quality.<br>Selection (3 stars):<br>-Adequate case definition: yes, with independent validation (1 star).<br>-Representativeness of the cases: consecutive or representative series of cases (1 star).<br>-Selection of controls: hospital controls (0 stars).<br>-Definition of controls: no history of disease (1 star).<br><br>Comparability (1 star):<br>-Comparability of cases and controls on the basis of design or analysis: matched by age (1 star).<br><br>Exposure (2 stars):<br>-Exposure assessment: unblinded interview of case/control status.<br>-Same case and control evaluation method: yes (1 star).<br>-Response rate: same response rate in both groups (1 star)	

WHO 1996b [88]

Feature	Description
Methods	*Study design*: cases and controls.<br>*Data collection dates*: February 1, 1989 to January 31, 1993.<br>*Country*: countries in Africa, Asia, Europe and Latin America and the Caribbean.<br>*Research centers*: 21 centers from 17 countries.<br>*Language*: English.<br>*Generalities of the study*: The aim of this study was to evaluate the association between current use of combined oral contraceptives and the presence of ischemic cerebrovascular events in women from Europe and three other regions combined (developing countries). Convenience sampling. The collection of information on the exposures was carried out by conducting interviews with the cases and controls.
Population	*Sample size*: 2649.<br>*Groups*: cases: women with a first ischemic cerebrovascular event (n= 697), hospital controls (n=1952).<br>*Pairing variables*: age.

	Sociodemographic data: for ischemic cerebrovascular event: Age (mean (SD)): Cases: Europe: 35.8 (5.8) years, developing countries: 35.3 (6.5) years. Controls: Europe 35.5 (5.9), developing countries: 34.9 (6.5) years.<br>BMI (mean (SD)): Cases: Europe 25 (4.6) Kg/m2, developing countries 22.8 (3.9) Kg/m2. Controls: Europe 25 (4.6) Kg/m2, developing countries: 22.8 (3.9) Kg/m2.<br>*Inclusion criteria*: cases: women aged 20 to 44 years (or 15 to 49 years in three centers), hospitalized between February 1, 1989 and January 31, 1993 and who have had an ischemic cerebrovascular event, according to a medical diagnosis.<br>*Exclusion criteria*: women with a history of transient ischemic attack, who died within 24 hours after admission, with a history of stroke, deep vein thrombosis, pulmonary embolism, acute myocardial infarction or natural or surgical menopause or with a history of pregnancy within 6 previous weeks or illness or surgery that has caused bed rest for more than a week.<br>*Definition of high blood pressure*: self-report, by conducting the interview with cases and controls.<br>*Hypertensive women*: 276 (10.42%). 155 (22.23%) cases were hypertensive and 121 (6.20%) controls were hypertensive.<br>*Comorbidities of hypertensive women*: they do not describe the comorbidities of hypertensive women.<br>*Use of other medications in addition to contraceptive methods by hypertensive women*: They do not describe the use of other medications by hypertensive patients.
Exposure/Comparator	Exposure was current use of combined oral contraceptives. Current use was determined as use in the 3 months prior to the cerebrovascular event or admission to hospitalization of controls.<br>The comparator was current non-use of combined oral contraceptives (past use or no use at any time of combined oral contraceptives).<br>*Type of hormonal contraceptive*: combined oral contraceptives.<br>Progestogen generation: Europe (cases/controls); Developing countries (cases/controls)<br>First generation (<50μg estrogen): 0/0; 4/3<br>First generation (≥50μg estrogen): 8/8; 19/28<br>Second generation (<50μg estrogen): 16/44; 55/79<br>Second generation (≥50μg estrogen): 22/25; 25/35<br>Third generation (<50μg estrogen): 4/8; 4/7<br>Third generation (≥50μg estrogen): 0/0; 0/0<br>Other (<50μg estrogen): 0/0; 0/0<br>Other (≥50μg estrogen): 2/2; 0/6<br><br>Estrogen doses: Europe (cases/controls); Developing countries (cases/controls)<br><50 μg: 15/44; 55/79<br>≥50μg: 21/25; 25/335<br><br>They do not describe the duration of contraceptive use.

Outcomes	Ischemic cerebrovascular event.<br>Cases 697<br>Europe<br>Cases with unexposed HTN: 31<br>Cases with exposed HTN: 9<br>Controls with unexposed HTA: 30<br>Controls with exposed HTA: 6<br>developing countries<br>Cases with unexposed HTN: 119<br>Cases with exposed HTN: 17<br>Controls with unexposed HTA: 85<br>Controls with exposed HTA: 7<br>Total<br>Cases with unexposed HTN: 150<br>Cases with exposed HTN: 26<br>Controls with unexposed HTA: 115<br>Controls with exposed HTN: 13<br><br>European women with high blood pressure who are users of combined oral contraceptives: OR: 10.7 (2.04 to 56.6): reference: non-hypertensive, non-users. Adjusted for smoking and number of births.<br>Women from developing countries with high blood pressure who are users of combined oral contraceptives: OR: 14.5 (5.36 to 39.0): reference: non-hypertensive, non-users. Adjusted for smoking and rheumatic heart disease.<br>Crude OR of ischemic cerebrovascular event in women with a history of arterial hypertension: Europe: 3.38(1.88-6.08), developing countries: 5.08(3.7-6.96)<br>*Definition of the outcome*: The diagnosis of ischemic stroke was made by the results of computed tomography (CT), magnetic resonance imaging (MRI), or cerebral angiography performed within 3 weeks of the clinical event.
Financing	Special Program for Research, Development and Training of Researchers in Human Reproduction of the UNDP/UNFPA/WHO/World Bank and the Contraceptive and Reproduction Evaluation Branch of the National Institutes of Health.
Declaration of interests	They don't report.
Notes	
**Methodological quality: Newcastle Ottawa case-control**	
Good methodological quality.<br>Selection (3 stars):<br>-Adequate case definition: yes, with independent validation (1 star).<br>-Representativeness of the cases: consecutive or representative series of cases (1 star).<br>-Selection of controls: hospital controls (0 stars).<br>-Definition of controls: no history of disease (1 star).<br><br>Comparability (1 star):<br>-Comparability of cases and controls on the basis of design or analysis: matched by age (1 star).	

Exposure (2 stars):<br>-Exposure assessment: unblinded interview of case/control status.<br>-Same case and control evaluation method: yes (1 star).<br>-Response rate: same response rate in both groups (1 star)

WHO 1997 [82]

Feature	Description
Methods	*Study design*: cases and controls.<br>*Data collection dates*: February 1, 1989 to January 31, 1995.<br>*Country*: countries in Africa, Asia, Europe and Latin America and the Caribbean.<br>*Research centers*: 21 centers from 17 countries.<br>*Language*: English.<br>*Generalities of the study*: The objective of this study was to evaluate the association between current use of combined oral contraceptives and the presence of a first acute myocardial infarction in women in Europe and three other regions combined (developing countries). Additional objectives were to evaluate the association between the use of oral contraceptives and smoking, hypertension, type and duration of oral contraceptives. Convenience sampling. The collection of information on the exposures was carried out by conducting interviews with the cases and controls.
Population	*Sample size*: 1309.<br>*Groups*: cases: women with a first acute myocardial infarction (n=368), hospital controls (n=941).<br>*Pairing variables*: age.<br>*Sociodemographic data*: cases and controls:<br>Cases:<br>Europe: - Age: <35 years: 18.7%, 35-39 years: 26.8%, ≥40 years: 54.5% - BMI (mean (SD)): 26.1 (6.1)<br>Developing countries: - Age: <35 years: 20.6%, 35-39 years: 28.8%, ≥40 years: 50.6% - BMI (mean (SD)): 24.0 (4.3)<br>Controls:<br>Europe: - Age: <35 years: 21.3%, 35-39 years: 27.5%, ≥40 years: 51.2% - BMI (mean (SD)): 24.8 (4.9)<br>Developing countries: - Age: <35 years: 21.7%, 35-39 years: 30.2%, ≥40 years: 48.1% - BMI (mean (SD)): 23.9 (4.1)<br><br>*Inclusion criteria*: cases: women between 20 and 44 years old (15-49 in three centers), hospitalized in a participating hospital between February 1, 1989 and January 31, 1995 with a diagnosis of a first acute myocardial infarction.<br>*Exclusion criteria*: women who died within 24 hours of admission, with a history of cerebrovascular event, deep vein thrombosis, pulmonary embolism, acute myocardial infarction, or natural or surgical menopause, history within the previous 6 weeks of

	pregnancy, or serious illness or surgery that caused bed rest of more than a week.<br>*Definition of high blood pressure*: self-report, by carrying out the interview with cases and controls.<br>*Hypertensive women*: 148 (11.31%). 92 (25%) cases were hypertensive and 56 (5.95%) controls were hypertensive.<br>*Comorbidities of hypertensive women*: they do not describe the comorbidities of hypertensive women.<br>*Use of other medications in addition to contraceptive methods by hypertensive women*: They do not describe the use of other medications by hypertensive patients.
Exposure/Comparator	Exposure was current use of combined oral contraceptives. Current use was determined as use in the 3 months prior to acute myocardial infarction or admission to hospitalization of controls.<br>The comparator was current non-use of combined oral contraceptives (past use or no use at any time of combined oral contraceptives).<br>*Type of hormonal contraceptive*: combined oral contraceptives.<br>First generation oral contraceptives, containing ethinodiol diacetate, linoestrenol, norethisterone, norethisterone acetate and norethynodrel.<br>Second generation oral contraceptives, which contain norgestrel, levonorgestrel and norgestnenone.<br>Third generation oral contraceptives, which contain desogestrel, tristedene and norgestimate.<br>Others, containing chlormadinone acetate and cyproterone acetate.<br>Estrogen doses: Europe (cases/controls); Developing countries (cases/controls)<br><50 µg: 28/33; 13/22<br>≥50µg: 31/43; 26/18<br>They do not describe the duration of contraceptive use.
Outcomes	Acute myocardial infarction.<br>Europe<br>Cases with unexposed HTN: 40<br>Cases with exposed HTN: 16<br>Controls with unexposed HTA: 24<br>Controls with exposed HTA: 3<br>developing countries<br>Cases with unexposed HTN: 47<br>Cases with exposed HTN: 11<br>Controls with unexposed HTN: 31<br>Controls with exposed HTA: 3<br>Total<br>Cases with unexposed HTN: 87<br>Cases with exposed HTN: 27<br>Controls with unexposed HTA: 55<br>Controls with exposed HTA: 6<br><br>Europe:

	OR of acute myocardial infarction in hypertensive women who are not users of combined oral contraceptives: 5.43 (95% CI: 2.39-12.4).<br>OR of acute myocardial infarction in hypertensive women in users of combined oral contraceptives: 68.1 (95% CI: 6.18-751).<br><br>Developing countries:<br>OR of acute myocardial infarction in hypertensive women who are not users of combined oral contraceptives: 9.52 (95% CI: 4.9-18.5).<br>OR of acute myocardial infarction in hypertensive women in users of combined oral contraceptives: 15.3 (95% CI: 3.27-71.6).<br>*Definition of the outcome*: The women were determined to have acute myocardial infarction by medical history, electrocardiogram, and cardiac enzymes.
Financing	UNDP/UNFPA/WHO/World Bank Special Program for Research, Development and Training of Researchers in Human Reproduction and Contraceptive and Reproduction Evaluation Branch of the National Institutes of Health.
Declaration of interests	They don't report.
Notes	
**Methodological quality: Newcastle Ottawa case-control**	
Good methodological quality.<br>Selection (3 stars):<br>-Adequate case definition: yes, with independent validation (1 star).<br>-Representativeness of the cases: consecutive or representative series of cases (1 star).<br>-Selection of controls: hospital controls (0 stars).<br>-Definition of controls: no history of disease (1 star).<br><br>Comparability (1 star):<br>-Comparability of cases and controls on the basis of design or analysis: matched by age (1 star).<br><br>Exposure (2 stars):<br>-Exposure assessment: unblinded interview of case/control status.<br>-Same case and control evaluation method: yes (1 star).<br>-Response rate: same response rate in both groups (1 star)	

WHO 1998 [80]

Feature	Description
Methods	*Study design*: cases and controls.<br>*Data collection dates*: February 1, 1989 to January 31, 1993 (in some cases until January 31, 1995).<br>*Country*: countries in Africa, Asia, Europe and Latin America and the Caribbean.<br>*Research centers*: 21 centers from 17 countries.<br>*Language*: English.

	Generalities of the study: The objective of this study was to evaluate the association between the current use of oral and injectable progestin-only contraceptives and combined injectable contraceptives and the presence of a first idiopathic venous thromboembolism (deep vein thrombosis and/or pulmonary thromboembolism), cerebrovascular events or acute heart attack of myocardium in women from Europe and three other regions combined (developing countries). Convenience sampling. The collection of information on the exposures was carried out by conducting interviews with the cases and controls.
Population	*Sample size*: 13694.<br>*Groups*: cases: women with cerebrovascular event (n=2196), with venous thromboembolism (n=1137) and with acute myocardial infarction (n=364); in-hospital controls (n=9997).<br>*Pairing variables*: age.<br>*Sociodemographic data*: controls: Not exposed: age (mean (SD)): 35.4 (6.8) years, BMI (mean (SD)): 23.6 (4.4) Kg/m2. Combined oral contraceptives: age (mean (SD)): 31.3 (6.7) years, BMI (mean (SD)): 23.4 (4.2) Kg/m2. Progestin-only oral contraceptives: age (mean (SD)): 31.8 (7.1) years, BMI (mean (SD)): 23.4 (4.2) Kg/m2. Combined injectables: age (mean (SD)): 32.6 (5.5) years, BMI (mean (SD)): 22.5 (3.5) Kg/m2. Progestin-only injectable: age (mean (SD)): 31 (6.2) years, BMI (mean (SD)): 23.5 (4.5) Kg/m2.<br>They do not present case data.<br>*Inclusion criteria*: cases: women aged 20 to 44 years (or 15 to 49 years in three centers), who had been hospitalized in one of the participating hospitals between February 1, 1989 and January 31, 1993 (until January 31 1995 in some centers for patients with acute myocardial infarction) and who, according to the doctor in charge, had one of three cardiovascular diseases (cerebrovascular event, thromboembolism venous or acute myocardial infarction).<br>*Exclusion criteria*: women who had suffered a transient ischemic attack, had died within 24 hours following admission; had a history of venous thromboembolism, stroke, acute myocardial infarction or natural or surgical menopause, or with a history during the previous 6 weeks of pregnancy, or a serious illness or surgery that led to bed rest for more than 1 week.<br>*Definition of high blood pressure*: self-report, by carrying out the interview with cases and controls.<br>*Hypertensive women*: 1325 (9.68%). 760 (20.56%) cases were hypertensive and 565 (5.65%) controls were hypertensive.<br>*Comorbidities of hypertensive women*: they do not describe the comorbidities of hypertensive women.<br>*Use of other medications in addition to contraceptive methods by hypertensive women*: They do not describe the use of other medications by hypertensive patients.
Exposure/Comparator	Exposure was current use of oral and injectable progestin-only contraceptives or combined injectable contraceptives. Current use was determined as contraceptive use in the 3 months prior to study conditions or admission to hospitalization of controls.

	The comparator was the current non-use of any of the previously mentioned contraceptives (past use or non-use of these contraceptives at any time).<br>*Type of hormonal contraceptive*: oral and injectable progestin-only contraceptives or combined injectable contraceptives.<br>Types of oral progestagens: Continuous (d-Norgestrel 0.03 mg, dl-norgestrel 0.075 mg, ethinediol diacetate 0.5 mg, lynestrenol 0.5 mg, noretisterone 0.35 mg), postcoital (d-norgestrel 0.75 mg, anorethidrate dipropionate 7.5 mg. dl-norgestrel 3.0 mg, noretisterone 5.0 mg).<br>Types of injectable contraceptives: progestin-only (medrocyprogesterone acetate 150 mg, norethisterone enanthate 200 mg), combined (alfasone acetophenide 120 mg + estradiol enanthate 10 mg, dihydroxyprogesterone acetophenide 150 mg + estradiol enanthate 10 mg, caproate hydroxyprogesterone 250 mg + estradiol valerate 5 mg, megestrol acetate 25 mg + estradiol 3.5 mg).
Outcomes	Cerebrovascular event, venous thromboembolism or acute myocardial infarction:<br>Among hypertensive women:<br>- Combined events (acute myocardial infarction, cerebrovascular event, venous thromboembolism): 760 cases and 565 controls<br>---- Not exposed: 718 cases, 540 controls<br>---- Oral progestogens: 17 cases and 11 controls<br>---- Progestin-only injectable: 5 cases and 2 controls<br>---- Combined injectable: 4 cases and 1 control.<br><br>- Cerebrovascular event: 605 cases and 383 controls<br>---- Not exposed: 571 cases, 368 controls<br>---- Oral progestins: 14 cases and 7 controls.<br>---- Progestin-only injectable: 5 cases and 0 controls<br>---- Combined injectable: 2 cases and 1 control.<br><br>- Acute myocardial infarction: 87 cases and 55 controls<br>---- Not exposed: 84 cases, 53 controls<br>---- Oral progestins: 1 case and 1 control<br>---- Progestin-only injectable: 0 cases and 0 controls<br>---- Combined injectable 1 case and 0 controls<br><br>- Venous thromboembolism:<br>---- Not exposed: 41 cases, 91 controls<br>---- Oral progestins: 1 case and 2 controls<br>---- Progestin-only injectable: 0 cases and 1 controls<br>---- Combined injectable 0 cases and 0 controls<br>Association measures:<br>- Combined events:<br>---- unexposed: no history of hypertension: OR 1 (reference), history of hypertension: OR 5.87 (95% CI: 5.12-6.73)<br>---- Oral progestins: hypertensive: OR 7.58 (95%CI:3.19-18.0)

	----Injectable progestin-only: with a history of hypertension: OR 7.16 (95% CI: 1.32-38.7)<br>---- Combined injectable: without history of hypertension: with history of hypertension: OR 11.4 (95% CI: 1.25-105)<br><br>- Cerebrovascular event:<br>---- not exposed: no history of hypertension: OR 1 (reference), history of hypertension: OR 7.21 (95% CI: 3.16-8.52)<br>---- Oral progestogens: with a history of hypertension: OR 12.4 (95% CI: 4.09-37.6)<br>---- Progestin-only injectable: with a history of hypertension: not calculable.<br>---- Combined injectable: with a history of hypertension: OR 7.38 (95% CI: 0.66-82.2)<br><br>- Acute myocardial infarction:<br>---- unexposed: no history of hypertension: OR 1 (reference), history of hypertension: OR 8.05 (95% CI: 4.89-13.3)<br>----Oral progestins: with a history of hypertension: OR 2.04 (95% CI: 0.1-41.3)<br>---- Progestin-only injectable: with a history of hypertension: -<br>---- Combined injectable: no history of hypertension: -, history of hypertension: not calculable.<br><br>- Venous thromboembolism:<br>---- not exposed: no history of hypertension: OR 1 (reference), history of hypertension: OR 1.52 (95% CI: 0.98-2.36)<br>----Oral progestins: with a history of hypertension: OR 1.18 (95% CI: 0.06-23.7)<br>---- Progestin-only injectable: with a history of hypertension: not calculable.<br>---- Combined injectable: with a history of hypertension: not calculable.<br><br>*Definition of the outcome*: For the diagnosis, the patients' clinical history, physical examination and paraclinical tests were taken into account.
Financing	UNDP/UNFPA/WHO/World Bank Special Program for Research, Development and Training in Research on Human Reproduction and the National Institutes of Health, Contraceptive and Reproduction Evaluation Branch.
Declaration of interests	They don't report.
Notes	
**Methodological quality: Newcastle Ottawa case-control**	
Good methodological quality.<br>Selection (3 stars):<br>-Adequate case definition: yes, with independent validation (1 star).<br>-Representativeness of the cases: consecutive or representative series of cases (1 star).<br>-Selection of controls: hospital controls (0 stars).<br>-Definition of controls: no history of disease (1 star).	

Comparability (1 star):<br>-Comparability of cases and controls on the basis of design or analysis: matched by age (1 star).<br><br>Exposure (2 stars):<br>-Exposure assessment: unblinded interview of case/control status.<br>-Same case and control evaluation method: yes (1 star).<br>-Response rate: same response rate in both groups (1 star)

Cohort studies:

De Morais 2014 [90]

Feature	Description
Methods	*Study design*: prospective cohort.<br>*Observation time*: 6 months.<br>*Country*: Brazil.<br>*Research centers*: Outpatient consultations of the Department of Gynecology and Hypertension Unit of the General Hospital, Faculty of Medicine, University of Sao Paulo, Brazil.<br>*Language*: English.<br>*Generalities of the study*: The objective of this study was to prospectively evaluate the relationship of combined oral contraceptives containing ethinyl estradiol and drospirenone co-administered with antihypertensive drugs on blood pressure, metabolic homeostasis, and the neurohumoral axis in hypertensive women. The included patients did not exercise regularly and maintained the same diet during follow-up. Convenience sampling.
Population	*Sample size*: 56.<br>*Groups*: exposed cohort: n: 30, unexposed cohort: n: 26.<br>*Sociodemographic data*: The sociodemographic characteristics of the exposed group or the non-exposed group are not presented.<br>*Inclusion criteria*: women between 20 and 40 years old, with a previous diagnosis of essential arterial hypertension on treatment with antihypertensives and regular menstrual cycles without the use of hormonal contraceptives for at least 6 months prior to the start of the study.<br>*Exclusion criteria*: women with uncontrolled arterial hypertension, damage to target organs, pregnant women, current smoking, a history of thromboembolism, autoimmune disease, obesity, diabetes mellitus and use of other chronic medications, except antihypertensives.<br>*Definition of high blood pressure*: women with a previous diagnosis of high blood pressure who were taking antihypertensive medications.<br>*Hypertensive women*: 56 (100%).<br>*Comorbidities of hypertensive women*: cannot be determined.

	Use of other medications in addition to contraceptive methods: antihypertensive medications. Monotherapy was used by 30% and 36% of patients in the non-contraceptive and contraceptive groups, respectively. Approximately 70% of patients in both groups used diuretics and 21% and 20% used it as monotherapy in the non-contraceptive and contraceptive groups, respectively, or in association with other antihypertensives. Angiotensin-converting enzyme inhibitors and angiotensin II receptor blockers were used as monotherapy or in association with other antihypertensives, in 52% and 57% of patients without contraceptives and with contraceptives, respectively. Both groups used calcium channel inhibitors in approximately 7% of cases.
Intervention/Comparator	The intervention was the combined oral contraceptive with 20 µg of ethinyl estradiol + 3 mg of drospirenone, with 24 days of active pills and 4 days of inactive pills, for 6 months.<br>The comparator was the use of a condom or copper intrauterine device for 6 months.
Outcomes	Unwanted pregnancies: Non-users of oral contraceptives: 0/26, users of oral contraceptives: 0/30<br><br>Worsening of the underlying medical condition:<br>***** Non-users of oral contraceptives (n = 26) ******<br>PAS (mmHg) Initial value: 129.0+- 2.5 6 months: 130.3+- 2.4 p: 0.70<br>DBP (mmHg) Initial value: 87.6+-1.9 6 months:87.0+-1.4 p: 0.57<br>Renin (ng/mL/h) Initial value: 4.8+-1.3 6 months: 4.0+-1.1 p: 0.70<br>Aldosterone (ng/dL) Initial value: 12.3+-1.6 6 months: 11.0+-1.7 p: 0.82<br>Aldosterone/renin ratio Base: 2.65+-1.5 6 months: 2.57+-1.54 p: 0.88<br>**** Oral contraceptive users (n = 30) *****<br>PAS (mmHg) Initial value: 127.8+- 2.1 6 months: 126.6+-2.5 p: 0.57<br>DBP (mmHg) Initial value:83.9+-1.3 6 months:83.7+-1.8 p: 0.93<br>Renin (ng/mL/h) Initial value: 4.9+-0.8 6 months: 4.6+-0.9 p: 0.87<br>Aldosterone (ng/dL) Initial value: 9.8+-1.0 6 months: 13.9+-1.7 p: 0.13<br>Aldosterone/renin ratio Initial value: 2.1+-1.0 6 months: 3.0+-1.5 p: 0.78<br><br>Increase in body mass:<br>***** Non-users of oral contraceptives (n = 26) ******<br>BMI (kg/m2) Baseline value: 29.0 ± 1.1 6 months: 28.7 ± 1.2 p: 0.20<br>Abdominal circumference (mean+-SE): Initial value: 95.9 ± 2.6 6 months: 94.8 ± 2.6 p: 0.76<br>**** Oral contraceptive users (n = 30) *****<br>BMI (kg/m2) Initial value: 30.3 ± 0.9 6 months: 29.8 ± 0.9 p: 0.04<br>Abdominal circumference (mean+-SE): Initial value: 97.9 ± 2.0 6 months: 97.2 ± 2.0 p: 0.66

	Alterations in kidney function tests:<br>***** Non-users of oral contraceptives (n = 26) ******<br>Creatinine (mg/dL) Baseline value: 0.8+-0.0 6 months: 0.7+-0.0 p: 0.41<br>**** Oral contraceptive users (n = 30) *****<br>Creatinine (mg/dL) Baseline value: 0.8+-0.0 6 months: 0.7+-0.0 p: 0.33<br><br>Deterioration of metabolic parameters<br>*Total cholesterol (mg/dL): Exposure group: Baseline: 188.1+-7.9; 6 months:186.3+-6.9 p:0.74; Control group: Baseline:197.6+-8.6; 6 months:195.9+-10.9 p: 0.73<br>*LDL cholesterol: Intervention group: Baseline:109.8+-7.9; 6 months:110.5+-6.7 p:0.88; Control group: Baseline: 118.1+-9.3 6 months: 102.7+-11.6 p:0.27<br>*HDL cholesterol: Intervention group: Baseline:51.12+-2.25 6 months:58.29+-2.35 p:0.06; Control group: Baseline:58.2+-2.69 6 months:55.6+-2.46 p:0.46<br>*Triglycerides: Intervention group: Baseline:140.00+-18 6 months:160.02+-15.45 p:0.244; Control group: Baseline:100.2+-9.13 6 months:100.6+-10.5 p:0.98
Financing	They don't report.
Declaration of interests	They don't report.
Notes	The authors classify this study as a clinical experiment, however, it is not a clinical experiment, it is a prospective intervention cohort.
**Methodological quality: Newcastle Ottawa cohort**	
Poor methodological quality.<br>Selection (1 star):<br>1. Representativeness of the exposed cohort: c) Selected group<br>2. Selection of the unexposed cohort: a) From the same community as the exposed cohort (one star)<br>3. Determination of exposure: d) No description<br>4. Demonstration that the outcome of interest was not present at the beginning of the study: b) No<br><br>Comparability (0 stars):<br>c) The cohorts are not comparable according to the design or controlled analysis to detect confounding factors<br><br>Result (3 stars):<br>1. Evaluation of the result: b) Records (1 star)<br>2. Was the follow-up long enough to produce results?: a) Yes (1 star)<br>3. Adequacy of follow-up of the cohorts: b) Subjects lost to follow-up are unlikely to introduce bias: the number of losses was less than or equal to 20% or the description of those lost did not suggest differences with those followed. (1 star)	
**Risk of bias (ROBINS-I)**	

Bias	Assessment	Medium

Confounding bias	Critical risk of bias.	There is a potential for confounding in the effect of the intervention in this study. There are unmeasured variables in both groups that may be unmeasured confounding factors and that may be related to both the intervention chosen by the women and the outcome and not be in the causal chain, such as the presence of comorbidities, which are not reported. . There was no time-varying confounding, as patients did not change between the intervention group and the group not exposed to oral contraceptives. The authors did not perform a statistical analysis adjusting for potential confounding variables. To control for confounding, they did not perform stratification, regressions, matching, standardization, or inverse probabilistic weighting. There was no adjustment of post-intervention variables.<br>The study poses significant difficulties in its ability to provide valuable evidence about the effects of the intervention.
Selection bias	Critical risk of bias.	There was no random selection of participants. The selection of the intervention or comparator group could be related to the characteristics of the women. The start of follow-up is the same between the intervention group and the comparator group. No methods were used to adjust for the presence of selection bias.
Bias in the classification of interventions	Moderate risk of bias.	The intervention and comparator group were clearly defined. The information used to define the two groups was recorded prior to the start of the study. However, the choice of intervention or comparator group may have been affected by knowledge of the outcome or the risk of the outcome.
Bias due to deviations from planned interventions	Serious risk of bias.	There could have been performance bias, which was not reported in the study, however, given that the researchers and women had knowledge of the type of intervention or comparator they were in, they became more susceptible to this type of bias. These deviations could affect the result. The majority of women adhered to the intervention assignment. The presence of co-interventions in the intervention and comparator groups is unknown.
Bias due to missing data	Low risk of bias.	The presence of missing data is not reported. Outcome data are available for all participants who completed the study. Patients were not excluded due to the presence of missing data or due to missing data on the variables necessary for the analysis.

Bias in measuring results	Moderate risk of bias.	The outcome measurement may have been influenced by knowledge of the type of contraceptive received. The outcome evaluators were aware of the type of intervention received by the women. The method used to evaluate the outcomes was the same for both groups. The presence of systematic errors in the measurement of outcomes, related to the type of contraceptive used, is unknown.
Bias in the selection of results reporting	Low risk of bias.	The estimator of the effect is not presented using multiple measures of the outcomes, nor multiple analyzes of the outcomes or in different subgroups.
General bias	Critical risk of bias: the study is problematic in providing useful evidence.	

De Rossi 2014 [91]

Feature	Description
Methods	*Study design*: prospective cohort.<br>*Observation time*: 6 months.<br>*Country*: Brazil.<br>*Research centers*: Family Planning Clinic of the Department of Gynecology and Obstetrics of the Hospital das Clínicas of the Faculty of Medicine of the University of São Paulo.<br>*Language*: English.<br>*Generalities of the study*: The aim of this study was to evaluate the effects of oral contraceptives containing 20µg of ethinyl estradiol and 3 mg of drospirenone in overweight women with mild hypertension on blood pressure, BMI, insulin resistance and lipid parameters. Convenience sampling.
Population	*Sample size*: 65.<br>*Groups*: exposed cohort: n: 40, unexposed cohort: n: 25.<br>*Sociodemographic data*: the sociodemographic characteristics of the exposed group or the non-exposed group are not presented<br>*Inclusion criteria*: women from 18 to 45 years old who attended the Family Planning Clinic of the Department of Gynecology and Obstetrics of the Hospital de Clínicas of the Faculty of Medicine of the University of São Paulo, with grade 1 hypertension, grade I overweight or obesity (BMI of 25 to 29.9 and 30 to 34.9, respectively) and no additional comorbidity. Without having been using hormonal contraceptives in the last six months.<br>*Exclusion criteria*: women with: positive pregnancy test, total cholesterol (TC) > 240 mg/dl, low-density lipoprotein cholesterol (LDL) > 160 mg/dl, high-density lipoprotein cholesterol (HDL) < 40 mg/dl, triglycerides (TG) > 150 mg/dl and fasting glucose ≥ 100 mg/dl.<br>*Definition of high blood pressure*: not described.<br>*Hypertensive women*: 65 (100%).

	Comorbidities of hypertensive women: overweight or obesity grade I. The number of women with each pathology cannot be determined.<br>*Use of other medications in addition to contraceptive methods*: cannot be determined.
Intervention/Comparator	The intervention was combined oral contraceptives with 20 µg of ethinyl estradiol and 3 mg of drospirenone, used for 6 months.<br>The comparator was the use of condom and copper intrauterine device for 6 months.
Outcomes	Worsening of the underlying condition<br>*Daytime SBP: Intervention group: Baseline: 128.22+-1.66 6 months: 124.11 +-1.45 p: 0.197; Control group: Baseline: 130.48+-2.4 6 months: 127.10+-2.59 p:0.20<br>*Daytime DBP: Intervention group: Baseline: 82.51+-1.56 6 months: 81.56+-1.51 p: 0.495; Control group: Baseline: 84.81+-1.86 6 months: 82.52+-2.15 p: 0.28<br>*Nocturnal PAS: Intervention group: Baseline: 114.5+-1.79 6 months: 110.24+.1.77 p: 0.032; Control group: Baseline: 114.02+-2.63 6 months: 113.11+-2.77 p:0.68<br>*Mean nocturnal SBP reduction: Intervention group: Baseline: 13.24+-1.08 6 months: 15.47+-0.82 p:0.027; Control group: Baseline: 16.03+-1.69 6 months: 14.04+-1.5 p:0.30<br>*Nocturnal DBP: Intervention group: Baseline: 68.74+-1.56 6 months: 65.34+-1.74 p: 0.062; Control group: Baseline: 67.82+-2.09 6 months: 69.13+-1.96 p: 0.5<br><br>Increase in body mass<br>*BMI: Intervention group: Baseline: 28.9+-0.78 6 months:28.45+-0.79 p: 0.620; Control group: Baseline: 30.79+-1.20 6 months: 29.01+-1.11 p: 0.27<br><br>Deterioration of metabolic parameters<br>*Total cholesterol: Intervention group: Baseline: 189.6+-7.55 6 months:196.61+-8.79 p:0.879; Control group: Baseline:188.1+-7.9 6 months:186.3+-6.88 p: 0.74<br>*LDL cholesterol: Intervention group: Baseline:108.14+-9.89 6 months:102.05+-9.82 p:0.486; Control group: Baseline: 109.8 6+-7.86 6 months: 110.5+-6.67 p:0.88<br>*HDL cholesterol: Intervention group: Baseline:51.12+-2.25 6 months:58.29+-2.35 p:0.06; Control group: Baseline:58.2+-2.69 6 months:55.6+-2.46 p:0.46<br>*Triglycerides: Intervention group: Baseline:140.00+-18 6 months:160.02+-15.45 p:0.244; Control group: Baseline:100.2+-9.13 6 months:100.6+-10.5 p:0.98
Financing	They don't report.
Declaration of interests	They don't report.
Notes	The authors classify this study as a clinical experiment, however, it is not a clinical experiment, it is a prospective intervention cohort.
**Methodological quality: Newcastle Ottawa cohort**	

Poor methodological quality.<br>Selection: 2 stars<br>1) Representativeness of the exposed cohort: c) Selected group<br>2) Selection of the unexposed cohort: a) From the same community as the exposed cohort (one star)<br>3) Determination of exposure: d) No description<br>4) Demonstration that the outcome of interest was not present at the beginning of the study: a) Yes (one star)<br><br>Comparability:<br>c) The cohorts are not comparable based on the design or analysis controlled for confounding factors: we do not have information on the comparability of the cohorts.<br><br>Result: 2 stars<br>1) Evaluation of the result: b) Records (one star)<br>2) Was the follow-up long enough to produce results?: a) Yes (one star)<br>Please indicate the average duration of follow-up and a brief justification for the previous evaluation: 6 months<br>3) Adequacy of cohort follow-up: d) No declaration

**Risk of bias (ROBINS-I)**		
Bias	Assessment	Medium
Confounding bias	Critical risk of bias.	There is a possibility of confounding the effect of the intervention. There is unmeasured confusion, the study does not report the presence of conditions that could be related to the outcomes. The authors did not perform statistical analyzes to adjust for possible confounding present in the study. There is no time-varying confounding, participants do not change between the intervention group and the comparator group, and the analysis was not based on dividing the women's follow-up time according to the intervention received. There was no presence of control of post-intervention variables.<br>The study has significant difficulties in terms of its ability to provide useful evidence about the effects of the intervention.
Selection bias	Critical risk of bias.	There was no random assignment to the intervention groups or the comparator group. The characteristics of the participating women could have affected the choice of contraceptive method. The start of follow-up of the women in the intervention group and the comparator group coincided. No techniques are used to adjust the risk of selection bias.<br>The study has difficulties in providing useful evidence on the effects of the intervention.
Bias in the classification of interventions	Moderate risk of bias.	The intervention and comparator groups were clearly defined. The information used to define the intervention and comparator groups was defined at

		the beginning of the study. Allocation to each group may have been affected by knowledge of the outcome or risk of the outcome.
Bias due to deviations from planned interventions	Serious risk of bias.	There could have been bias due to deviations in the interventions, since the researchers and the participating women were aware of the group to which each woman belonged. These deviations could affect the result. The presence of co-interventions in the groups is unknown. Women adhered to the intervention assignment.
Bias due to missing data	Low risk of bias.	The authors do not report the presence of missing data; they indicate that they performed the outcome measures on all participating women. According to the authors, the results of the outcomes are found for all participants. Patients were not discarded due to missing data or lack of information on the variables required for the analysis.
Bias in measuring results	Moderate risk of bias.	The measurement of the outcome could be affected by knowledge of the type of contraceptive received by the women. The evaluators of the outcomes were aware of the group to which each participant belonged. The method used to evaluate the outcomes was the same for both groups. It cannot be determined whether there were systematic errors in the measurement of the outcomes.
Bias in the selection of results reporting	Low risk of bias.	The effect estimator is not presented across multiple outcome measures, multiple outcome analyzes are not performed, nor are different subgroups presented.
General bias	Critical risk of bias: the study poses difficulties in providing useful evidence.	

Case series studies:

Elkik 1986 [93]

Feature	Description
Methods	*Study design*: prospective case series.<br>*Data collection dates*: they do not describe.<br>*Country*: USA.<br>*Research centers*: they don't report it.<br>*Language*: English.

	Generalities of the study: The objective of the study was to describe the safety of the vaginal ring in hypertensive women by measuring changes in blood pressure and plasma renin substrate concentration.
Participants	*Sample size*: 12, however, the study was completed by 9 women.<br>*Sociodemographic data*: mean (SD) age: 29.17 (6.84) years, years with arterial hypertension: median (IQR): 3 (5.5) years, median (IQR) weight: 60.5 (9) Kg.<br>*Inclusion criteria*: hypertensive women referred to the health center who use vaginal rings.<br>*Exclusion criteria*: women with secondary arterial hypertension.<br>*Definition of high blood pressure*: blood pressure was measured during 2 cycles prior to treatment for 5 times, then during the first, second, fourth, sixth and ninth to twelfth cycles, during the use of the vaginal ring and after a recovery period of one month .<br>*Hypertensive women*: 12 (100%).<br>*Comorbidities of hypertensive women*: 2/12 women were moderate smokers (<10 cigarettes/day). They all weighed +-15% of their ideal weight.<br>*Use of other medications in addition to contraceptive methods*: The antihypertensive medications used by 9/12 of the women in the study are: acebutolol, spirolactone, amiloride, hydrochlorothiazide, triamterene, cyclothiazide, metoprolol and clonidine.
Exposure	The exposure was the combined vaginal ring (estradiol and levonorgestrel). The vaginal ring was used for a mean (SD) of 15.6 (6.7) menstrual cycles in 12 hypertensive women, with a range of 6 to 24 cycles.<br>The vaginal ring released a mean (SD) of 293 (54) µg/day of levonorgestrel and 183 (34) µg/day of estradiol.
Outcomes	Unwanted pregnancies: no unwanted pregnancies occurred in the 187 cycles observed.<br>Systolic and diastolic blood pressure values:<br>Control cycles: cycle -1: Number of patients: 12: SBP (mmHg)+-SD: 139.7+-15.7; DBP (mmHg)+SD: 88.5+-15.7<br>Control cycles: cycle 0: Number of patients: 12: SBP (mmHg)+-SD: 134.7+-18.4; DBP (mmHg)+SD: 82.4+-11.2<br>Treatment cycles: cycle 1: Number of patients: 12: SBP (mmHg)+-SD: 136.1+-14.6; DBP (mmHg)+SD: 85.0+-9.3<br>Treatment cycles: cycle 2: Number of patients: 12: SBP (mmHg)+-SD: 134.1+-18.3; DBP (mmHg)+SD: 82.8+-12.5<br>Treatment cycles: cycle 4: Number of patients: 12: SBP (mmHg)+-SD: 130.1+-12.7; DBP (mmHg)+SD: 81.4+-17.7<br>Treatment cycles: cycle 6: Number of patients: 12: SBP (mmHg)+-SD: 136.0+-16.3; DBP (mmHg)+SD: 83.4+-9.7<br>Treatment cycles: cycle 9-12: Number of patients: 11; SBP (mmHg)+-SD: 133.6+-16.1: DBP (mmHg)+-SD: 82.8+-10.7<br>Recovery cycles 1-2: Number of patients: 11: SBP (mmHg)+-SD: 132.5+-15.6; DBP (mmHg)+SD: 86.9+-13.02<br>No significant differences were found in SBP or DBP compared to pre- or post-treatment values.<br><br>Total cholesterol: mean total cholesterol (DS) mg/100mL: control (month 0): 182+-30; 1 month: 158+-30; 2 months: 155+-35; 4 months: 161+-27; 6

	months: 152+-34; 9-12 months: 157+-26 (p<0.01: measurements compared to month 0).<br><br>LDL cholesterol: mean LDL cholesterol (DS) mg/100mL: control (month 0): 114+-28; 1 month: 107+-26; 2 months: 105+30; 4 months: 101+-19; 6 months: 105+-27; 9-12 months: 109+-32 (p: not significant compared to measurements with month 0 (p value not reported).<br><br>HDL cholesterol: mean HDL cholesterol (DS) mg/100mL: Control (month 0): 59+-10; 1 month: 42+-9; 2 months: 41+-6; 4 months: 40+-6; 6 months: 38+-9; 9-12 months: 38+-3 (p<0.001 compared measurements with month 0).<br><br>Triglycerides: mean triglycerides (DS) mg/100mL: Control (month 0): 52+-17; 1 month: 38+-8; 2 months: 38+-13; 4 months: 41+-15; 6 months: 41+-10; 9-12 months: 38+-11 (p<0.05 compared to measurements with month 0)).<br><br>Abandonment of the contraceptive method due to side effects: 1 patient was excluded after 10 cycles due to cervical ulceration, which disappeared spontaneously.<br>Adverse effects of the interventions: the mean (SD) days of spotting or premature bleeding was 0.7 (1) days/cycle. 3 of 12 women had increased vaginal discharge. Endometrial biopsies performed on 6 women after using the vaginal ring for 6-9 months showed atrophic changes due to exposure to progestogens; the presence of hyperplasia, carcinoma or sarcoma was not identified.
Financing	Este estudio fue financiado por U.S. Agency for International Development, International Development Research Centre of Canada, Ford Foundation, Rockefeller Foundation y George J. Hecht Fund.
Declaration of interests	They don't report.
Notes	Series of exposed participants.<br>Before starting the use of the vaginal ring, blood pressure was measured on 2 different days one month apart, and physical and gynecological examinations were performed. All patients were examined by the same doctor.
**Critical Approach: Joanna Briggs Institute**	
Unclear critical assessment<br>- Were there clear criteria for inclusion in the case series? No<br>- Was the disease measured in a standard and reliable way in all participants included in the case series? Yeah<br>- Were valid methods used to identify the disease in all participants included in the case series? It is not clear<br>- Was there a consecutive inclusion of participants in the case series? No<br>- Did the case series include all participants? No<br>- Were the demographic data of the study participants clearly reported? Yeah<br>- Was the clinical information of the participants clearly reported? Yeah<br>- Were the results or outcomes of case follow-up clearly communicated? Yeah<br>- Was the demographic information of the centers or clinics that presented the cases clearly communicated? No<br>- Was the statistical analysis adequate? Yeah	

Bounhoure 2008 [92]

**Feature**	**Description**
Methods	*Study design*: series of cases.<br>*Data collection dates*: 1977-2006.<br>*Country*: France.<br>*Research centers*: they do not report.<br>*Language*: French.<br>*Generalities of the study*: The objective of this study was to report 12 cases of acute myocardial infarction in women between 30 and 40 years old who were using different types of oral contraceptives.
Participants	*Sample size*: 12.<br>*Sociodemographic data*: The hypertensive women were 30, 34 and 36 years old, respectively.<br>*Inclusion criteria*: women between 30 and 40 years old, users of hormonal contraception, hospitalized between 1977 and 2006 due to acute myocardial infarction.<br>*Exclusion criteria*: does not report.<br>*Definition of high blood pressure*: they do not report.<br>*Hypertensive women*: 3 (25%).<br>*Comorbidities of hypertensive women*: 2/3 hypertensive women were obese. 3/3 hypertensive women were active smokers. 1/3 hypertensive women had a history of thrombosis.<br>*Use of other medications in addition to contraceptive methods*: cannot be determined.
Exposure	The exposure was combined oral contraceptives.<br>Of hypertensive women:<br>- 2 patients used first generation contraceptives with doses of ethinyl estradiol of 100-150µg together with a northeastern steroid or norgestrel.<br>- 1 patient used second generation contraceptives with ethinyl estradiol doses of 50µg combined with levonorgestrel or norgestrel.
Outcomes	Acute myocardial infarction. All three women had acute myocardial infarction.<br>*Definition of the outcome*: Acute myocardial infarction was confirmed through the use of electrocardiogram and cardiac enzymes.
Financing	They don't report.
Declaration of interests	They don't report.
Notes	
**Critical Approach: Joanna Briggs Institute**	
Unclear critical assessment<br>- Were there clear criteria for inclusion in the case series? No<br>- Was the disease measured in a standard and reliable way in all participants included in the case series? No<br>- Were valid methods used to identify the disease in all participants included in the case series? No<br>- Was there a consecutive inclusion of participants in the case series? No	

- Did the case series include all participants? It is not clear
- Were the demographic data of the study participants clearly reported? Yeah
- Was the clinical information of the participants clearly reported? Yeah
- Were the results or outcomes of case follow-up clearly communicated? Yeah
- Was the demographic information of the centers or clinics that presented the cases clearly communicated? No
- Was the statistical analysis adequate? Yeah
