# Supplementary material for: Safety and effectiveness of hormonal vs non-hormonal or no contraception in women with hypertension and future fertility desire: A broad-scope systematic review
Source: PLoS One. 2026 Mar 31;21(3):e0345959. doi: 10.1371/journal.pone.0345959 (PMC13038026; doi:10.1371/journal.pone.0345959)
Supplement: S12 Appendix — (PDF) [file pone.0345959.s012.pdf]

L. Appendix S12: General characteristics of the exposures/interventions and study comparators

Study	Type of hormonal contraceptive evaluated	Exposure	Comparator	Definition of current use of hormonal contraceptives	Definition of no current use of hormonal contraceptives	Estrogen and progestogen doses
Collaborative Group for the Study of Stroke in Young Women 1975 [84]	Combined oral contraceptive	Current use	No current use	During the calendar month of admission to the hospital or prior to the outcome (hospital cases and controls) or the index date of the case (community controls).	Does not present definition.	They do not describe
Lidegaard 1993 and 1995 [86,87]	Combined oral contraceptive or progestin-only pill	Current use	No current use	Use of contraceptives at the time of hospital admission.	Previous use or never having used the hormonal contraceptive method.	Progestin-only pills; 50 ug of estrogen ("medium estrogen" pills); 30-40 ug of estrogen ("miniestrogens"); 20 g of estrogen ("microestrogen" pills).
Croft 1989 [77] and	Combined oral contraceptive or progestin-only pill	Current use	No current use	Does not present definition.	Previous use or never having used the hormonal	Progestogen content: Norethindrone acetate (norethisterone) (4 mg+50 ug EE[1]; 3mg+50ug EE[1];

Study	Type of hormonal contraceptive evaluated	Exposure	Comparator	Definition of current use of hormonal contraceptives	Definition of no current use of hormonal contraceptives	Estrogen and progestogen doses
Hannaford 1994 [78]					contraceptive method.	1mg+50ug EE[1]); Other combinations (Lynestrenol (lynestrol); Ethynodiol diacetate; Levonorgestrel; Other progestins). Estrogen content: Mestranol or ethinyl estradiol (>50 ug; 50 ug; <50 ug)
Tanis 2001 [83], Kemmeren 2002 [89], Van Den Bosch 2003 [81]	Combined oral contraceptive or progestin-only pill	Current use	No current use	Contraceptive use within the month prior to the index date.	Previous use or never having used the hormonal contraceptive method.	First-generation oral contraceptives, which contain linestrenol or norethindrone; Second-generation oral contraceptives, which contain norgestrel or levonorgestrel; Third generation oral contraceptives, which contain desogestrel or Gestodene; Oral contraceptives that contain an estrogen and other types of progestins (cyproterone or norgestimate); Progestogen only. Concentrations: 50ug of ethinyl estradiol and 125ug of levonorgestrel; 30ug of ethinyl estradiol and 150ug of levonorgestrel

Study	Type of hormonal contraceptive evaluated	Exposure	Comparator	Definition of current use of hormonal contraceptives	Definition of no current use of hormonal contraceptives	Estrogen and progestogen doses
Heinemann 1998 [76]	Combined oral contraceptive	Current use	No current use	Contraceptive use within 3 months prior to the index date of the study condition or 3 months prior to hospitalization in hospital controls or the day of the interview in community controls.	Previous use or never having used the hormonal contraceptive method.	First generation oral contraceptives (high doses of ethinyl estradiol 50 µg); Second generation oral contraceptives (low dose of ethinyl estradiol with other progestins other than Gestodene and desogestrel; Third generation oral contraceptives (low doses of ethinyl estradiol < 50 µg] with Gestodene or desogestrel).
WHO 1995 [79], WHO 1996a [85], WHO 1996b [88], WHO 1997 [82]	Combined oral contraceptive	Current use	No current use	Contraceptive use within 3 months prior to the index date of the study condition or admission to hospitalization of controls.	Previous use or never having used the hormonal contraceptive method.	First generation oral contraceptives, containing ethinodiol diacetate, linoestrenol, norethisterone, norethisterone acetate and norethynodrel.<br>Second generation oral contraceptives, which contain norgestrel, levonorgestrel and norgestnenone.<br>Third generation oral contraceptives, which contain desogestrel, tristedene and norgestimate.<br>Others, containing chlormadinone acetate and cyproterone acetate.

Study	Type of hormonal contraceptive evaluated	Exposure	Comparator	Definition of current use of hormonal contraceptives	Definition of no current use of hormonal contraceptives	Estrogen and progestogen doses
						Estrogen dosage: < 50ug, ≥50ug.
WHO 1998 [80]	Combined injectable contraceptive, progestin-only pill, and progestin-only injectables	Current use	No current use	Contraceptive use within 3 months prior to the index date of the study condition or admission to hospitalization of controls.	Previous use or never having used the hormonal contraceptive method.	Types of oral progestagens: Continuous (d-Norgestrel 0.03 mg, dl-norgestrel 0.075 mg, ethinediol diacetate 0.5 mg, lynestrenol 0.5 mg, noretisterone 0.35 mg), postcoital (d-norgestrel 0.75 mg, anorethidrate dipropionate 7.5 mg. dl-norgestrel 3.0 mg, noretisterone 5.0 mg). Types of injectable contraceptives: progestin-only (medroxyprogesterone acetate 150 mg, norethisterone enanthate 200 mg), combined (alfasone acetophenide 120 mg + estradiol enanthate 10 mg, dihydroxyprogesterone acetophenide 150 mg + estradiol enanthate 10 mg, caproate hydroxyprogesterone 250 mg + estradiol valerate 5

Study	Type of hormonal contraceptive evaluated	Exposure	Comparator	Definition of current use of hormonal contraceptives	Definition of no current use of hormonal contraceptives	Estrogen and progestogen doses
						mg, megestrol acetate 25 mg + estradiol 3.5 mg).
de Morais 2014 [90]	Combined oral contraceptive	Use of combined oral contraceptives	Use of condom or copper intrauterine device	[2]	[2]	[2]
by Rossi 2014 [91]	Combined oral contraceptive	Use of combined oral contraceptives	Use of condom or copper intrauterine device	[2]	[2]	[2]
Elkik 1986 [93]	Combined contraceptive vaginal ring	Use of combined vaginal ring	[2]	[2]	[2]	[2]
Bounhoure 2008 [92]	Combined oral contraceptive	Use of combined oral contraceptives	[2]	[2]	[2]	[2]

[1]EE: ethinyl estradiol

[2] It corresponds to sections that do not apply to the study.
