# Supplementary material for: Safety and effectiveness of hormonal vs non-hormonal or no contraception in women with hypertension and future fertility desire: A broad-scope systematic review
Source: PLoS One. 2026 Mar 31;21(3):e0345959. doi: 10.1371/journal.pone.0345959 (PMC13038026; doi:10.1371/journal.pone.0345959)
Supplement: S13 Appendix — (PDF) [file pone.0345959.s013.pdf]

M. Appendix S13: Summary table of results of included GRADE studies

Should combined oral contraceptives be used compared to not used combined oral contraceptives in women of childbearing age with high blood pressure?
Combined oral contraceptives compared to not using combined oral contraceptives in women of childbearing age with high blood pressure
Settings: Family planning

**Certainty assessment**							**Number of patients**		**Effect**			
**Number of studies**	**Study design**	**Risk of bias**	**Inconsistency**	**Indirect evidence**	**Imprecision**	**Other considerations**	**Combined oral contraceptives**	**Do not use combined oral contraceptives**	**Relative (95% CI)**	**Absolute (95% CI)**	**Certainty**	**Importance**
**Ischemic cerebrovascular event (assessed with: Women who presented this outcome)**												
3[1,2,3]	observational studies	very serious [a]	very serious [b]	it's not serious	very serious [c]	none	Of the three studies, one found that the odds of experiencing ischemic cerebrovascular events were higher among "current users" of combined oral contraceptives than among "non-current users." In contrast, the other two studies suggested that "current" use of combined oral contraceptives could be both positively and negatively associated with the presence of ischemic cerebrovascular events.				⨁◯◯◯ Very low	CRITICAL
**Hemorrhagic cerebrovascular event (assessed with: Women who developed this outcome)**												

Certainty assessment							Number of patients		Effect		Certainty	Importance
**Number of studies**	**Study design**	**Risk of bias**	**Inconsistency**	**Indirect evidence**	**Imprecision**	**Other considerations**	**Combined oral contraceptives**	**Do not use combined oral contraceptives**	**Relative (95% CI)**	**Absolute (95% CI)**		
2[1,4]	observational studies	very serious [a]	it's not serious	it's not serious	it's not serious[d]	none	494 cases 345 Controls 79/39 exposed 415/306 not exposed		**OR 1.64** (1.08 a 2.50)	-	⊕⊕○○ Low	CRITICAL
							-	15.0%		74 more per 1,000 (from 10 more to 156 more)		
**Increase in body mass index (assessed with: Increase in BMI)**												
2[5,6]	observational studies	extremely serious [e]	serious[f]	it's not serious	very serious [g]	none	In the Morais 2014 study, a decrease in BMI was found 6 months after starting oral contraceptives in users of this contraceptive method and no differences were found in BMI 6 months after starting the study in non-users of oral contraceptives. oral contraceptives. In the study by Rossi 2014, they found no differences in BMI values in women who used oral contraceptives or in women who did not use oral contraceptives.				⊕○○○ Very low	IMPORTANT
**Deterioration of metabolic parameters: serum total cholesterol values (assessed with: Changes in serum concentrations of total serum cholesterol)**												

Certainty assessment							Number of patients		Effect		Certainty	Importance
Number of studies	Study design	Risk of bias	Inconsistency	Indirect evidence	Imprecision	Other considerations	Combined oral contraceptives	Do not use combined oral contraceptives	Relative (95% CI)	Absolute (95% CI)		
2[5,6]	observational studies	extremely serious [e]	it's not serious	it's not serious	very serious [g]	none	In de Morais 2014 and de Rossi 2014, no significant changes in serum total cholesterol levels were identified in women who used oral contraceptives or in women who did not use oral contraceptives at 6 months into the study.				⨁◯◯◯ Very low	IMPORTANT
**Deterioration of metabolic parameters: LDL cholesterol values (assessed with: Changes in serum LDL cholesterol concentrations)**												
2[5,6]	observational studies	extremely serious [e]	it's not serious	it's not serious	very serious [g]	none	In the studies by Morais 2014 and Rossi 2014, no differences were found in serum LDL cholesterol levels 6 months after starting the study in women who used and did not use oral contraceptives.				⨁◯◯◯ Very low	IMPORTANT
**Deterioration of metabolic parameters: HDL cholesterol values (assessed with: Changes in serum HDL cholesterol concentrations)**												
2[5,6]	observational studies	extremely serious [e]	it's not serious	it's not serious	very serious [g]	none	In the studies by Morais 2014 and Rossi 2014, no differences were found in serum HDL cholesterol levels 6 months after starting the study in women who used and did not use oral contraceptives.				⨁◯◯◯ Very low	IMPORTANT
**Deterioration of metabolic parameters: triglyceride values (assessed with: Changes in serum concentrations of triglycerides)**												
2[5,6]	observational studies	extremely serious [e]	it's not serious	it's not serious	very serious [g]	none	In the studies by Morais 2014 and Rossi 2014, no differences were found in serum triglyceride levels 6 months after starting the study in women who used and did not use oral contraceptives.				⨁◯◯◯ Very low	IMPORTANT

CI: Confidence interval; OR: Odds ratio

Explanations

a. The quality of the evidence is degraded by two levels, given that the studies present a high risk of bias. Confounding bias (they do not present measures of association adjusted for possible confounding variables and outcomes). Regarding the risk of measurement bias, there is a high risk of recall bias, since the exposures were collected through surveys with the patients. This poses a potential risk of differential misclassification, given that cases are more likely to recall exposures compared to controls.

b. The quality of the evidence is degraded by two levels due to the presence of unexplained heterogeneity. It is not possible to identify the source of heterogeneity between studies.

c. The quality of the evidence is degraded by two levels given that the confidence intervals of the combined estimator cross the line of no effect and the optimal size of information is 846 patients and there are 613 patients among the three studies. For Collaborative 1975 the optimal information size was 92 patients and has 244 women, in WHO 1996b the optimal information size was 1624 patients and has 304 women, in Heinemann the optimal information size was 470 patients and has 65 women.

d. The optimal information size is 978 patients and there are 839 patients. The confidence interval of the combined estimator does not cross the line of no effect.

e. The quality of the evidence is degraded by three levels since both studies present a critical risk of bias (assessed by ROBINS-I). It has critical confounding and selection bias, serious risk of bias due to deviations from the intended interventions, moderate risk of bias in the classification of interventions and in the measurement of outcomes, and low risk of bias due to missing data and selection of report of the results.

f. The quality of the evidence is degraded at one level, given that in one study they report the presence of a decrease in BMI in the intervention group 6 months after starting the study and in the other study no changes were found, however, In neither study did they adjust for possible confounding variables. It is not possible to identify the possible causes that cause the outcomes to vary between studies.

g. The quality of the evidence is degraded by two levels, given that it is not possible to calculate the optimal size of information with the data that the authors provide in their publications, which suggests precision problems.

References

1.Women, Collaborative,Group,for,the,Study,of,Stroke,in,Young. Oral Contraceptives and Stroke in Young Women: Associated Risk Factors. JAMA; 1975.

2.Organization, World,Health. Ischaemic stroke and combined oral contraceptives: results of an international, multicentre, case-control study. Lancet; 1996.

3.Heinemann LA, Lewis MA,Spitzer WO,Thorogood M,Guggenmoos-Holzmann I,Bruppacher R. Thromboembolic stroke in young women. A European case-control study on oral contraceptives. Contraception; 1998.

4.Organization, World,Health. Haemorrhagic stroke, overall stroke risk, and combined oral contraceptives: results of an international, multicentre, case-control study. Lancet; 1996.

5.de Rossi P, Giribela CRG,Baracat EC,Colombo FMC,Melo NR. Evaluation of blood pressure, body mass index, lipid profile and insulin resistance in mild hypertensive overweight women with the use of a low dose combined oral contraceptive containing drospirenone: Results from a prospective clinical trial. G Ital Ostet E Ginecol; 2014.

6.de Morais TL, Giribela C,Nisenbaum MG,Guerra G,Mello N,Baracat E,et al. Effects of a contraceptive containing drospirenone and ethinylestradiol on blood pressure, metabolic profile and neurohumoral axis in hypertensive women at reproductive age. Eur J Obstet Gynecol Reprod Biol; 2014.

Should combined oral contraceptives be used compared to not used combined oral contraceptives in women of childbearing age with high blood pressure?

Combined oral contraceptives or progestin-only pills compared with not using combined oral contraceptives or progestin-only pills in women of childbearing age with high blood pressure

Configuration: Family planning

**Certainty assessment**							**number of patients**		**Effect**			
**Number of studies**	**Study design**	**Risk of bias**	**Inconsistency**	**Indirect evidence**	**Imprecision**	**Other considerations**	**combined oral contraceptives or progestin-only pills**	**do not use combined oral contraceptives or progestin-only pills**	**Relative (95% CI)**	**Absolute (95% CI)**	**Certainty**	**Importance**
**Ischemic cerebrovascular event (assessed with: Number of women who presented this outcome)**												
2[1,2,3]	observational studies	very serious [a]	serious[b]	it's not serious	very serious [c]	none	In Kemmeren 2002, the crude OR of ischemic cerebrovascular event among 'current' users of combined oral contraceptives or progestin-only pills was 0.95 (95% CI 0.42-22.15) and in Lidegaard 1993 and 1995 of 4.68 (95% CI 1.48-14.79).				⊕◯◯◯ Very low	CRITICAL
**Acute myocardial infarction (assessed with: Number of women who presented this outcome)**												
2[4,5]	observational studies	very serious [a]	it's not serious	it's not serious	very serious [d]	none	98 cases 69 Controls 29/27 exposed 69/82 not exposed		**OR 1.15** (0.60 a 2.19)	-	⊕◯◯◯ Very low	CRITICAL
							-	0.0%		0 minus per 1,000 (from 0 minus to 0 minus)		

CI: Confidence interval; OR: Odds ratio

Explanations

a. The quality of the evidence is degraded by two levels, given that the two studies present a high risk of bias. It presents confusion bias (they do not present association measures adjusted for the possible confounding variables and the outcome) and measurement bias, given the high risk of memory bias, since the exposures were obtained through surveys, increasing the risk of differential misclassification. , since cases have a greater probability of remembering the exposures compared to controls.

b. It is uncertain whether the heterogeneity present is due to the definition that each study used for “current use” exposure.

c. In Kemmeren 2002, the quality of the evidence is degraded by two levels because the confidence interval crosses the line of no effect and the optimal information size is 53,240 and includes 103 women. In Lidegaard 1993 and 1995 it is degraded by one level because the confidence interval is wide and the optimal information size is 116 and has 124 women.

d. The quality of the evidence is degraded at two levels, given that the optimal size of information is 8354 patients and there are a total of 207 patients; on the other hand, the confidence interval crosses the line of no effect.

References

1. Kemmeren JM, Tanis BC, van den Bosch MAAJ, Bollen ELEM, Helmerhorst FM, van der Graaf Y, et al. Risk of Arterial Thrombosis in Relation to Oral Contraceptives (RATIO) study: oral contraceptives and the risk of ischemic stroke. strokes; 2002.

2.O, Lidegaard. Oral contraceptives, pregnancy and the risk of cerebral thromboembolism: the influence of diabetes, hypertension, migraine and previous thrombotic disease. Br J Obstet Gynaecol; 1995.

3.O, Lidegaard. Oral contraception and risk of a cerebral thromboembolic attack: results of a case-control study. BMJ; 1993.

4.Croft P, Hannaford PC. Risk factors for acute myocardial infarction in women: evidence from the Royal College of General Practitioners’ oral contraception study.. BMJ; 1989.

5. Tanis BC, van den Bosch MA, Kemmeren JM, Cats VM, Helmerhorst FM, Algra A, et al. Oral contraceptives and the risk of myocardial infarction. N Engl J Med; 2001.
