# Supplementary material for: Safety and effectiveness of hormonal vs non-hormonal or no contraception in women with hypertension and future fertility desire: A broad-scope systematic review
Source: PLoS One. 2026 Mar 31;21(3):e0345959. doi: 10.1371/journal.pone.0345959 (PMC13038026; doi:10.1371/journal.pone.0345959)
Supplement: S14 Appendix — (PDF) [file pone.0345959.s014.pdf]

N. Appendix S14: Evidence quality assessment table for individual studies

Should combined oral contraceptives be used compared to no combined oral contraceptives in women of childbearing age with high blood pressure?											
**Unwanted pregnancies**											
Number of studies	Study design	Follow-up duration	Risk of bias	Indirect evidence	Imprecision	Publication bias	Big effect	Plausible publication factors	Dose-response gradient	Importance	Certainty
1	Cohort study (de Morais 2014)	6 months	Extremely serious risk of bias[A]	No[B]	Very serious[C]	No	No	No	No	9. Critical	Very low
**Acute myocardial infarction**											
1	Case-control study (WHO 1997)	Data collection dates: February 1, 1989 to January 31, 1995.	Very serious risk of bias[D]	No[B]	Very serious[E]	No	No	No	No	9. Critical	Very low
1	Case series (Bounhoure 2008)	Data collection dates: 1977-2006.	Very serious risk of bias[F]	No	Not applicable	No	No	No	No	9. Critical	Very low
**Venous thromboembolic events**											
1	Case-control study (WHO 1995)	Data collection dates: February 1, 1989 to January 31, 1993.	Very serious risk of bias[D]	No[B]	Very serious[C]	No	No	No	No	9. Critical	Very low

**Worsening of baseline condition: changes in SBP (cohorts)**											
1	Cohort study (de Morais 2014)	6 months	Extremely serious risk of bias[A]	No[B]	Very serious[G]	No	No	No	No	9. Critical	Very low
**Worsening of baseline condition: changes in DBP (cohorts)**											
1	Cohort study (de Morais 2014)	6 months	Extremely serious risk of bias[A]	No[B]	Very serious[G]	No	No	No	No	9. Critical	Very low
**Worsening of baseline condition: changes in daytime SBP (cohorts)**											
1	Cohort study (de Rossi 2014)	6 months	Extremely serious risk of bias[A]	No[B]	Very serious[G]	No	No	No	No	9. Critical	Very low
**Worsening of baseline condition: changes in nocturnal SBP (cohorts)**											
1	Cohort study (de Rossi 2014)	6 months	Extremely serious risk of bias[A]	No[B]	Very serious[G]	No	No	No	No	9. Critical	Very low
**Worsening of baseline condition: changes in daytime DBP (cohorts)**											
1	Cohort study (de Rossi 2014)	6 months	Extremely serious risk of bias[A]	No[B]	Very serious[G]	No	No	No	No	9. Critical	Very low
**Worsening of baseline condition: changes in nocturnal DBP (cohorts)**											
1	Cohort study (de Rossi 2014)	6 months	Extremely serious risk of bias[A]	No[B]	Very serious[G]	No	No	No	No	9. Critical	Very low
**Increase abdominal perimeter**											

1	Cohort study (de Morais 2014)	6 months	Extremely serious risk of bias[A]	No[B]	Very serious[G]	No	No	No	No	4. Important	Very low
**Alteration of kidney function tests**											
1	Cohort study (de Morais 2014)	6 months	Extremely serious risk of bias[A]	No[B]	Very serious[G]	No	No	No	No	4. Important	Very low

[A] It presents a critical risk of bias, using the ROBINS-I tool, it presents: critical risk of confounding bias (the results are not adjusted to the possible confounding variables, there is a risk of the presence of residual confusion) and selection bias (the selection of the group intervention or comparator could be related to the characteristics of the women). Serious risk of bias due to deviations from the intended interventions (since the researchers and women had knowledge of the type of intervention or comparator they were in, they become more susceptible to this type of bias). Moderate risk of bias in the classification of interventions (the choice of intervention or comparator group may have been affected by knowledge of the outcome or the risk of the outcome) and in the measurement of outcomes (measurement of the outcome may be influenced by knowledge of the type of contraceptive received). Low risk of bias due to missing data and selection of reporting of results.

[B] It does not present sources of indirect evidence (differences in the population (applicability), differences in the intervention (applicability), differences in the outcomes measured (alternative outcomes) or indirect comparisons).

[C] It presents precision problems because it is not possible to calculate the optimal size of information with the data provided by the authors.

[D] The quality of the evidence is degraded by two levels, given that although the methodological quality of this study is high, it presents a high risk of bias. It presents confusion bias (they do not present association measures adjusted for possible confounding variables and outcomes) and measurement bias, given the high risk of memory bias, since the exposures were obtained through interviews, increasing the risk of differential misclassification. , since cases have a greater probability of remembering the exposures compared to controls.

[E] The quality of the evidence is degraded by two levels, given that the optimal size of information is 226 patients and there are a total of 175 patients; additionally, the confidence interval is wide. However, the confidence interval does not cross the line of no effect.

[F] As this is a case series, it presents a high risk of bias. It presents a high risk of selection bias (only exposed patients are presented, the selection of patients is for convenience), measurement bias (the way exposure was measured is unknown) and confusion (the outcomes are not evaluated when controlling for potential variables). of confusion).

[G] It is not possible to calculate the optimal size of information since the authors do not provide the data corresponding to the differences in means between groups (users versus non-users), given the above, neither are the corresponding dispersion measures counted nor can they be calculated, since they do not There is neither a p value, nor a t statistic, nor confidence intervals of the intergroup mean difference to calculate the standard deviation, nor is there a standard error value for this purpose.

Should the combined contraceptive vaginal ring be used compared to not using the combined contraceptive vaginal ring in women of childbearing age with high blood pressure?											
**Worsening of the underlying condition**											
Number of studies	Study design	Follow-up duration	Risk of bias	Indirect evidence	Imprecision	Publication bias	Big effect	Plausible publication factors	Dose-response gradient	Importance	Certainty
1	Case series (Elkik 1986)	12 months	Very serious risk of bias[A]	No	Not applicable	No	No	No	No	9. Critical	Very low
**Alteration in total cholesterol levels**											
Number of studies	Study design	Follow-up duration	Risk of bias	Indirect evidence	Imprecision	Publication bias	Big effect	Plausible publication factors	Dose-response gradient	Importance	Certainty
1	Case series (Elkik 1986)	12 months	Very serious risk of bias[A]	No	Not applicable	No	No	No	No	9. Critical	Very low
**Alteration of LDL cholesterol levels**											
Number of studies	Study design	Follow-up duration	Risk of bias	Indirect evidence	Imprecision	Publication bias	Big effect	Plausible publication factors	Dose-response gradient	Importance	Certainty
1	Case series (Elkik 1986)	12 months	Very serious risk of bias[A]	No	Not applicable	No	No	No	No	9. Critical	Very low
**Alteration in HDL cholesterol levels**											

Number of studies	Study design	Follow-up duration	Risk of bias	Indirect evidence	Imprecision	Publication bias	Big effect	Plausible publication factors	Dose-response gradient	Importance	Certainty
1	Case series (Elkik 1986)	12 months	Very serious risk of bias[A]	No	Not applicable	No	No	No	No	9. Critical	Very low
**Alteration triglyceride levels**											
Number of studies	Study design	Follow-up duration	Risk of bias	Indirect evidence	Imprecision	Publication bias	Big effect	Plausible publication factors	Dose-response gradient	Importance	Certainty
1	Case series (Elkik 1986)	12 months	Very serious risk of bias[A]	No	Not applicable	No	No	No	No	9. Critical	Very low
[A]It presents a high risk of selection bias (only exposed patients are presented, patient selection is for convenience) and confusion (statistical or methodological methods are not used to control for potential confounding variables).											

**Should combined injectable contraceptives be used compared to not using combined injectable contraceptives in women of childbearing age with high blood pressure?**											
**Ischemic and hemorrhagic cerebrovascular event**											
Number of studies	Study design	Follow-up duration	Risk of bias	Indirect evidence	Imprecision	Publication bias	Big effect	Plausible publication factors	Dose-response gradient	Importance	Certainty
1	Case-control study (WHO 1998)	Dates of data collection: February 1, 1989 to January 31, 1993 (in some	Very serious risk of bias[A]	No[B]	Extremely serious[C]	No	No	No	No	9. Critical	Very low

		cases until January 31, 1995).									
**Acute myocardial infarction**											
Number of studies	Study design	Follow-up duration	Risk of bias	Indirect evidence	Imprecision	Publication bias	Big effect	Plausible publication factors	Dose-response gradient	Importance	Certainty
1	Case-control study (WHO 1998)	Dates of data collection: February 1, 1989 to January 31, 1993 (in some cases until January 31, 1995).	Very serious risk of bias[A]	No[B]	Extremely serious[D]	No	No	No	No	9. Critical	Very low
**Venous thromboembolism**											
Number of studies	Study design	Follow-up duration	Risk of bias	Indirect evidence	Imprecision	Publication bias	Big effect	Plausible publication factors	Dose-response gradient	Importance	Certainty
1	Case-control study (WHO 1998)	Dates of data collection: February 1, 1989 to January 31, 1993 (in some cases until	Very serious risk of bias[A]	No[B]	Not applicable	No	No	No	No	9. Critical	Very low

		January 31, 1995).									

[A]The quality of the evidence is degraded by two levels, given that it presents a high risk of bias. It presents confusion bias (they do not present association measures adjusted for possible confounding variables and outcomes) and measurement bias, given the high risk of memory bias, since the exposures were obtained through interviews, increasing the risk of differential misclassification. , since cases have a greater probability of remembering the exposures compared to controls.

[B]It does not present sources of indirect evidence (differences in the population (applicability), differences in the intervention (applicability), differences in the outcomes measured (alternative outcomes) or indirect comparisons)

[C]The quality of the evidence is degraded in three levels, given that the optimal size of information is 143,024 patients and has a total of 942 patients. Additionally, the confidence interval is wide and crosses the line of no effect.

[D]The quality of the evidence is degraded in three levels, given that the optimal size of information is 30418050 patients and has a total of 138 patients. Additionally, the confidence interval is wide and crosses the line of no effect.

**Should progestin-only pills be used compared to no progestin-only pills in women of childbearing age with high blood pressure?**											
**Ischemic and hemorrhagic cerebrovascular event**											
Number of studies	Study design	Follow-up duration	Risk of bias	Indirect evidence	Imprecision	Publication bias	Big effect	Plausible publication factors	Dose-response gradient	Importance	Certainty
1	Case-control study (WHO 1998)	Dates of data collection: February 1, 1989 to January 31, 1993 (in some cases until January 31, 1995).	Very serious risk of bias[A]	No[B]	Very serious[C]	No	No	No	No	9. Critical	Very low
Acute myocardial infarction											

Number of studies	Study design	Follow-up duration	Risk of bias	Indirect evidence	Imprecision	Publication bias	Big effect	Plausible publication factors	Dose-response gradient	Importance	Certainty
1	Case-control study (WHO 1998)	Dates of data collection: February 1, 1989 to January 31, 1993 (in some cases until January 31, 1995).	Very serious risk of bias[A]	No[B]	Extremely serious[D]	No	No	No	No	9. Critical	Very low
Venous thromboembolism											
Number of studies	Study design	Follow-up duration	Risk of bias	Indirect evidence	Imprecision	Publication bias	Big effect	Plausible publication factors	Dose-response gradient	Importance	Certainty
1	Case-control study (WHO 1998)	Dates of data collection: February 1, 1989 to January 31, 1993 (in some cases until January 31, 1995).	Very serious risk of bias[A]	No[B]	Extremely serious[E]	No	No	No	No	9. Critical	Very low

[A]The quality of the evidence is degraded by two levels, given that it presents a high risk of bias. It presents confusion bias (they do not present association measures adjusted for possible confounding variables and outcomes), and measurement bias, given the high risk of memory bias, since the exposures were obtained through interviews, increasing the risk of misclassification. differential, since cases have a greater probability of remembering the exposures compared to controls.

[B]It does not present sources of indirect evidence (differences in the population (applicability), differences in the intervention (applicability), differences in the outcomes measured (alternative outcomes) or indirect comparisons).

[C]The quality of the evidence is degraded by two levels, given that the optimal information size is 23,050 patients and has a total of 960 patients; additionally, the interval crosses the line of no effect.

[D]The quality of the evidence is degraded in three levels, given that the optimal size of information is 9948 patients and has a total of 139 patients. Additionally, the confidence interval is wide and crosses the line of no effect.

[E]The quality of the evidence is degraded in three levels, given that the optimal size of information is 145,736 patients and has a total of 135 patients. Additionally, the confidence interval is wide and crosses the line of no effect.

**Should progestin-only injectable contraceptives be used compared to not using progestin-only injectable contraceptives in women of childbearing age with high blood pressure?**											
**Ischemic and hemorrhagic cerebrovascular event**											
Number of studies	Study design	Follow-up duration	Risk of bias	Indirect evidence	Imprecision	Publication bias	Big effect	Plausible publication factors	Dose-response gradient	Importance	Certainty
1	Case-control study (WHO 1998)	Dates of data collection: February 1, 1989 to January 31, 1993 (in some cases until January 31, 1995).	Very serious risk of bias[A]	No[B]	Extremely serious[C]	No	No	No	No	9. Critical	Very low
**Acute myocardial infarction**											
Number of studies	Study design	Follow-up duration	Risk of bias	Indirect evidence	Imprecision	Publication bias	Big effect	Plausible publication factors	Dose-response gradient	Importance	Certainty
1	Case-control study (WHO 1998)	Dates of data collection: February 1, 1989 to January 31, 1993 (in some cases until January 31, 1995).	Very serious risk of bias[A]	No[B]	Very serious[D]	No	No	No	No	9. Critical	Very low
**Venous thromboembolism**											
Number of studies	Study design	Follow-up duration	Risk of bias	Indirect evidence	Imprecision	Publication bias	Big effect	Plausible publication factors	Dose-response gradient	Importance	Certainty

1	Case-control study (WHO 1998)	Dates of data collection: February 1, 1989 to January 31, 1993 (in some cases until January 31, 1995).	Very serious risk of bias[A]	No[B]	Extreme ly serious[E]	No	No	No	No	9. Critical	Very low

[A]The quality of the evidence is degraded by two levels, given that it presents a high risk of bias. It presents confusion bias (they do not present association measures adjusted for possible confounding variables and outcomes), and measurement bias, given the high risk of memory bias, since the exposures were obtained through interviews, increasing the risk of misclassification. differential, since cases have a greater probability of remembering the exposures compared to controls.

[B]It does not present sources of indirect evidence (differences in the population (applicability), differences in the intervention (applicability), differences in the outcomes measured (alternative outcomes) or indirect comparisons).

[C]The quality of the evidence is degraded in three levels, given that the optimal size of information is 302,884 patients and has a total of 944 patients. Additionally, the confidence interval is wide and crosses the line of no effect.

[D]The data provided by the authors do not allow a calculation of the optimal size of information for this outcome.

[E]The quality of the evidence is degraded in three levels, given that the optimal size of information is 339,126 patients and has a total of 133 patients. Additionally, the confidence interval is wide and crosses the line of no effect.

**Should combined oral contraceptives or progestin-only pills be used compared to not using combined oral contraceptives or progestin-only pills in women of childbearing age with high blood pressure?**											
**Ischemic and hemorrhagic cerebrovascular event**											
Number of studies	Study design	Follow-up duration	Risk of bias	Indirect evidence	Imprecision	Publication bias	Big effect	Plausible publication factors	Dose-response gradient	Importance	Certainty
1	Case-control study (Hannaford 1994)	Dates of data collection: 1968-1990.	Very serious risk of bias[A]	No[B]	Very serious[C]	No	No	No	No	9. Critical	Very low
**Peripheral arterial disease**											

Number of studies	Study design	Follow-up duration	Risk of bias	Indirect evidence	Imprecision	Publication bias	Big effect	Plausible publication factors	Dose-response gradient	Importance	Certainty
1	Case-control study (Van Den Bosch 2003)	Data collection dates: January 19990 to October 1995.	Very serious risk of bias[A]	No[B]	Very serious[D]	No	No	No	No	9. Critical	Very low

[A]The quality of the evidence is degraded by two levels, given that it presents a high risk of bias. It presents confusion bias (they do not present association measures adjusted for the possible confounding variables and the outcome) and measurement bias, given the high risk of memory bias, since the exposures were obtained by asking the exposures prior to the day of the event, increasing the risk of differential misclassification, as cases are more likely to remember exposures compared to controls.

[B]It does not present sources of indirect evidence (differences in the population (applicability), differences in the intervention (applicability), differences in the outcomes measured (alternative outcomes) or indirect comparisons).

[C]The quality of the evidence is degraded by two levels, given that the optimal information size is 1834 patients and has a total of 117 patients, and the confidence interval crosses the line of no effect.

[D]The quality of the evidence is degraded by two levels, given that the optimal information size is 10,642 patients and has a total of 98 patients, and the confidence interval crosses the line of no effect.
