# Supplementary material for: Safety and effectiveness of hormonal vs non-hormonal or no contraception in women with hypertension and future fertility desire: A broad-scope systematic review
Source: PLoS One. 2026 Mar 31;21(3):e0345959. doi: 10.1371/journal.pone.0345959 (PMC13038026; doi:10.1371/journal.pone.0345959)
Supplement: S15 Appendix — (PDF) [file pone.0345959.s015.pdf]

O. Appendix S15: 2x2 tables of the included case-control studies

Case-control studies:

- **Combined oral contraceptives:**

o **Primary outcomes**

▪ **Ischemic cerebrovascular event**

Collaborative 1975

	Cases	Controls	Total
Exposed	38	23	61
Not exposed	54	129	183
Total	92	152	244

WHO 1996b

	Cases	Controls	Total
Exposed	26	13	39
Not exposed	150	115	265
Total	176	128	304

Heinemann 1998

	Cases	Controls	Total
Exposed	9	14	23
Not exposed	22	20	42
Total	31	34	65

▪ **Hemorrhagic cerebrovascular event**

Collaborative 1975

	Cases	Controls	Total
Exposed	35	23	58
Not exposed	120	129	249
Total	155	152	307

WHO 1996a

	Cases	Controls	Total
Exposed	44	16	60
Not exposed	295	177	472
Total	339	193	532

▪ **Acute myocardial infarction**

WHO 1997

	Cases	Controls	Total
Exposed	27	6	33
Not exposed	87	55	142
Total	114	61	175

o **Secondary outcomes**

▪ **Venous thromboembolism**

There is not all the data to fill out the 2X2 table.

- **Combined injectable contraceptive:**

o **Primary outcomes**

▪ **Ischemic and hemorrhagic cerebrovascular event**

WHO 1998

	Cases	Controls	Total
Exposed	2	1	3
Not exposed	571	368	939
Total	573	369	942

▪ **Acute myocardial infarction**

WHO 1998

	Cases	Controls	Total
Exposed	1	0	1
Not exposed	84	53	137
Total	85	53	138

- **Secondary outcomes**

- **Venous thromboembolism**

	Cases	Controls	Total
Exposed	0	0	0
Not exposed	41	91	132
Total	41	91	132

- **Progestin-only pill:**

- **Primary outcomes**

- **Ischemic and hemorrhagic cerebrovascular event**

WHO 1998

	Cases	Controls	Total
Exposed	14	7	21
Not exposed	571	368	939
Total	585	375	960

- **Acute myocardial infarction**

WHO 1998

	Cases	Controls	Total
Exposed	1	1	2
Not exposed	84	53	137
Total	85	54	139

- **Secondary outcomes**

- **Venous thromboembolism**

	Cases	Controls	Total
Exposed	1	2	3
Not exposed	41	91	132
Total	42	93	135

- **Progestin-only injectables:**

- **Primary outcomes**

- **Ischemic and hemorrhagic cerebrovascular event**

WHO 1998

	Cases	Controls	Total
Exposed	5	0	5
Not exposed	571	368	939
Total	576	368	944

- **Acute myocardial infarction**

WHO 1998

	Cases	Controls	Total
Exposed	0	0	0
Not exposed	84	53	137
Total	84	53	137

- **Secondary outcomes**
- **Venous thromboembolism**

	Cases	Controls	Total
Exposed	0	1	1
Not exposed	41	91	132
Total	41	92	133

- **Combined oral contraceptives and progestin-only pills:**
- **Primary outcomes**
- **Ischemic cerebrovascular event**

Kemmeren 2002

	Cases	Controls	Total
Exposed	16	19	35
Not exposed	32	36	68
Total	48	55	103

Lidegaard 1993 and Lidegaard 1995

	Cases	Controls	Total
Exposed	18	4	22
Not exposed	50	52	102

Total	68	56	124

- **Ischemic and hemorrhagic cerebrovascular event**

Hannaford 1994

	Cases	Controls	Total
Exposed	21	23	44
Not exposed	30	43	73
Total	51	66	117

- **Acute myocardial infarction**

Croft 1989

	Cases	Controls	Total
Exposed	5	8	13
Not exposed	34	46	80
Total	39	54	93

Tanis 2001

	Cases	Controls	Total
Exposed	24	19	43
Not exposed	35	36	71
Total	59	55	114

- **Secondary outcomes**

- **Peripheral arterial disease**

Van Den Bosch 2003

	Cases	Controls	Total
Exposed	16	19	35
Not exposed	27	36	63
Total	43	55	98
