# Supplementary material for: Safety and effectiveness of hormonal vs non-hormonal or no contraception in women with hypertension and future fertility desire: A broad-scope systematic review
Source: PLoS One. 2026 Mar 31;21(3):e0345959. doi: 10.1371/journal.pone.0345959 (PMC13038026; doi:10.1371/journal.pone.0345959)
Supplement: S16 Appendix — (PDF) [file pone.0345959.s016.pdf]

P. Appendix S16: Synthesis of outcomes related to the use of combined oral contraceptives using the vote counting method

**Outcome**	**Study type and description**	**Number of hypertensive participants**	**Result**			**Certainty of the evidence**	**Interpretation of the results**
			**In favor**	**Against**	**Does not differentiate**		
Prevention of unwanted pregnancies.	1 intervention cohort study. (de Morais 2014 [90]).	They had 56 hypertensive women, 30 exposed and 26 unexposed.			Unwanted pregnancies occurred in none of the groups.	Very low.	The evidence is very uncertain about the effect Combined oral contraceptives may reduce, increase, or have little or no effect.
Ischemic CVD	3 case-control studies<br><br>(Collaborative group for the study of stroke in young women 1975 [84], Heinemann 1998 [76], WHO 1996b [88])	For this outcome, among the 3 studies, there were 613 hypertensive patients: 299 cases (73 exposed and 226 not exposed) and 314 controls (50 exposed and 264 not exposed).<br><br>*Collaborative group for the study of stroke in young women 1975*: 244 hypertensive: 92 cases (38 exposed,	A study (Collaborative group 1975 [84]) suggests that "current" use of oral contraceptives could be positively associated with the presence of ischemic CVD in hypertensive women. *Collaborative group for the Study of Stroke in*		Two studies (WHO 1996b [88]), Heinemann 1998 [76] found that "current" use of combined oral contraceptives could be positively, negatively or have no association with the presence of ischemic CVD. *WHO 1996b: OR crude: 1,53 (IC 95% 0,75-3,11)*	Very low	One study suggests there may be an increased risk and the other two suggest there may be no difference. The evidence is very uncertain on the effect of COC on ischemic stroke. Combined oral contraceptives could reduce, increase, or

Outcome	Study type and description	Number of hypertensive participants	Result			Certainty of the evidence	Interpretation of the results
			In favor	Against	Does not differentiate		
		54 not exposed), 152 controls (23 exposed, 129 not exposed).<br><br>*WHO 1996b*: 304 hypertensive patients (176 cases (26 exposed, 150 not exposed), 128 controls (13 exposed, 115 not exposed).<br><br>*Heinemann 1998*: 65 hypertensive patients (31 cases (9 exposed, 22 not exposed), 34 controls (14 exposed, 20 not exposed)	*Young Women 1975: OR crude: 3,95 (IC 95% 2,15-7,25)*.		*Heinenmann 1998: OR crude: 0,58 (IC 95% 0,21-1,64)]*.		have little or no effect.
Hemorrhagic CVD	2 case-control studies (Collaborative group for the study of stroke in young women 1975 [84], WHO 1996a [85])	Between the 2 studies, there were 839 hypertensive patients: 494 cases (79 exposed and 415 unexposed) and 345 controls (39 exposed and 306 unexposed).	The pooled estimator suggests that the likelihood of having been exposed to combined oral contraceptives among women who had hemorrhagic			Low	Studies suggest that OCP use in hypertensive women may increase the hemorrhagic stroke.

Outcome	Study type and description	Number of hypertensive participants	Result			Certainty of the evidence	Interpretation of the results
			**In favor**	**Against**	**Does not differentiate**		
			CVD was greater than among those who did not have the outcome. *Meta-analysis: Pooled OR: 1.64 (95% CI 1.08-2.5)*				
Acute myocardial infarction	1 case-control study (WHO 1997 [82])<br><br>1 case series (Bounhoure 2008 [92])	In the case-control study there were 175 hypertensive patients, 114 cases (27 exposed, 87 unexposed) and 61 controls (6 exposed and 55 unexposed).<br><br>In the case series there were 12 cases of AMI among oral contraceptive users, three of them were hypertensive women.	This study suggests that the likelihood of having been exposed to combined oral contraceptives among women who experienced acute myocardial infarction was greater than among those who did not have the outcome. *WHO 1997: OR crude: 2,85 (IC95% 1,10-8,93)*			Very low	The evidence is very uncertain about the effect of combined oral contraceptives on the presence of AMI in hypertensive women.<br><br>Combined oral contraceptives could reduce, increase, or have little or no effect.

Outcome	Study type and description	Number of hypertensive participants	Result			Certainty of the evidence	Interpretation of the results
			In favor	Against	Does not differentiate		
			Among the 12 women exposed to oral contraceptives who presented with AMI in the case series, 3 were hypertensive.			Very low	
Venous thromboembolism	1 case-control study (WHO 1995) [79]	For this outcome, the study included 69 cases with a first event of venous thromboembolism in hypertensive women and 133 hypertensive controls. There is no data on exposed and non-exposed cases.	It was not possible to calculate the crude OR with the available data.	It was not possible to calculate the crude OR with the available data.	It was not possible to calculate the crude OR with the available data.	Very low	Combined oral contraceptives may reduce, increase, or have little or no effect .
Worsening of baseline condition: SBP increase	1 cohort study<br><br>de Morais 2014 [90]	In the cohort study there were 56 hypertensive women (exposed: n: 30, unexposed: n: 26).			No changes were identified at 6 months from the start of the study in SBP in users and non-users of combined oral contraceptives.	Very low	Combined oral contraceptives may reduce, increase, or have little or no effect on SBP values in hypertensive women.

Outcome	Study type and description	Number of hypertensive participants	Result			Certainty of the evidence	Interpretation of the results
			In favor	Against	Does not differentiate		
Worsening of baseline condition: increased DBP	1 cohort study<br><br>de Morais 2014 [90]	In the cohort study there were 56 hypertensive women (exposed: n: 30, unexposed: n: 26).			No changes were identified at 6 months from the start of the study in DBP in users and non-users of combined oral contraceptives.	Very low	Combined oral contraceptives may reduce, increase, or have little or no effect on DBP in hypertensive women.
Worsening of the baseline condition: increased daytime SBP	1 cohort study<br><br>by Rossi 2014 [91]	In the cohort study there were 65 hypertensive women (40 exposed and 25 unexposed).			We did not find differences 6 months after the start of the study in daytime SBP values in women users and non-users of combined oral contraceptives.	Very low	Combined oral contraceptives may reduce, increase, or have little or no effect on daytime SBP in hypertensive women.

Outcome	Study type and description	Number of hypertensive participants	Result			Certainty of the evidence	Interpretation of the results
			In favor	Against	Does not differentiate		
Worsening of the baseline condition: increased nocturnal SBP	1 cohort study<br><br>by Rossi 2014 [91]	In the cohort study there were 65 hypertensive women (40 exposed and 25 unexposed).		They found that, 6 months after the beginning of the study, women who used oral contraceptives showed a decrease in nocturnal SBP values.	No differences were found 6 months after the start of the study in nocturnal SBP values in non-users of oral contraceptives.	Very low	Combined oral contraceptives may reduce, increase, or have little or no effect on nocturnal SBP in hypertensive women.
Worsening of baseline condition: increased daytime DBP	1 cohort study<br><br>De Rossi 2014 [91]	In the cohort study there were 65 hypertensive women (40 exposed and 25 unexposed).			No differences were found 6 months after the start of the study in daytime DBP values in women users and non-users of combined oral contraceptives.	Very low	Combined oral contraceptives may reduce, increase, or have little or no effect on daytime DBP in hypertensive women.

Outcome	Study type and description	Number of hypertensive participants	Result			Certainty of the evidence	Interpretation of the results
			In favor	Against	Does not differentiate		
Worsening of the underlying condition: increased nocturnal DBP	1 cohort study<br><br>de Rossi 2014 [91]	In the cohort study there were 65 hypertensive women (40 exposed and 25 unexposed).			No differences were found 6 months after the start of the study in nocturnal DBP values in women users and non-users of combined oral contraceptives.	Very low	Combined oral contraceptives may reduce, increase, or have little or no effect on nocturnal DBP in hypertensive women.
Increase in BMI	2 cohort studies<br><br>de Morais 2014 [90] and de Rossi 2014 [91]	In one of the studies [90] They had 56 hypertensive women (exposed: n: 30, not exposed: n: 26) and in the other study [91] They included 65 hypertensive women (exposed: n: 40, unexposed: n: 25).<br><br>Between the two studies they had 121 hypertensive women (exposed n: 70, unexposed n: 51)		They found [90] a decrease in BMI 6 months after the start of the study in users of combined oral contraceptives.	They found no differences in BMI 6 months after the start of the study in non-users of oral contraceptives [90].<br>In the other study [91] They found no differences in BMI in either users or non-users of combined oral contraceptives 6 months after the start of the study.	Very low	Combined oral contraceptives may reduce, increase, or have little or no effect on BMI in hypertensive women.

Outcome	Study type and description	Number of hypertensive participants	Result			Certainty of the evidence	Interpretation of the results
			In favor	Against	Does not differentiate		
Increased abdominal perimeter	1 cohort study<br><br>de Morais 2014 [90]	In this study they had 56 hypertensive women (exposed: n: 30, unexposed: n: 26).			They found no differences between abdominal circumference values 6 months after the start of the study in users and non-users of combined oral contraceptives.	Very low	Combined oral contraceptives may reduce, increase, or have little or no effect on the abdominal circumference of hypertensive women.
Alteration of kidney function tests	1 cohort study<br><br>de Morais 2014 [90]	In this study they had 56 hypertensive women (exposed: n: 30, unexposed: n: 26).			They found no differences in serum creatinine values in women who used oral contraceptives and in women who did not use oral contraceptives 6 months after the start of the study.	Very low	Combined oral contraceptives may reduce, increase, or have little or no effect on kidney function tests in hypertensive women.
Alteration in total cholesterol levels (increase)	2 cohort studies<br><br>de Morais 2014 [90] and de Rossi 2014 [91]	In one of the studies [90], they had 56 hypertensive women (exposed: n: 30, not exposed: n: 26) and in the other study [91] they had 65 women			In none of the studies were changes found in serum total cholesterol levels either in women using oral contraceptives or in women not	Very low	Combined oral contraceptives may reduce, increase, or have little or no effect on serum total cholesterol levels in

Outcome	Study type and description	Number of hypertensive participants	Result			Certainty of the evidence	Interpretation of the results
			In favor	Against	Does not differentiate		
		(exposed: n: 40, not exposed: n: 25).<br><br>Between the two studies they had 121 hypertensive women (exposed n: 70, unexposed n: 51)			using oral contraceptives 6 months after the start of the study.		hypertensive women.
Alteration of LDL cholesterol levels (increase)	2 cohort studies<br><br>de Morais 2014 [90] and de Rossi 2014 [91]	In one of the studies [90] they had 56 hypertensive women (exposed: n: 30, not exposed: n: 26) and in the other study [91], they had 65 women (exposed: n: 40, not exposed: n: 25).<br><br>Between the two studies they had 121 hypertensive women (exposed n: 70, unexposed n: 51)			In none of the studies were differences found in serum LDL cholesterol levels 6 months after starting the study in women users and non-users of combined oral contraceptives.	Very low	Combined oral contraceptives may reduce, increase, or have little or no effect on serum LDL cholesterol levels in hypertensive women.
Alteration in HDL cholesterol levels (decrease)	2 cohort studies<br><br>de Morais 2014 [90] and	In one of the studies [90], they had 56 hypertensive women (exposed: n: 30, not exposed: n: 26) and in the other			In none of the studies were differences found in serum HDL cholesterol levels in women users	Very low	Combined oral contraceptives may reduce, increase, or have little or no effect on serum

Outcome	Study type and description	Number of hypertensive participants	Result			Certainty of the evidence	Interpretation of the results
			In favor	Against	Does not differentiate		
	de Rossi 2014 [91]	study [91], they had 65 women (exposed: n: 40, not exposed: n: 25).<br><br>Between the two studies they had 121 hypertensive women (exposed n: 70, unexposed n: 51)			and non-users of combined oral contraceptives 6 months after starting the study.		HDL cholesterol levels in hypertensive women.
Alteration in triglyceride levels (increase)	2 cohort studies<br><br>de Morais 2014 [90] and de Rossi 2014 [91]	In one of the studies [90], they had 56 hypertensive women (exposed: n: 30, not exposed: n: 26) and in the other study [91], they had 65 women (exposed: n: 40, not exposed: n: 25).<br><br>Between the two studies they had 121 hypertensive women (exposed n: 70, unexposed n: 51)			In none of the studies were differences found in serum triglyceride levels in women users and non-users of combined oral contraceptives 6 months after the start of the study.	Very low	Combined oral contraceptives may reduce, increase, or have little or no effect on serum triglyceride levels in hypertensive women.
