# Supplementary material for: Safety and effectiveness of hormonal vs non-hormonal or no contraception in women with hypertension and future fertility desire: A broad-scope systematic review
Source: PLoS One. 2026 Mar 31;21(3):e0345959. doi: 10.1371/journal.pone.0345959 (PMC13038026; doi:10.1371/journal.pone.0345959)
Supplement: S17 Appendix — (PDF) [file pone.0345959.s017.pdf]

Q. Appendix S17. Narrative synthesis of continuous outcomes for combined oral contraceptives

Outcome	Studies	Findings
Worsening of the underlying condition	Two cohort studies, de Morais 2014 [90] and de Rossi 2014 [91]	In the Morais 2014 study [90], they found there were no changes in systolic or diastolic blood pressure in users and non-users of combined oral contraceptives (Oral contraceptive users (n = 30): mean SBP +-Standard error (SE): initial value: 127.8+- 2.1mmHg and at 6 months: 126.6+-2.5mmHg (p value: 0.57); DBP+-SE: initial value: 83.9+-1.3mmHg and at 6 months: 83.7+-1.8mmHg (p value: 0.93) Non-users of oral contraceptives (n = 26): mean of SBP +-SE: initial value: 129.0+-2.5mmHg and after 6 months: 130.3+- 2.4mmHg (p value: 0.70); mean DBP+- SE: initial value: 87.6+-1.9mmHg and at 6 months: 87.0+-1.4mmHg (p value: 0.57)).<br><br>In the Rossi 2014 study [91], they found there were no differences between baseline and 6-month values of daytime systolic and diastolic blood pressure in women exposed to the use of combined oral contraceptives and in women not exposed to combined oral contraceptives (mean daytime SBP +- SE: women users of oral contraceptives: baseline: 128.22+-1.66mmHg and at 6 months: 124.11 +-1.45mmHg (p value: 0.197); women who are not users of oral contraceptives: baseline: 130.48+- 2.4mmHg and at 6 months: 127.10+-2.59mmHg (p value: 0.20)). Mean daytime DBP+-SE: women using oral contraceptives: baseline: 82.51+-1.56mmHg and at 6 months: 81.56+-1.51mmHg (p value: 0.495); women who are not users of oral contraceptives: Baseline: 84.81+-1.86mmHg and at 6 months : 82.52+-2.15mmHg (p value: 0.28)). However, they found a statistical difference between the baseline measurement of nocturnal systolic blood pressure among women using oral contraceptives (mean nocturnal SBP+-SE: women using combined oral contraceptives: baseline: 114.5+-1, 79mmHg and at 6 months: 110.24+.1.77mmHg (p value: 0.032)), but no differences were found between the baseline blood pressure measurement nocturnal systolic blood pressure of women who are not users of oral contraceptives and also of the nocturnal diastolic blood pressure of women who are users and non-users of combined oral contraceptives (mean nocturnal DBP+-SE: women who are not users of oral contraceptives: baseline: 114.02+-2.63mmHg and at 6 months: 113.11+-2.77mmHg (p value: 0.68); nocturnal DBP+-SE: women users of oral contraceptives: baseline: 68.74+-

Outcome	Studies	Findings
		1.56mmHg and at 6 months: 65.34+-1.74mmHg (p value: 0.062); baseline: 67.82+-2.09mmHg and at 6 months: 69.13+-1.96mmHg (p value: 0.5)).
Increase in body mass index	Two cohort studies, de Morais 2014 [90] and de Rossi 2014 [91]	In the Morais 2014 study [90], they found a decrease in BMI 6 months after starting the study in users of combined oral contraceptives and they did not find differences in BMI 6 months after starting the study in non-users of oral contraceptives (users of oral contraceptives: mean BMI +- SE: initial value: 30.3 ± 0.9Kg/m2 and at 6 months: 29.8 ± 0.9 Kg/m2 (p value: 0.04), Non-users of oral contraceptives: mean BMI+-SE: initial value: 29.0 ± 1.1Kg/m2 and at 6 months: 28.7 ± 1.2Kg/m2 (p value : 0.20)). In Rossi's 2014 study [91], they did not find differences in BMI values in women who used combined oral contraceptives or in women who were not users of combined oral contraceptives (users of oral contraceptives: mean BMI+-SE: initial value: 28.9+-0.78 Kg/m2 and at 6 months: 28.45+-0.79 Kg/m2 (p value: 0.620). oral contraceptives: initial value: 30.79+-1.20 Kg/m2 and after 6 months: 29.01+-1.11Kg/m2 (p value: 0.27)).
Increased abdominal perimeter	A cohort study de Morais 2014 [90]	In this study, they did not find no differences between the abdominal perimeter values 6 months after the start of the study between users and non-users of combined oral contraceptives (mean abdominal perimeter+-SE: initial value: 97.9 ± 2.0cm and 6 months: 97.2 ± 2.0 (p value: 0.66) Non-users of oral contraceptives: initial value: 95.9 ± 2.6cm and a. 6 months: 94.8 ± 2.6cm (p value: 0.76)).
Alteration of kidney function tests	A cohort study de Morais 2014 [90]	In this study, no differences were found in serum creatinine values in women who used oral contraceptives and in non-users of oral contraceptives when comparing the initial creatinine value before the start of the use of oral contraceptives and 6 months after the start. of this study (oral contraceptive users: mean creatinine+-SE value: baseline value: 0.8+-0.0mg/dL and at 6 months: 0.7+-0.0mg/dL (p value: 0.33). Non-users of oral contraceptives: mean creatinine value+-SE: baseline value: 0.8+-0.0mg/dL and at 6 months: 0.7+-0.0mg/dL (p value: 0.41)).
Deterioration of metabolic parameters (total	Two cohort studies	In the Morais 2014 study [90], no significant changes were identified in serum total cholesterol levels in women who used oral contraceptives or in women who were

Outcome	Studies	Findings
cholesterol)	de Morais 2014 [90] and de Rossi 2014 [91]	not users of oral contraceptives at 6 months after starting the study (women who used oral contraceptives: mean total cholesterol (mg/dL) +-SE: initial value: 188.1+-7.9mg/dL and at 6 months: 186.3+-6.9mg/dL (p value: 0.74). Women who are not users of oral contraceptives: mean total cholesterol (mg/dL)+-SE:197.6+-8.6mg/dL and at 6 months:195.9+-10. .9 mg/dL (p value: 0.73)). In Rossi's 2014 study (91), they did not find significant changes in serum levels of total cholesterol in women users of oral contraceptives or in women not users of oral contraceptives 6 months after starting the study (total cholesterol (mg/dL): women users of contraceptives oral: initial value: 189.6+-7.55mg/dL and at 6 months: 196.61+-8.79mg/dL (value p:0.879). Women who are not users of oral contraceptives: initial value: 188.1+-7.9 mg/dL and at 6 months: 186.3+-6.88 mg/dL (p value: 0.74). .
Deterioration of metabolic parameters (LDL cholesterol)	Two cohort studies de Morais 2014 [90] and de Rossi 2014 [91]	In the Morais 2014 study [90], no differences were found in the serum levels of LDL cholesterol 6 months after starting the study in women users and non-users of oral contraceptives (women users of oral contraceptives: mean LDL cholesterol (mg/dL)+-SE: initial value: 109.8+-7.9mg/dL and at 6 months: 110.5+-6.7mg/dL (p value: 0.88). oral contraceptive users: mean LDL cholesterol (mg/dL)+-SE: initial value: 118.1+-9.3mg/dL and at 6 months: 102.7+-11.6mg/dL (p value: 0.27). In de Rossi 2014 [91], no differences were found between the initial values and 6 months after starting the study between women users and non-users of combined oral contraceptives (women users of oral contraceptives: mean LDL cholesterol (mg/dL)+-SE: value initial:108.14+-9.89mg/dL and at 6 months:102.05+-9.82mg/dL (p value:0.486). Women who are not users of oral contraceptives: mean LDL cholesterol (mg/dL)+-SE: initial value: 109.8 6+-7.86mg/dL and at 6 months: 110.5+-6.67mg/dL (p-value:0.88)).
Deterioration of metabolic parameters (HDL cholesterol)	Two cohort studies, de Morais 2014 [90] and de Rossi 2014 [91]	In the Morais 2014 study [90], no significant differences were found in HDL cholesterol levels between the initial measurement and 6 months after the start of the study, both in women who used oral contraceptives and those who did not use them (women oral contraceptive users: mean HDL cholesterol (mg/dL)+-SE: initial value: 51.12+-2.25mg/dL and at 6 months:58.29+-2.35mg/dL (p value:0.06). Women who are not users of oral contraceptives: mean HDL cholesterol (mg/dL)+-

Outcome	Studies	Findings
		SE: initial value:58.2+-2.69mg/dL and at 6 months: 55.6+-2.46mg/dL (p value: 0.46). In the Rossi 2014 [91], they also did not find differences between the initial HDL cholesterol values and those 6 months after the beginning of the study in women users and non-users of oral contraceptives (women users of oral contraceptives: mean HDL cholesterol (mg/dL)+-SE: initial value : 51.12+-2.25mg/dL and at 6 months: 58.29+-2.35mg/dL (p value: 0.06). Women who did not use oral contraceptives: average cholesterol: mean HDL cholesterol (mg/dL)+-SE: initial value: 58.2+-2.69mg/dL and at 6 months: 55.6+-2.46mg/dL (p value: 0.46)).
Deterioration of metabolic parameters (triglycerides)	Two cohort studies<br>de Morais 2014 [90] and de Rossi 2014 [91]	In the Morais 2014 study [90], they did not find differences in initial triglyceride values and 6 months after the start of the study in women who used oral contraceptives or in women who did not use oral contraceptives (women who used oral contraceptives: mean triglycerides (mg/dL) +-SE: initial value: 140.00+-18mg/dL and at 6 months: 160.02+-15.45mg/dL (p value: 0.244) Women who are not users of oral contraceptives: mean triglycerides (mg/dL)+-SE: initial value: 100.2+-9.13mg. /dL and at 6 months: 100.6+-10.5mg/dL (p value: 0.98)). In Rossi's 2014 study [91], they did not find differences in the initial triglyceride values and 6 months after starting the study in women users and non-users of oral contraceptives (women users of oral contraceptives: mean triglycerides (mg/dL)+-SE: initial value: 140.00+-18mg/dL and at 6 months: 160.02+-15.45mg/dL (value p:0.244). Women who are not users of oral contraceptives: mean triglycerides (mg/dL)+-SE: initial value: 100.2+-9.13mg/dL and at 6 months: 100.6+-10. 5mg/dL (p value: 0.98)).
