# Supplementary material for: Safety and effectiveness of hormonal vs non-hormonal or no contraception in women with hypertension and future fertility desire: A broad-scope systematic review
Source: PLoS One. 2026 Mar 31;21(3):e0345959. doi: 10.1371/journal.pone.0345959 (PMC13038026; doi:10.1371/journal.pone.0345959)
Supplement: S18 Appendix — Outcome: hemorrhagic cerebrovascular event. (PDF) [file pone.0345959.s018.pdf]

## R. Appendix S18. ROB-ME Current use of combined oral contraceptives compared with no current use (past use or never used) of combined oral contraceptives in hypertensive women. Outcome: hemorrhagic cerebrovascular event

| Question | Answer |
|---|---|
| 3.1. Were prospectively registered studies or studies identified for a prospective meta-analysis the only type of study eligible for inclusion in the review? | Yes |
| 3.3. If Y/PY to 3.2: Were you likely to have found all eligible studies regardless of their results? | Yes |
| 4.1. Of the studies identified, was there any for which no result was available for inclusion in the meta-analysis, likely because of the P value, magnitude or direction of the result generated? | No |
| 4.3. Of the studies identified, was there any for which it was unclear whether an eligible result was generated? | No |
| 4.5 Do circumstances indicate potential for some eligible studies not being identified because of the P value, magnitude or direction of the results generated? | No |
| Risk of bias judgement | Low |
