# Supplementary material for: Safety and effectiveness of hormonal vs non-hormonal or no contraception in women with hypertension and future fertility desire: A broad-scope systematic review
Source: PLoS One. 2026 Mar 31;21(3):e0345959. doi: 10.1371/journal.pone.0345959 (PMC13038026; doi:10.1371/journal.pone.0345959)
Supplement: S19 Appendix — (PDF) [file pone.0345959.s019.pdf]

S. Appendix S19. Synthesis of results related to the use of the combined contraceptive vaginal ring using the vote counting method

Outcome	Study type and description	Number of participants	Result			Certainty of the evidence	Interpretation of the results
			In favor	Against	Does not differentiate		
Prevention of unwanted pregnancies	1 case series<br><br>Elkik 1986 [93]	Twelve hypertensive women exposed to the combined contraceptive vaginal ring were included.	None of the women included had unwanted pregnancies in the 187 cycles observed.			Very low	The combined contraceptive vaginal ring may reduce, increase, or have little or no effect on unwanted pregnancies in hypertensive women.
Worsening of baseline condition: SBP increase	1 case series<br><br>Elkik 1986 [93]	Twelve hypertensive women exposed to the combined contraceptive vaginal ring were included.			No differences were found in SBP values in women exposed to the vaginal ring in the 12 months of observation.	Very low	The combined contraceptive vaginal ring may reduce, increase, or have little or no effect on the increase in SBP in hypertensive women.
Worsening of baseline condition: increased DBP	1 case series<br><br>Elkik 1986 [93]	Twelve hypertensive women exposed to the combined contraceptive vaginal ring were included.			No differences were found in DBP values in women exposed to the vaginal ring in the 12 months of observation.	Very low	The combined contraceptive vaginal ring may reduce, increase, or have little or no effect on increased DBP in hypertensive women.

Outcome	Study type and description	Number of participants	Result			Certainty of the evidence	Interpretation of the results
			In favor	Against	Does not differentiate		
Alteration in total cholesterol levels (increase)	1 case series<br><br>Elkik 1986 [93]	Twelve hypertensive women exposed to the combined contraceptive vaginal ring were included.		They found a decrease in total cholesterol values in women exposed to the vaginal ring in the 12 months of observation.		Very low	The combined contraceptive vaginal ring may reduce, increase, or have little or no effect on serum total cholesterol concentrations in hypertensive women.
Alteration of LDL cholesterol levels (increase)	1 case series<br><br>Elkik 1986 [93]	Twelve hypertensive women exposed to the combined contraceptive vaginal ring were included.			They found no differences in the 12-month observation period in LDL cholesterol values in women exposed to the vaginal ring.	Very low	The combined contraceptive vaginal ring may reduce, increase, or have little or no effect on serum LDL cholesterol concentrations in hypertensive women.
Alteration in HDL cholesterol levels (decrease)	1 case series<br><br>Elkik 1986 (93)	Twelve hypertensive women exposed to the combined contraceptive vaginal ring were included.	They found a decrease in HDL cholesterol values in women exposed to the vaginal ring in			Very low	The combined contraceptive vaginal ring may reduce, increase, or have little or no effect on serum HDL cholesterol concentrations in hypertensive women.

Outcome	Study type and description	Number of participants	Result			Certainty of the evidence	Interpretation of the results
			In favor	Against	Does not differentiate		
			the 12 months of observation.				
Alteration in triglyceride levels (increase)	1 case series<br><br>Elkik 1986 [93]	Twelve hypertensive women exposed to the combined contraceptive vaginal ring were included.		They found a decrease in triglyceride values in women exposed to the vaginal ring in the 12 months of observation.		Very low	The combined contraceptive vaginal ring may reduce, increase, or have little or no effect on serum triglyceride concentrations in hypertensive women.
