# Supplementary material for: Safety and effectiveness of hormonal vs non-hormonal or no contraception in women with hypertension and future fertility desire: A broad-scope systematic review
Source: PLoS One. 2026 Mar 31;21(3):e0345959. doi: 10.1371/journal.pone.0345959 (PMC13038026; doi:10.1371/journal.pone.0345959)
Supplement: S20 Appendix — (PDF) [file pone.0345959.s020.pdf]

T. Appendix S20: Narrative synthesis of continuous outcomes for vaginal ring

Outcome	Studies	Findings
Worsening of the underlying condition	A case series study<br><br>Elkik 1986 [93]	In this study, they found no significant differences in SBP or DBP compared to the values before starting the use of the combined contraceptive vaginal ring (Systolic and diastolic blood pressure values: control cycles: cycle -1: number of patients: 12: SBP (mmHg)+-SD: 139.7+-15.7; DBP (mmHg)+-SD: 88.5+-15.7; control cycles: cycle 0: number of patients: 12: SBP (mmHg)+-SD: 134.7+-18.4; DBP (mmHg)+SD: 82.4+-11.2; treatment cycles: cycle 1: number of patients: 12: SBP (mmHg)+-SD: 136.1+-14.6; DBP (mmHg)+SD: 85.0+-9.3 treatment cycles: cycle; 2: number of patients: 12: SBP (mmHg)+-SD: 134.1+-18.3; DBP (mmHg)+SD: 82.8+-12.5; patients: 12: SBP (mmHg)+-SD: 130.1+-12.7; DBP (mmHg)+SD: 81.4+-17.7 cycles; treatment: cycle 6: number of patients: 12: SBP (mmHg)+-SD: 136.0+-16.3; DBP (mmHg)+SD: 83.4+-9.7; 9-12: number of patients: 11; SBP (mmHg)+-SD: 133.6+-16.1: DBP (mmHg)+-SD: 82.8+-10.7; recovery cycles 1-2: Number of patients: 11: SBP (mmHg)+-SD: 132.5+-15.6; DBP (mmHg)+SD: 86.9+-13.02) (p value for DBP and SBP at each measurement time compared to cycle 0: not significant (the significance value is not reported))).
Deterioration of metabolic parameters (total cholesterol)	A case series study<br><br>Elkik 1986 [93]	In this study, they found a decrease in total cholesterol values in hypertensive women exposed to the combined contraceptive vaginal ring compared to the values before starting the use of the combined contraceptive vaginal ring (mean total cholesterol (DS) mg/100mL: control (month 0): 182+-30; 1 month: 158+-30; 2 months: 155+-35; 152+-34; 9-12 months: 157+-26 (p<0.01: compared to measurements with month 0).
Deterioration of metabolic parameters (LDL cholesterol)	A case series study<br><br>Elkik 1986 [93]	In this study, they found no differences in the LDL cholesterol concentrations of hypertensive women exposed to the combined contraceptive vaginal ring compared to the values prior to starting the use of the combined contraceptive vaginal ring (mean LDL cholesterol (DS) mg/100mL: control (month 0): 114+-28; 1 month: 107+-26; 2 months: 105+30; 105+-27; 9-12 months: 109+-32 (p: not significant compared to measurements with month 0 (p value not reported)).
Deterioration of metabolic parameters (HDL	A case series study	In this study, they found a decrease in serum HDL cholesterol concentration values in women exposed to the combined contraceptive vaginal ring compared to the values prior to the start of use of the combined contraceptive vaginal ring (mean HDL cholesterol (DS)

Outcome	Studies	Findings
cholesterol)	Elkik 1986 [93]	mg/100mL : Control (month 0): 59+-10; 1 month: 42+-9; 2 months: 41+-6; 9-12 months: 38+-3 (p<0.001 compared measurements with month 0).
Deterioration of metabolic parameters (Triglycerides)	A case series study<br><br>Elkik 1986 [93]	In this study, they found a decrease in serum triglyceride levels in hypertensive women using the combined contraceptive vaginal ring compared to the values prior to the start of use of the combined contraceptive vaginal ring (mean triglycerides (DS) mg/100mL: Control (month 0): 52+-17; 1 month: 38+-8; 2 months: 38+-13; 6 months: 41+-10; 9-12 months: 38+-11 (p<0.05 compared to measurements with month 0)).
