# Supplementary material for: Safety and effectiveness of hormonal vs non-hormonal or no contraception in women with hypertension and future fertility desire: A broad-scope systematic review
Source: PLoS One. 2026 Mar 31;21(3):e0345959. doi: 10.1371/journal.pone.0345959 (PMC13038026; doi:10.1371/journal.pone.0345959)
Supplement: S21 Appendix — (PDF) [file pone.0345959.s021.pdf]

U. **Appendix S21: Synthesis of the results related to the use of the combined injectable contraceptive using vote-counting method**

Outcome	Study type and description	Number of participants	Result			Certainty of the evidence	Interpretation of the results
			In favor	Against	Does not differentiate		
Ischemic and hemorrhagic CVD	1 case-control study<br><br>WHO 1998 [80]	For this outcome, the study included 942 hypertensive women (cases: 573 (exposed: 2, unexposed: 571), controls: 369 (exposed: 1, unexposed: 368)			"Current use" of combined injectable contraceptives may be both negatively and positively associated with the presence of ischemic or hemorrhagic CVD, or may be unrelated to the outcome.<br><br>*WHO 1998: OR crude: 1.29 (IC 95% 0.07-76.24)*	Very low	The use of combined injectables in hypertensive women could be greater, less or have no effect on the presence of ischemic or hemorrhagic CVD, but the evidence is very uncertain.
Acute myocardial infarction	1 case-control study<br><br>WHO 1998 [80]	For this outcome, the study included 138 hypertensive women (cases: 85 (exposed: 1, unexposed: 84), controls: 53 (exposed: 0, unexposed: 53).			"Current use" of combined injectable contraceptives may be both negatively and positively associated with the presence of acute myocardial infarction, or may be unrelated to the outcome.	Very low	The use of combined injectables in hypertensive women could increase, decrease or have no effect on the presence of AMI, but the evidence is very uncertain.

**Outcome**	**Study type and description**	**Number of participants**	**Result**			**Certainty of the evidence**	**Interpretation of the results**
			In favor	Against	Does not differentiate		
					WHO 1998:OR crude: 1.26 (IC 95% 0.04-38.27)		
Venous thromboembolism	1 case-control study<br><br>WHO 1998 [80]	For this outcome, the study included 132 hypertensive women (cases: 41 (exposed: 0, unexposed: 41), controls: 91 (exposed: 0, unexposed: 91)).			In this study there were no cases or controls exposed to this type of contraceptive.	Very low	The use of combined injectables in hypertensive women could increase, decrease or have no effect on the presence of venous thromboembolism, but the evidence is very uncertain.
