# Supplementary material for: Safety and effectiveness of hormonal vs non-hormonal or no contraception in women with hypertension and future fertility desire: A broad-scope systematic review
Source: PLoS One. 2026 Mar 31;21(3):e0345959. doi: 10.1371/journal.pone.0345959 (PMC13038026; doi:10.1371/journal.pone.0345959)
Supplement: S22 Appendix — (PDF) [file pone.0345959.s022.pdf]

V. **Appendix S22. Synthesis of results related to the use of progestin-only pills using the vote counting method**

Outcome	Study type and description	Number of participants	Result			Certainty of the evidence	Interpretation of the results
			In favor	Against	Does not differentiate		
Ischemic and hemorrhagic CVD	1 case-control study<br><br>WHO 1998 [80]	For this outcome, the study included 960 hypertensive women for this outcome (cases: 585 (exposed: 14, non-exposed: 571), controls: 375 (exposed: 7, non-exposed: 368).			"Current" use of progestin-only pills could be either positively, negatively or unrelated to the presence of ischemic or hemorrhagic cerebrovascular events in hypertensive women.<br>*WHO 1998: OR crude: 1.29 (IC 95% 0.48-3.81).*	Very low	The use of progestin-only pills in hypertensive women could increase, decrease or have no effect on the presence of ischemic or hemorrhagic CVD, but the evidence is very uncertain.
Acute myocardial infarction	1 case-control study<br><br>WHO 1998 [80]	For this outcome, the study included 139 hypertensive women (cases: 85 (exposed: 1, unexposed: 84), controls: 54 (exposed: 1,			"Current" use of progestin-only pills could be either positively, negatively or unrelated to the presence of acute myocardial infarction in hypertensive women.	Very low	The use of progestin-only pills in hypertensive women could increase, decrease or have no effect on the presence of AMI, but the evidence is very uncertain.

Outcome	Study type and description	Number of participants	Result			Certainty of the evidence	Interpretation of the results
			In favor	Against	Does not differentiate		
		unexposed: 53).			*WHO 1998: OR crude: 0.63 (IC 95% 0.01-50)*.		
Venous thromboembolism	1 case-control study<br><br>WHO 1998 [80]	For this outcome, the study included 135 hypertensive women (cases: 42 (exposed: 1, unexposed: 41), controls: 93 (exposed: 2, unexposed: 91)).			"Current" use of progestin-only pills could be either positively, negatively or unrelated to the presence of venous thromboembolism in hypertensive women.<br><br>*WHO 1998: OR crude: 1.11 (IC 95% 0.02-21.86).*	Very low	The use of progestin-only pills in hypertensive women could increase, decrease or have no effect on the presence of venous thromboembolism, but the evidence is very uncertain.
