# Supplementary material for: Safety and effectiveness of hormonal vs non-hormonal or no contraception in women with hypertension and future fertility desire: A broad-scope systematic review
Source: PLoS One. 2026 Mar 31;21(3):e0345959. doi: 10.1371/journal.pone.0345959 (PMC13038026; doi:10.1371/journal.pone.0345959)
Supplement: S23 Appendix — (PDF) [file pone.0345959.s023.pdf]

W. **Appendix S23. Synthesis of results related to the use of progestin-only injectables using the vote counting method**

Outcome	Study type and description	Number of participants	Result			Certainty of the evidence	Interpretation of the results
			**In favor**	**Against**	**Does not differentiate**		
Ischemic and hemorrhagic CVD	1 case-control study<br><br>WHO 1998 [80]	For this outcome, the study included 944 hypertensive women (cases: 576 (exposed: 5, unexposed: 571), controls: 368 (exposed: 0, unexposed: 368)			The "current" use of progestin-only injectables could be either positively or negatively associated or not associated with the presence of ischemic or hemorrhagic cerebrovascular events in hypertensive patients.<br><br>*WHO 1998: OR crude: 6.45 (IC 95% 0.35-118.3)*	Very low	The use of progestin-only injectables in hypertensive women could increase, decrease, or have no effect on the presence of ischemic or hemorrhagic CVD, but the evidence is very uncertain.
Acute myocardial infarction	1 case-control study<br><br>WHO 1998 [80]	For this outcome, the study included 137 hypertensive women (cases: 84 (exposed: 0, unexposed: 84), controls: 53 (exposed: 0, unexposed: 53)			In this study, there were no cases or controls exposed to progestin-only injectables.	Very low	The use of progestin-only injectables in hypertensive women could increase, decrease, or have no effect on the presence of AMI, but the evidence is very uncertain.

Outcome	Study type and description	Number of participants	Result			Certainty of the evidence	Interpretation of the results
			In favor	Against	Does not differentiate		
Venous thromboembolism	1 case-control<br><br>WHO 1998 [80]	133 hypertensive women (41 cases (0 exposed, 41 non-exposed), 92 controls (1 exposed, 91 non-exposed)			The "current" use of progestin-only injectable contraceptives could be both positively and negatively associated or not associated with the presence of venous thromboembolism in hypertensive patients.<br>*WHO 1998: OR crude: 1.11 (IC95% 0.04-33.74).*	Very low	The use of progestin-only injectables in hypertensive women could increase, decrease, or have no effect on the presence of venous thromboembolism, but the evidence is very uncertain.
