# Supplementary material for: Safety and effectiveness of hormonal vs non-hormonal or no contraception in women with hypertension and future fertility desire: A broad-scope systematic review
Source: PLoS One. 2026 Mar 31;21(3):e0345959. doi: 10.1371/journal.pone.0345959 (PMC13038026; doi:10.1371/journal.pone.0345959)
Supplement: S24 Appendix — (PDF) [file pone.0345959.s024.pdf]

X. Appendix S24. Synthesis of results related to the use of combined oral contraceptives or progestin-only pills using the vote counting method.

Outcome	Study type and description	Number of participants	Result			Certainty of the evidence	Interpretation of the results
			In favor	Against	Does not differentiate		
Ischemic CVD	2 case-control studies<br><br>Lidegaard 1993 and 1995 [86,87], Kemmeren 2002 [89]	For this outcome, in the Kemmeren 2002 study [89] They included 103 hypertensive women (cases: 48 (exposed: 16, non-exposed: 32), controls: 55 (exposed: 19, non-exposed: 36)).<br><br>A Lidegaard 1993 and 1995 [86,87] They had 124 hypertensive women (cases: 68 (exposed: 18, non-exposed: 50), controls: 56 (exposed: 4, non-exposed: 52)).<br><br>Between the two studies: *Hypertensive*: total n: 227,	In a studio (86,87), it was found that the “current” use of combined oral contraceptives or progestin-only pills could be positively associated with the presence of ischemic CVD.<br><br>*Lidegaard 1993 y 1995: OR: 4.68 (IC 95% 1.48-14.79)*		in a studio (89) It was found that the “current” use of combined oral contraceptives or progestin-only pills could be positively, negatively or have no association with the presence of ischemic CVD.<br><br>*Kemmeren 2002: OR crude: 0.95 (IC 95% 0.42-22.15)*	Very low	COC or progestin-only pills may reduce, increase, or have little or no effect on the presence of ischemic CVD in hypertensive women.<br><br>One study suggests there may be an increased risk and the other suggests there may be no difference. The evidence is very uncertain about the effect of COC or progestin-only pills on ischemic CVD.

Outcome	Study type and description	Number of participants	Result			Certainty of the evidence	Interpretation of the results
			In favor	Against	Does not differentiate		
		cases: n=116, controls: n=111.					
Ischemic and hemorrhagic CVD	1 case-control study<br><br>Hannaford 1994 [78]	For this outcome, the study included 117 hypertensive women (cases: 51 (exposed: 21, unexposed: 30), controls: 66 (exposed: 23, unexposed: 43).			It was found that the “current” use of combined oral contraceptives or progestin-only pills could be positively, negatively or have no association with the presence of ischemic or hemorrhagic CVD.<br><br>*Hannaford 1994: OR crude: 1.31 (IC 95% 0.62-2.78)*	Very low	Combined oral contraceptives or progestin-only pills may reduce, increase, or have little or no effect on the occurrence of ischemic or hemorrhagic cerebrovascular events in hypertensive women.
Acute myocardial infarction	2 case-control studies<br><br>Croft 1989 [77], Tanis 2001 [83]	For this outcome, in Croft 1989 [77] There were 93 hypertensive women (cases: 39 (exposed: 5, unexposed: 34), controls: 54 (exposed: 8, unexposed: 46).<br><br>In Tanis 2001 [83], they had			In the combined association measure, it was found that the “current” use of combined oral contraceptives or progestin-only pills could be positively, negatively or have no association with the presence of	Very low	Combined oral contraceptives or progestin-only pills may reduce, increase, or have little or no effect on the occurrence of acute myocardial infarction in hypertensive women.

Outcome	Study type and description	Number of participants	Result			Certainty of the evidence	Interpretation of the results
			In favor	Against	Does not differentiate		
		114 hypertensive women (cases: 59 (exposed: 24, non-exposed: 35), controls: 55 (exposed: 19, non-exposed: 36).<br><br>Between the two studies: *Hypertensive*: total n=207, cases: n=98, controls: n=109.			acute myocardial infarction.<br><br>*Meta-analysis: Pooled crude OR: 1.15 (95% CI 0.60-2.19)*		The evidence is very uncertain about the effect of the COC or progestin-only pill on the presence of AMI in hypertensive women.
Peripheral arterial disease	1 case-control study<br><br>Van Den Bosch 2003 [81]	For this outcome, in this study they had 98 hypertensive women (cases: 43 (exposed: 16, non-exposed: 27, controls: 55 (exposed: 19, non-exposed: 36).			It was found that "current" use of combined oral contraceptives or progestin-only pills could be positively, negatively, or have no association with the presence of peripheral arterial disease.<br><br>*Van Den Bosch 2003: OR crude: 1.12 (IC95% 0.45-2.79).*	Very low	COC or progestin-only pills may reduce, increase, or have little or no effect on the presence of peripheral arterial disease in hypertensive women.
