# Supplementary material for: Safety and effectiveness of hormonal vs non-hormonal or no contraception in women with hypertension and future fertility desire: A broad-scope systematic review
Source: PLoS One. 2026 Mar 31;21(3):e0345959. doi: 10.1371/journal.pone.0345959 (PMC13038026; doi:10.1371/journal.pone.0345959)
Supplement: S25 Appendix — Outcome: acute myocardial infarction. (PDF) [file pone.0345959.s025.pdf]

Y. Appendix S25. ROB-ME Current use of combined oral contraceptives or progestin-only pills compared with no current use (past use or never used) of combined oral contraceptives or progestin-only pills in hypertensive women. Outcome: acute myocardial infarction

**Question**	**Answer**
3.1. Were prospectively registered studies or studies identified for a prospective meta-analysis the only type of study eligible for inclusion in the review?	Yes
3.3. If Y/PY to 3.2: Were you likely to have found all eligible studies regardless of their results?	Yes
4.1. Of the studies identified, was there any for which no result was available for inclusion in the meta-analysis, likely because of the P value, magnitude or direction of the result generated?	No
4.3. Of the studies identified, was there any for which it was unclear whether an eligible result was generated?	No
4.5 Do circumstances indicate potential for some eligible studies not being identified because of the P value, magnitude or direction of the results generated?	No
Risk of bias judgement	Low
