# Supplementary material for: Safety and effectiveness of hormonal vs non-hormonal or no contraception in women with hypertension and future fertility desire: A broad-scope systematic review
Source: PLoS One. 2026 Mar 31;21(3):e0345959. doi: 10.1371/journal.pone.0345959 (PMC13038026; doi:10.1371/journal.pone.0345959)
Supplement: S26 Appendix — (PDF) [file pone.0345959.s026.pdf]

Z. Appendix S26. Presentation summary

Presentation summarizing the objectives, methods, results, and main conclusions of the systematic review.

The presentation is available in the Zenodo repository:

Losada-Trujillo, N., Estrada -Orozco, K., Velasco-Lancheros, O. J., Ramirez-Vargas, B. A., Burgos-Cardenas, A. J., González-Caicedo, P., Hoyos Bedoya, M. J., & Gaitan-Duarte, H. (2026, marzo 8). Safety and effectiveness of hormonal vs non-hormonal or no contraception in women with hypertension and future fertility desire: a broad-scope systematic review- Presentation summary. Zenodo. https://doi.org/10.5281/zenodo.18906289
