# Supplementary material for: Safety and effectiveness of hormonal vs non-hormonal or no contraception in women with hypertension and future fertility desire: A broad-scope systematic review
Source: PLoS One. 2026 Mar 31;21(3):e0345959. doi: 10.1371/journal.pone.0345959 (PMC13038026; doi:10.1371/journal.pone.0345959)
Supplement: S27 Appendix — (PDF) [file pone.0345959.s027.pdf]

AA. Appendix S27. Articles screened by title and abstract from the databases

Articles screened by title and abstract from the databases during the systematic review, provided in standard reference formats (.bib, .csv, .enw, and .ris).

The dataset is available in the Zenodo repository:

Losada-Trujillo, N., Estrada -Orozco, K., Velasco-Lancheros, O. J., Ramirez-Vargas, B. A., Burgos-Cardenas, Á. J., González-Caicedo, P., Hoyos Bedoya, M. J., & Gaitán-Duarte, H. (2026). Safety and effectiveness of hormonal vs non-hormonal or no contraception in women with hypertension and future fertility desire: a broad-scope systematic review- Articles screened by title and abstract from the databases [Data set]. Zenodo. https://doi.org/10.5281/zenodo.18906040
