# Supplementary material for: Safety and effectiveness of hormonal vs non-hormonal or no contraception in women with hypertension and future fertility desire: A broad-scope systematic review
Source: PLoS One. 2026 Mar 31;21(3):e0345959. doi: 10.1371/journal.pone.0345959 (PMC13038026; doi:10.1371/journal.pone.0345959)
Supplement: Attachment — Submitted filename: Rebuttal Letter.pdf [file pone.0345959.s028.pdf]

Dear Editor and Reviewers

We sincerely thank you for the careful review of our manuscript, entitled: "Safety and effectiveness of hormonal vs non-hormonal or no contraception in women with hypertension and future fertility desire: a broad-scope systematic review". We appreciate the insightful comments and constructive feedback.

Below, we provide a detailed, point by point response to the comments. All changes have been incorporated into the manuscript, and we have carefully reformatted the tables to enhance readability and consistency.

- Tables: All tables, both within the main manuscript and the supplementary material, have been carefully reformatted to enhance their visual presentation, alignment, and clarity. We standardized font size, optimized spacing, and table legends to comply with journal policies. Additionally, we erased the colour used in subheadings to ensure a more fluent and consistent reading.
- Supplementary material: We have improved the readability, organization, and font size of all appendices, to ensure consistency and full compliance with the journal's formatting policies.
- Figures: We reviewed all in text citations to figures within the main manuscript. Figures are now consistently referred as Fig 1, Fig 2, etc., rather than Figure 1, Figure 2, etc. We used the Preflight Analysis and Conversion Engine (PACE) digital diagnostic tool to ensure all figures meet PLOS ONE requirements.
- Abstract: Abstract section was moved after title page as it was suggested by the journal requirements.
- Title page: We adjusted the symbols used to indicate author contributions and removed professional title abbreviations, in accordance with the journal guidelines.
- New version submission: We have submitted to the PLOS ONE platform a revised and corrected version of the manuscript. All recommended adjustments were incorporated, and the cover letter was updated to indicate this submission as a revised manuscript. In accordance with journal requirements, we provided two versions of the manuscript: one with track changes highlighted and another in clean format. Additionally, we included this response letter in the "Response to Reviewers" section, detailing the main changes have been done.

We sincerely thank the Editors and Reviewers once again for their valuable time, thoughtful comments, and constructive suggestions, which have significantly improved our manuscript. We greatly appreciate the opportunity to review our work and hope the changes made fully address the concerns raised.

Sincerely,

Natalia Losada-Trujillo on behalf of all co-authors
